# Synthesis of β‐Thiolated Amino Acids: Investigation Toward the Total Chemical Synthesis of Lasso Peptide Microcin J25

**DOI:** 10.1002/cbic.70452

**Published:** 2026-08-09

**Authors:** Yu‐Ting Hsiao, Bethan L. Heard, Ilia Perov, Marco J. van Belkum, Sorina Chiorean, Kaitlyn Towle, Isaac Antwi, John C. Vederas

**Affiliations:** ^1^ Department of Chemistry University of Alberta Edmonton Canada

**Keywords:** β‐thiol amino acids, disulfide bonds, lasso peptide, Microcin J25, thiazolidine

## Abstract

Microcin J25 (MccJ25) is of special interest due to its “lariat” topology. The lasso architecture is thought to be responsible for its remarkable thermal stability (100°C for 30 min incubation) and potency against Gram‐negative bacteria (i.e., MBC of ~4.75 µM against *E. coli* strain DH5α). This naturally occurring lasso peptide is formed via post‐translational modifications (PTMs), including leader peptide removal and macrolactam cyclization between the N‐terminus and 8‐Glu side chain. Since the discovery of MccJ25 in 1992, synthetic endeavors toward the total chemical synthesis of MccJ25 have remained elusive. Herein, we investigated the chemical synthesis of MccJ25 utilizing β‐thiol amino acids as the chemical tools to prestaple and rigidify the peptide backbone through disulfide linkages. Although the proposed chemical strategy failed to afford the desired lasso configuration, synthetic routes toward Fmoc‐SPPS compatible, stereoselective β‐thiol amino acids via thiazoline derivatives were developed. In addition, chemical/biochemical literature synthesis of MccJ25 and its analogs, as well as the synthesis of β‐thiol amino acids, are summarized within.

## Graphical abstract

Lasso peptide, Microcin J25 (MccJ25), exhibits extreme stability and potent antibacterial activity against Gram‐negative bacteria due to its rigid “lariat” structure. However, this unique topology precludes researchers to achieve the total chemical synthesis. Herein, we investigated the chemical synthetic prestapling strategy toward synthesis of MccJ25 through disulfide linkages. Synthesis of stereoselective β‐thiol amino acids which are amenable to Fmoc solid‐phase peptide synthesis (SPPS) was established. Furthermore, literature synthesis of MccJ25 and its analogs, as well as the synthesis of β‐thiol amino acids, are summarized within.

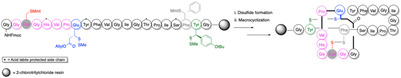

## 1 Introduction

Among the diverse array of naturally occurring ribosomally synthesized and post‐translationally modified peptides (RiPPs), lasso peptides stand out due to their distinctive topology, which gives them a unique “lariat” structure [1, 5]. The threaded conformation of lasso peptides makes them remarkably stable toward various factors, such as high temperatures and proteolytic enzymes [6, 7]. Moreover, the interlocked configuration has been found to exhibit antimicrobial activity via binding atypical molecule targets [8], which launches a new potential to address life‐threatening antimicrobial resistance (AMR) [2]. A recent study by Link's group demonstrated that a lasso peptide can interfere with the protein–protein interaction, which targets thrombosis initiation [9]. The distinguishing physical properties and bioactivity continually capture significant scientific interest to discover, characterize, and engineer naturally occurring lasso peptides [10, 19].

**FIGURE 1:**
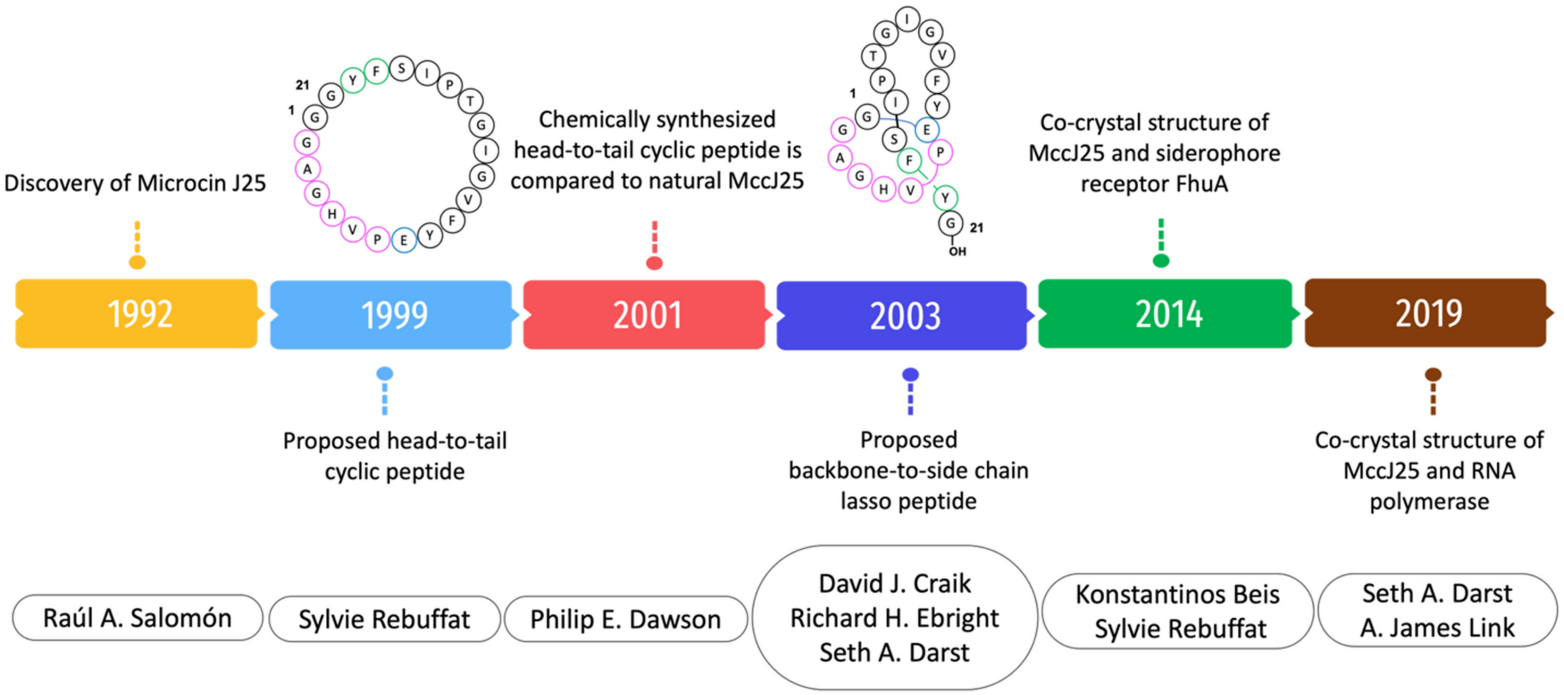
Timeline of MccJ25 elucidation.

One of the most extensively studied lasso peptides, Microcin J25 (MccJ25) [20], was first identified by Salomón in 1992 [21]. MccJ25 is known to possess remarkable bioactivity against Gram‐negative bacteria (i.e., minimum bactericidal concentration (MBC) of 0.01 mg/mL (~4.75 µM) against *Escherichia* *coli* (*E. coli*) strain DH5α) [22]. Figure 1 shows the historical background of MccJ25 since 1992 when Salomón isolated it from *E. coli* and characterized the peptide by composition analysis [21]. The data indicated that MccJ25 may be constituted from 20 amino acids with a molecular weight of *~*2100 Da. Further structural elucidation of the peptide was conducted utilizing Edman degradation, as well as mass spectrometry and NMR in 1999 [23]. MccJ25 was reported as a 21 amino acid, head‐to‐tail cyclic peptide at that time. In 2003, three research groups claimed MccJ25 is a lasso peptide with more comprehensive NMR information and MS/MS fragmentation patterns providing evidence [22, 24, 25]. These publications refuted previous assumptions about the peptide structure. A crystal structure was obtained in complex with the siderophore receptor FhuA from *E. coli* in 2014 [26]. This work not only revealed the lasso peptide structural evidence, but also provided insight into the internalization mechanism of MccJ25 (PDB: 4CU4). Another cocrystal structure of MccJ25 and RNA polymerase from *E. coli* (PDB: 6N60) published later in 2019 by Braffman et al. provided evidence of the ability of MccJ25 to inhibit transcription in bacteria [27].

**FIGURE 2:**
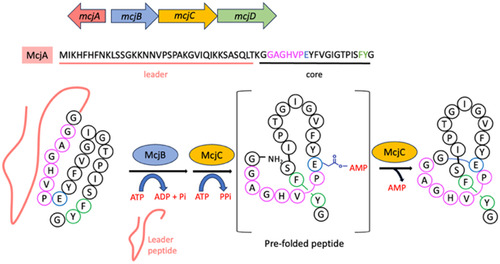
Gene cluster and biosynthesis of MccJ25.

The biosynthetic gene cluster responsible for producing MccJ25 consists of four genes [7, 28]. The 58 amino acid precursor peptide containing leader and core peptide sequences is produced by the *mcjA* gene. The leader peptide is removed by the peptidase McjB, and the lasso cyclase McjC catalyzes macrocyclization. It is believed that the post‐translational modification (PTM) enzymes McjB and McjC play a role in prefolding the core peptide into the correct conformation to form the threaded structure [3, 29]. Lastly, McjD has been reported as an ABC transporter that exports mature MccJ25 (Figure 2) [1, 30].

Although researchers have made efforts to study MccJ25 over decades, total chemical synthesis of MccJ25 remains challenging due to the threaded configuration, a problem that enzymes elegantly solve by prefolding the peptide into the correct conformation. Investigating total chemical synthesis could broaden the research area by allowing for the incorporation of unnatural amino acids to improve bioactivity and facilitate mechanism of action studies. The work on the synthesis of MccJ25 and its analogs will be discussed later in this article.

## 2 Results and Discussion

### 2.1 Structure Elucidation of “Lariat” Topology

**FIGURE 3:**
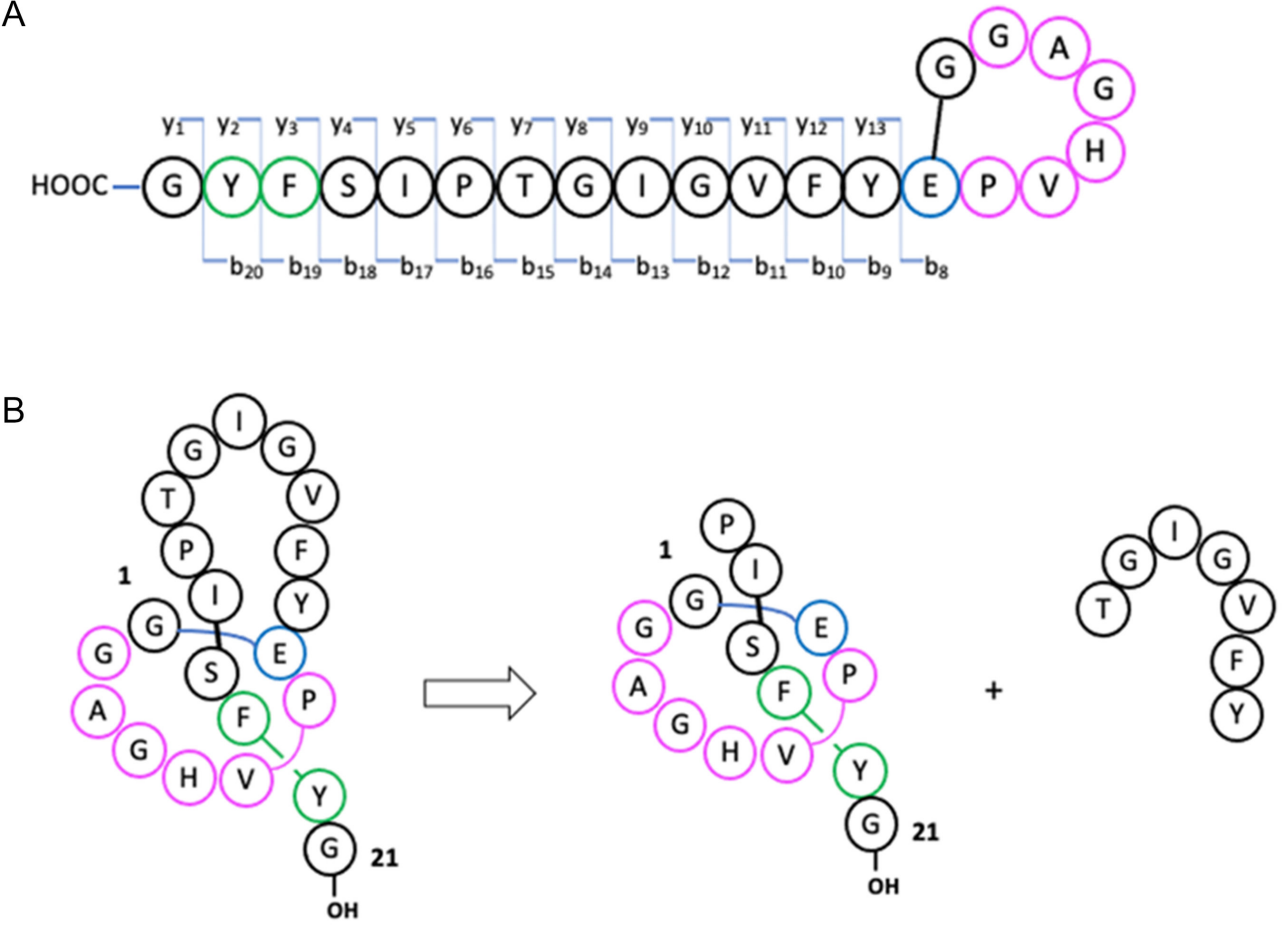
Schematic depiction of the fragmentation of MccJ25 by tandem mass spectrometry.

The threaded lasso topology of MccJ25 is thought to be responsible for its outstanding physical properties. The extremely compact interlocked structure is not only stable to proteolytic cleavage, but the lariat also remains intact in hard ionization conditions [24, 31]. The molecular mass of the unthreaded synthetic MccJ25 analog and the threaded native MccJ25 are identical. Therefore, to distinguish one from another, the unique fragmentation pattern of the rigid lasso peptide after tandem mass spectrometry is important information for structure elucidation [24, 25, 31, 32]. The most distinguishable characteristic of MccJ25 from the loop‐tail peptide (Figure 3A) is the compact region where the steric plugs (Phe_19_ and Tyr_20_) and the macrocycle ring interact. It is reported that the steric bulk of these residues can lock the C‐terminal tail in the macrolactam ring via noncovalent interactions and prevent unthreading even if the loop region is cleaved by mass spectrometry internal fragmentation (Figure 3B). This feature is essential to detect the difference between unthreaded loop‐tail structure and threaded lasso configuration.

### 2.2 Chemical Approaches Toward Synthesis of MccJ25 and Its Analogs

**SCHEME 1:**
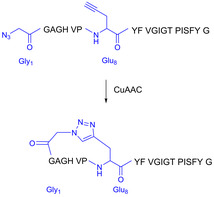
CuAAC cyclization of MccJ25 analog.

Attempts have been made to synthesize MccJ25 using chemical approaches; however, to date, none of them have been successful. In 2010, Link and coworkers intended to synthesize MccJ25 analogs utilizing azide–alkyne click chemistry (Scheme 1) [33]. The sequence of the clickable MccJ25 analog was achieved by replacing Glu_8_ with propargyl glycine, and Gly_21_ was replaced with azidoacetic acid.

After the azide–alkyne cycloaddition (CuAAC) was performed, the cyclic peptide was subjected to mass spectrometry analysis, which suggested the peptide was in a loop‐tail form instead of an interlocked structure. In 2012, Kaur and coworkers synthesized various disulfide‐containing MccJ25 variants and evaluated the antibacterial activities against numerous Gram‐negative pathogens [34]. Although some of the synthetic analogs showed slight activity against Gram‐negative bacteria, the 3D structures of the synthetic peptides were verified by circular dichroism, which suggested the folding behaviors were different from natural MccJ25 (Table 1a). In 2015, Hammami et al. reported similar results by examining the bactericidal activities of several MccJ25‐derived synthetic peptides [35]. Some of the peptides conserved antibacterial activity; the MICs dropped at least 10‐fold compared to native MccJ25, which suggested that the rigid threaded structure might be crucial for the potency of antibacterial agents (Table 1b).

**TABLE 1:** MccJ25 variants from Kaur's and Gomaa's group. Amide and disulfide bonds are colored in pink and blue, respectively.

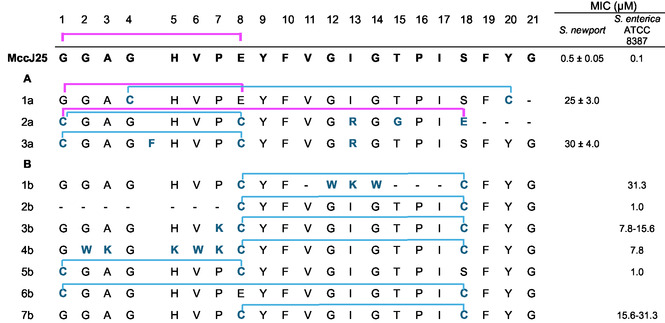

### 2.3 Bioengineered Gene Mutation of MccJ25

To achieve a wide diversity of MccJ25 analogs, single amino acid substitution of MccJ25 except for Glu_8_ was carried out by Pavlova et al. [36] using site‐directed mutagenesis. Around 240 variants were successfully produced, 155 inhibited RNA polymerase, and 77 of these retained antimicrobial activity. Among the active variants, substitution occurred mostly on the hairpin loop region (Phe_10_‐Ser_18_), which suggests the residues on the ring and C‐terminus tail are important for RNA polymerase inhibition. Another gene mutagenesis of MccJ25 was reported by Link's group in 2011 [37]. The study demonstrated that bioengineered gene mutation can widely extend the diversity of MccJ25 variants, and MccJ25 can tolerate multiple substitutions while still maintaining the lasso structure and bioactivity. The limitation of the site‐directed mutagenesis was that only standard amino acids were encoded. Therefore, to expand the variety of MccJ25 variants, Link's group incorporated four meta‐substituted phenylalanine analogs at the selected position in the MccJ25 sequence using a gene code expansion strategy [38]. Promisingly, 15 variants of MccJ25 were produced, and two of the variants were shown to retain nearly wild‐type activity.

### 2.4 Enzymatic Chemical Synthesis of MccJ25 and Its Analogs

In the recent study of MccJ25 by Hartrampf's group, they merged chemical and biological approaches to accomplish expanding diversity of analogs [39]. By utilizing automated fast‐flow peptide synthesis (AFPS), they produced numerous noncanonical substituted precursor peptide McjA analogs. The synthetically modified precursor peptides were then subjected to in vitro transformation with recombinant maturation enzymes. The variants include seven different phenyl substitutions on Tyr_9_ and six heterocyclic derivatives were substituted with Gly_12_ based on the promising result from Link's group. Interestingly, *D*‐amino acids and *N*‐methylated modifications were also tolerated to some extent to be transformed into lasso peptides.

### 2.5 Investigation of Novel Chemical Approaches Toward Total Synthesis of MccJ25 Utilizing β‐Thiolated Amino Acids

**FIGURE 4:**
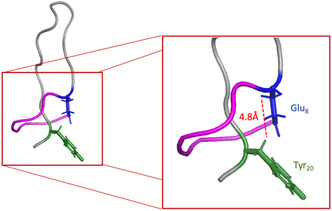
Glu_8_ and Tyr_20_ are in close proximity in 3D model (PDB: 1Q71).

**SCHEME 2:**
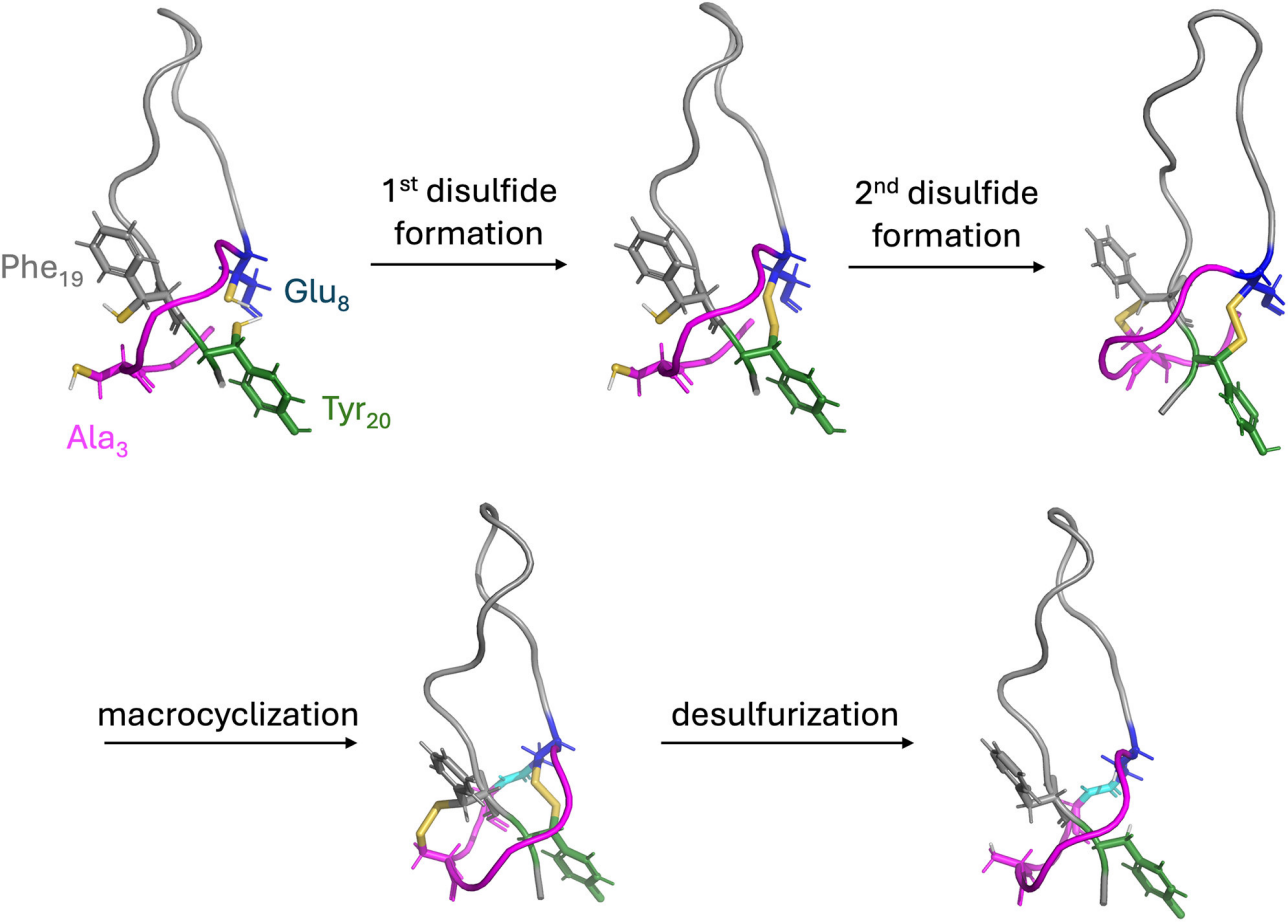
Proposed synthetic route toward MccJ25.

Herein, we focus on the synthesis of β‐thiol‐amino acids, which were incorporated as the synthetic handle in an attempt to synthesize MccJ25. We proposed an approach to prefold the peptide into a hairpin shape using a disulfide bond (yellow) between two amino acids (Glu_8_–Tyr_20_) that are in close proximity based on the 3D structure of MccJ25 (Figure 4, PDB: 1Q71) [24]. In addition, in order to guide the *N*‐terminus of the peptide to cyclize in the desired orientation, another disulfide linker (Ala_3_–Phe_19_) was proposed to rigidify the macrocycle ring region. This rigid synthetic peptide would then be subjected to macrocyclization (cyan) and cyclized into a threaded structure. Finally, desulfurization could be performed to restore proteinogenic amino acid side chains to afford native MccJ25 (Scheme 2).

### 2.6 β‐Thiolated Amino Acid Derivatives as Chemical Tool of Peptide Synthesis

In order to realize our strategy for the synthesis of MccJ25, a number of β‐thiol containing amino acids building blocks were necessary.

**SCHEME 3:**
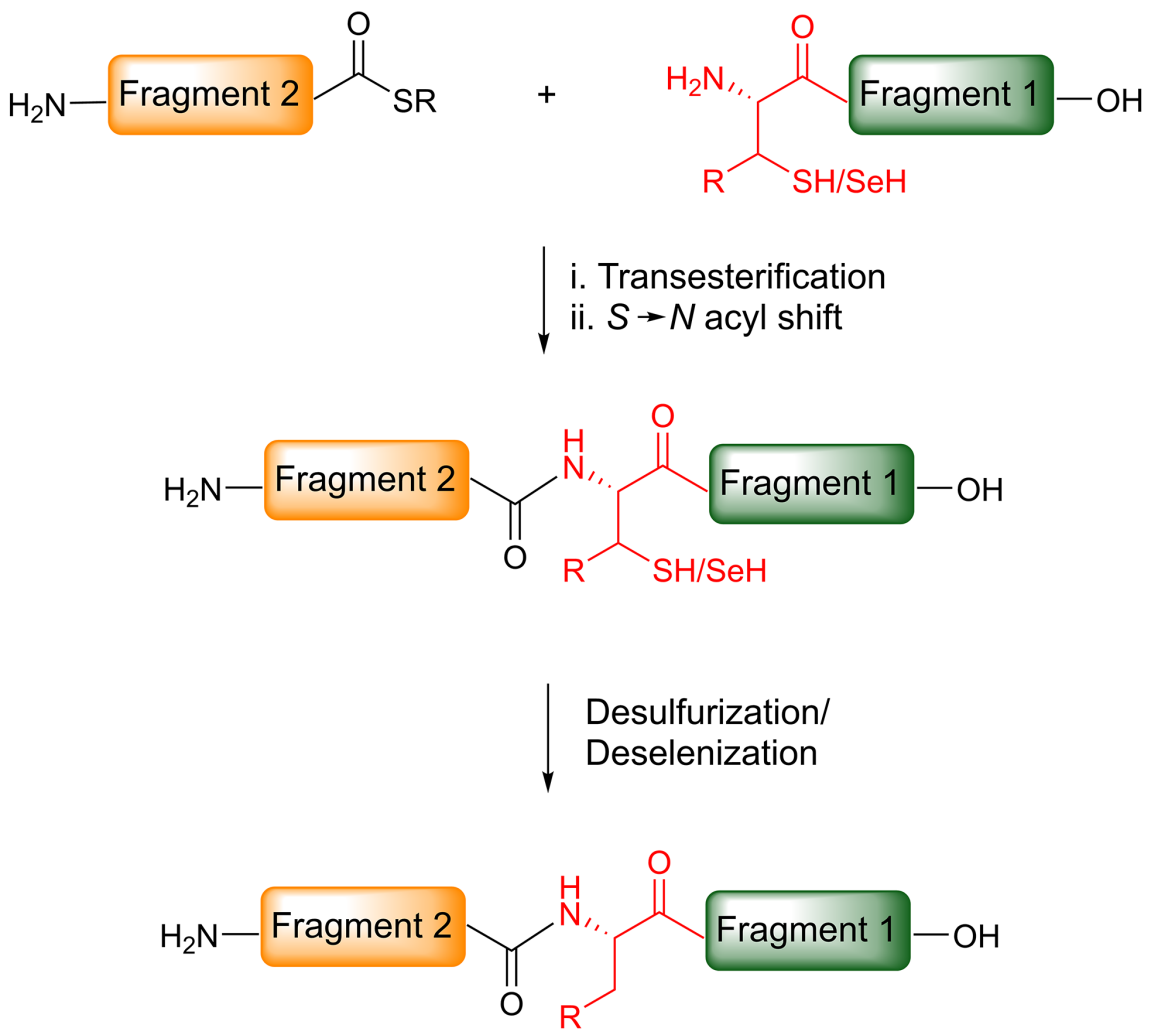
Native chemical ligation.

Sulfur‐containing amino acids, cysteine and methionine, are essential in nature. Moreover, disulfide formation of cysteines in PTM defines the protein shape and geometry structure. Cysteine gained significant interest after native chemical ligation (NCL) was developed by Kent and coworkers [40]. An *N*‐terminal cysteine residue with a *C*‐terminal thioester can undergo transthioesterification and, subsequently, *S*,*N*‐acyl shift that facilitates the formation of a native peptide bond between two peptide fragments. To overcome the limitation of NCL using naturally occurring cysteine residue, synthesis of a number of β‐thiol/selenol‐derived amino acids were developed so they can be utilized as cysteine surrogates [41, 43]. Furthermore, after the formation of the amide bond, free thiol/selenol can undergo traceless dechalcogenation [44] to restore the proteinogenic amino acid side chain (Scheme 3). Approaches toward the synthesis of thiolated/selenolated amino acid derivatives have been summarized comprehensively in the review article by Guan et al. in 2022 [45].

### 2.7 β‐Thiol Arginine

**SCHEME 4:**
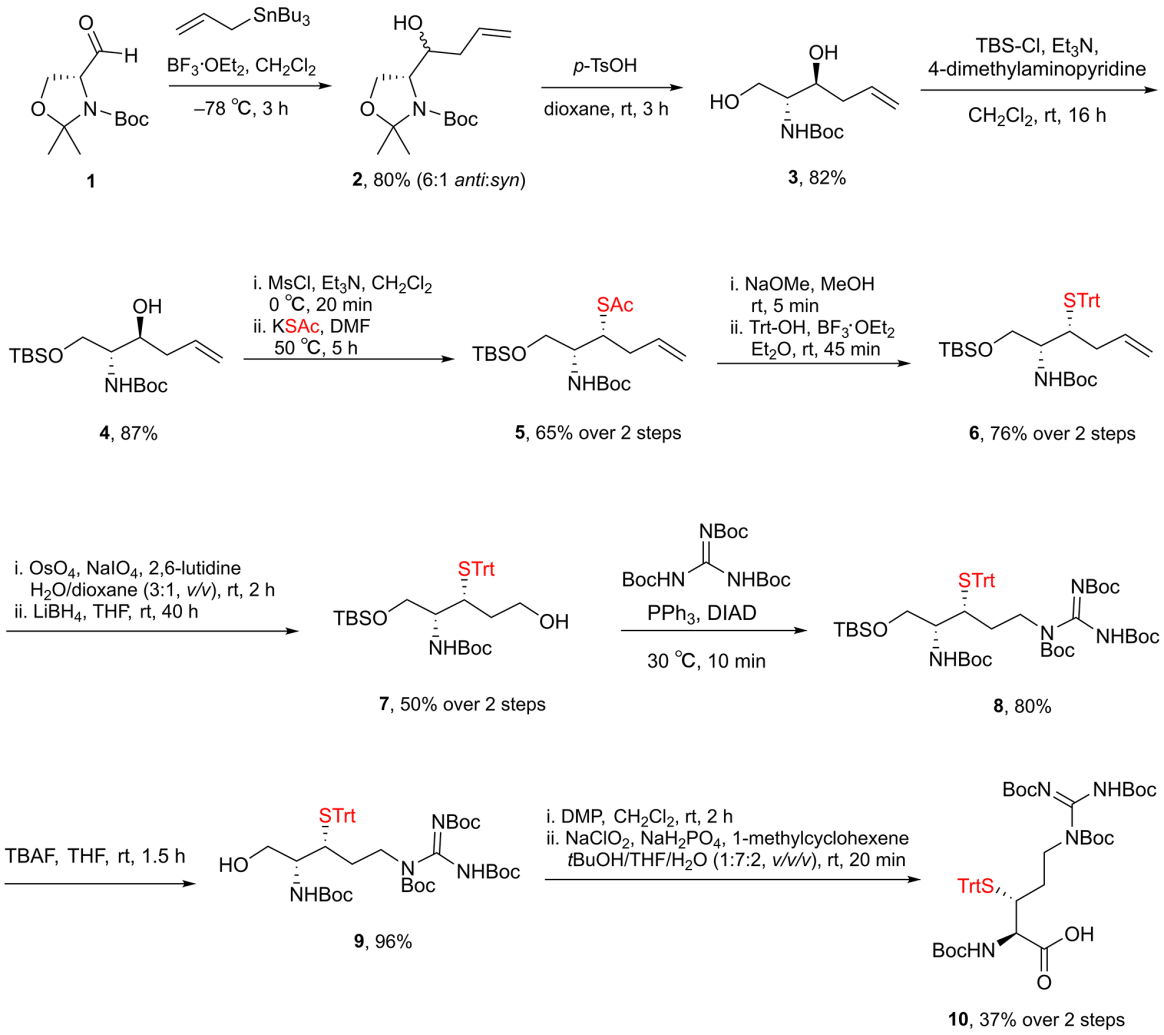
Synthesis of β‐thiol Arg by Payne's group.

In 2013, Malins et al. reported the synthesis of β‐thiol arginine via derivatization of Garner's aldehyde **1**. Thiol was introduced by activating the secondary alcohol of compound **4** through mesylation and subsequent S_*N*_2 conversion with potassium thioacetate. The arginine side chain was obtained by Fukuyama–Mitsunobu reaction with Boc‐protected guanidine to obtain compound **8**. Deprotection and oxidation of primary alcohol of **9** were performed to afford β‐thiol arginine **10**, which was used in the NCL of peptide segments (Scheme 4) [46].

### 2.8 β‐Thiol Phenylalanine

**SCHEME 5:**
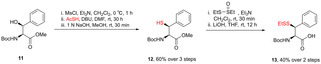
Synthesis of β‐thiol Phe by Crich's group.

**SCHEME 6:**
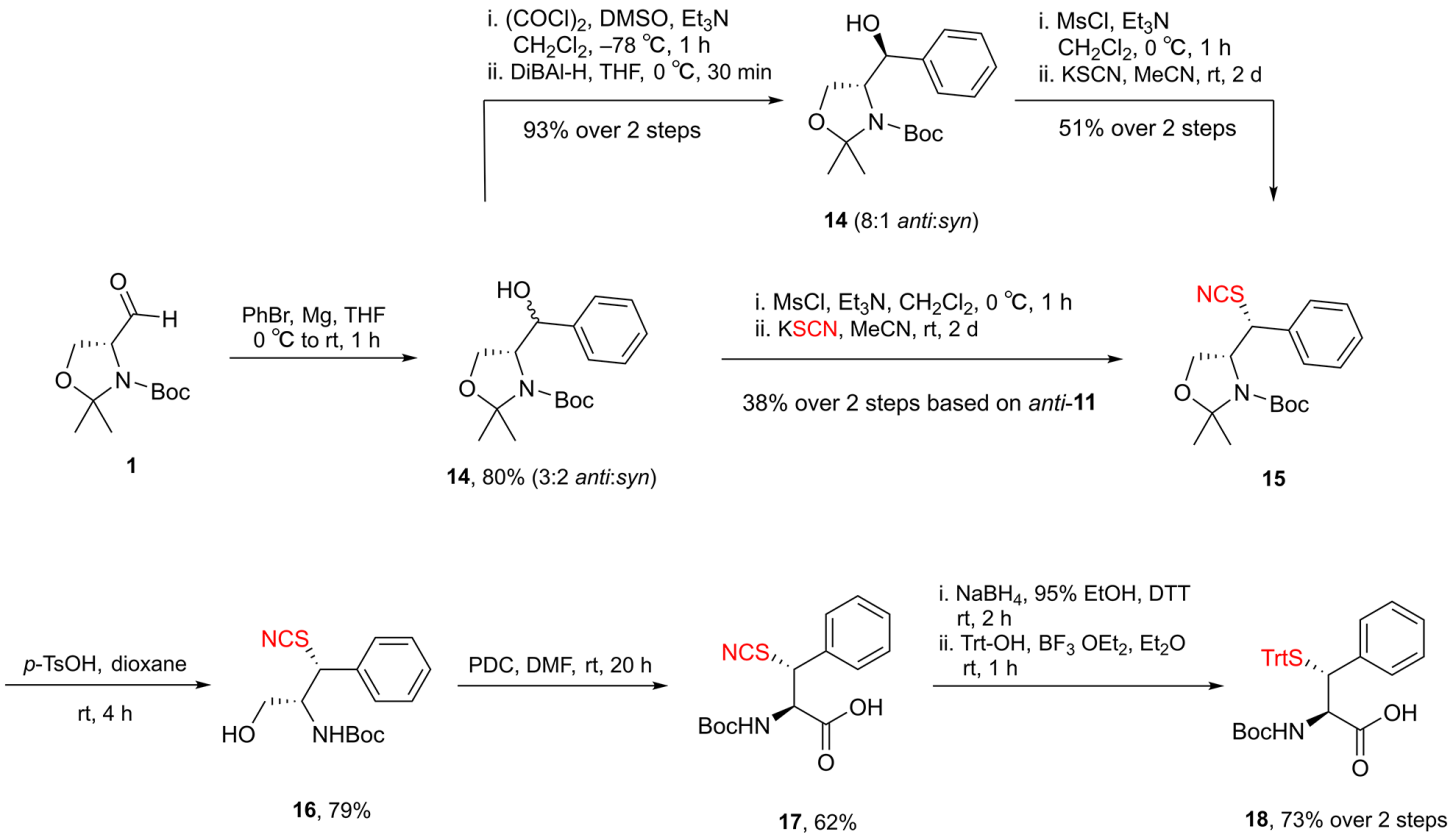
Synthesis of β‐thiol Phe by Payne's group.

In 2007, Crich and coworkers developed a synthetic route toward β‐thiol phenylalanine (Scheme 5) [47] from threo‐β‐hydroxy‐L‐phenylalanine derivative **11**, which can be synthesized by Easton's protocol [48]. Thiol conversion was performed in a similar manner as described to give β‐thiol **12**. It was then protected by reacting with *S*‐ethyl thiomethanesulfonate to afford β‐thiol phenylalanine **13** protected as a disulfide. In 2015, Malins reported the synthetic strategy to produce β‐thiol phenylalanine based on their experience with β‐thiol arginine (Scheme 6) [49].

### 2.9 β‐Thiol Leucine

**SCHEME 7:**
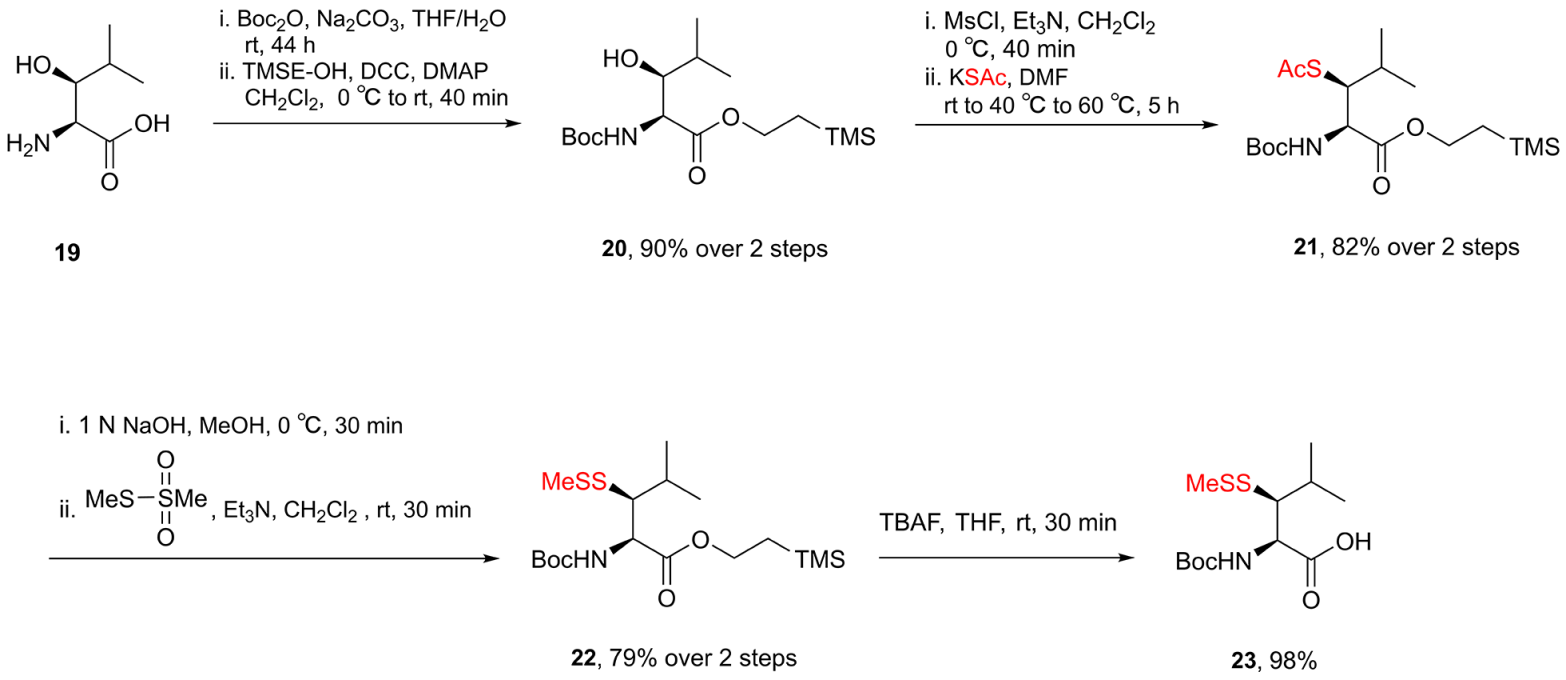
Synthesis of β‐thiol Leu by Danishefsky's group.

In 2010, two research groups published different synthetic routes to synthesize β‐thiol leucine. Both methods relied on relatively expensive (2*S*,3*S*)‐3‐hydroxyleucine **19** as the starting material. The route reported by Danishefsky's group [50] (Scheme 7) was similar to the previously mentioned β‐thiol phenylalanine protocol by Crich.

**SCHEME 8:**
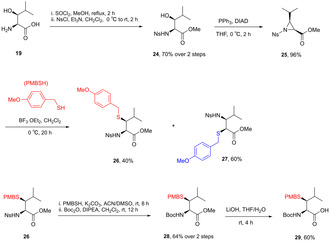
Synthesis of β‐thiol Leu by Brik's group.

On the other hand, Brik's group [51] utilized aziridine intermediate **25** via intramolecular Fukuyama–Mitsunobu reaction to obtain β‐thiol leucine **29** (Scheme 8). The downside of the synthesis involving aziridine intermediate was a substantial formation of the regioisomeric byproduct **27**, which was produced by thiol attack at the *α*‐carbon.

### 2.10 β‐Thiol Aspartic Acid

**SCHEME 9:**
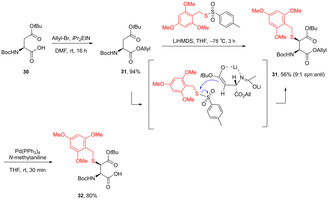
Synthesis of β‐thiol Asp by Payne's group.

**SCHEME 10:**
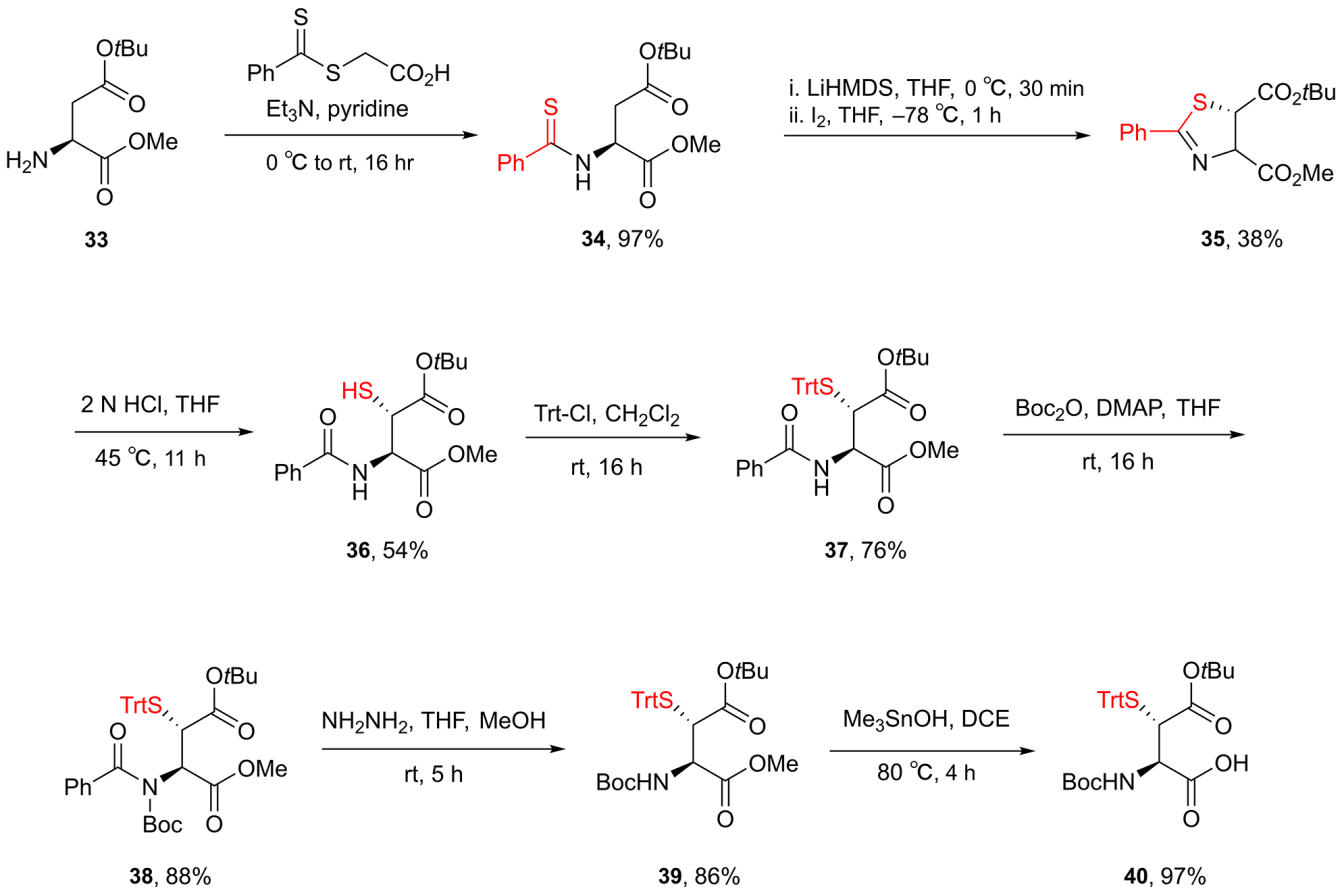
Synthesis of β‐thiol Asp by Tan's group.

In 2013, Payne and coworkers reported electrophilic sulfenylation chemistry to afford β‐thiol aspartate (Scheme 9) [52]. Another synthetic route to β‐thiol aspartate was reported by Tan and coworkers (Scheme 10) [53]. The desired thiol handle was installed via thioamide formation. Thiol rearrangement was achieved through thiazoline intermediate **35**.

### 2.11 β‐Thiol Asparagine

**SCHEME 11:**
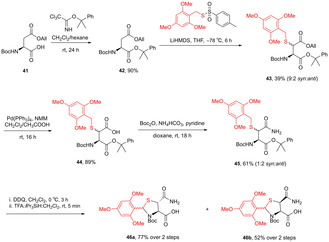
Synthesis of β‐thiol Asn by Payne's group.

In 2015, Payne and coworkers adopted the synthetic β‐thiol aspartate method mentioned above and converted it to a thiol‐derivative of asparagine [54]. Interestingly, they convert the β‐thiol asparagine to the thiazolidine building blocks, **46a** and **46b**, via DDQ‐mediated oxidative cyclization (Scheme 11). The protected thiazolidine asparagine building blocks were efficiently incorporated into solid‐phase peptide synthesis (SPPS) and achieved NCL with peptide thioesters. Furthermore, subsequent desulfurization was conducted to afford the desired 36 amino acids HIV fusion inhibitor enfuvirtide.

**SCHEME 12:**
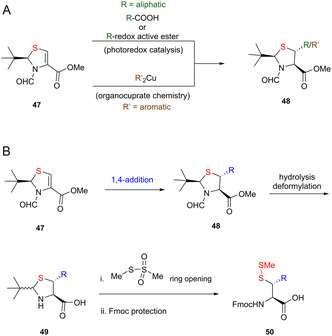
General scheme of β‐thiol amino acids from thiazoline **47**.

In summary, β‐thiol‐derived amino acid building blocks can be synthesized in some cases. The synthetic routes are relatively long and tedious, with poor stereoselectivity. Herein, we report stereoselective synthetic approaches to achieve β‐thiol derived glutamate, phenylalanine, and tyrosine from the readily available thiazolidine derivative **47** (Scheme S1) [55, 56]. Our strategy for the access to these amino acids was to perform various 1,4‐additions to install side‐chain functionality **48**, building on previously published work by Wang's group (Scheme 12A) [56, 58], and then devise a novel and efficient ring opening, followed by Fmoc reprotection that would allow us to directly obtain orthogonally protected amino acids **50** suitable for use in SPPS (Scheme 12B).

**SCHEME 13:**
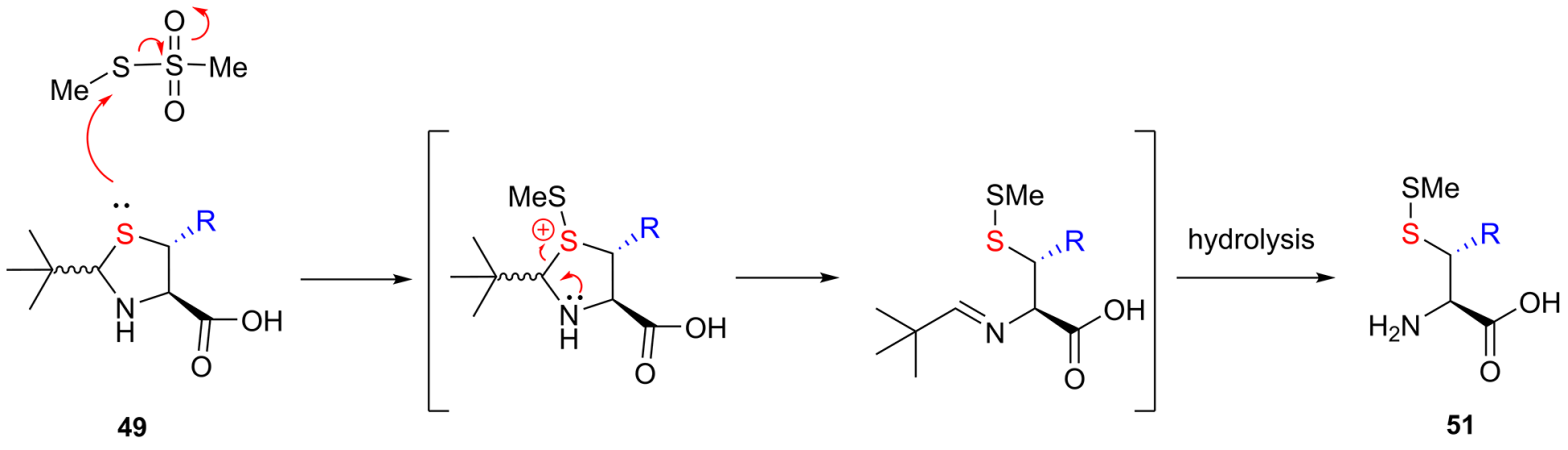
Plausible mechanism of thiazoline deprotection with MMTS.

The methodology developed by Wang and coworkers provides a straightforward approach to thiazolidine‐protected amino acid constructs via either photoredox catalysis (*R* = alkyl) or organocuprate chemistry (*R*’ = aryl), including derivatives of leucine, methionine, lysine, glutamine, and phenylalanine. Although the thiazolidine‐derived amino acids were implemented in SPPS as free amino acids, they could self‐react or multicouple the resin‐bound peptide. In addition, the thiazolidine could only be deprotected at the late stage, when the peptide was cleaved from the resin, which would be useful for peptide fragment ligation but limited its use for elongating the polypeptide chain. Pleasingly, we found that direct reaction of the cyclic thiazolines with *S*‐methyl methanethiosulfonate (MMTS) allowed activation of the sulfide which facilitated ring opening in the same step, leaving the thiol protected as the disulfide, a common sulfur protecting group for SPPS (Scheme 13). We also found that Fmoc protection could be carried out without the challenging isolation of the intermediate free amino acid. This methodology was applied for the synthesis of the required amino acid building blocks.

### 2.12 β‐Thiol Aspartic Acid from Thiazoline Scaffold

**SCHEME 14:**
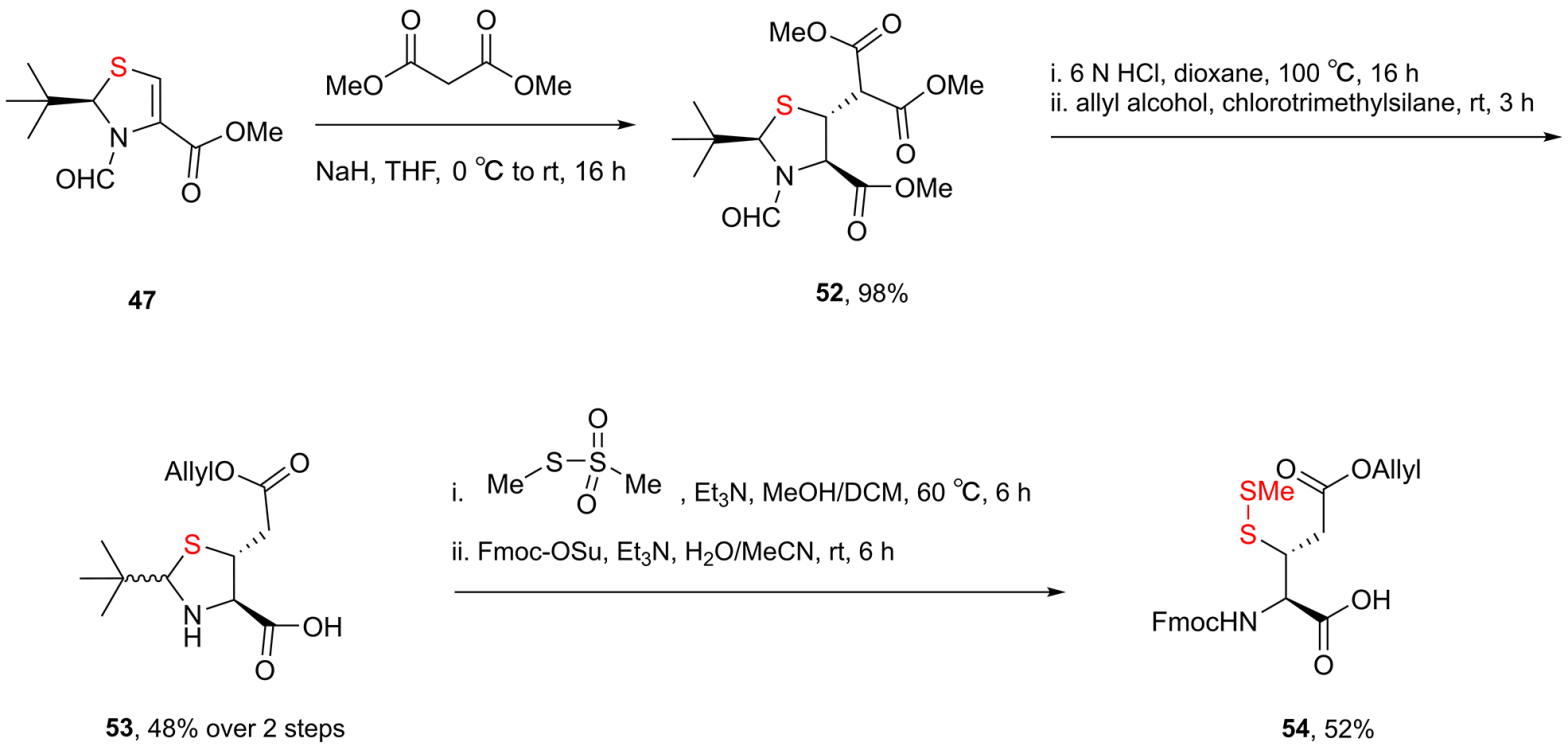
Synthesis of β‐thiol Asp from thiazoline.

For the synthesis of β‐thiol aspartic acid, conjugate addition of dimethyl malonate to the thiazolidine **47** gave the triester **52** in high yield. One pot hydrolysis, Krapcho decarboxylation, and deformylation under acidic conditions followed by selective side chain reprotection of the allyl group afforded compound **53**. Ring opening of thiazolidine was facilitated by a mild electrophilic MMTS reagent. Subsequently, Fmoc protection was carried out to obtain β‐thiol aspartate **54** (Scheme 14).

### 2.13 β‐Thiol Tyrosine From Thiazoline Scaffold

**SCHEME 15:**
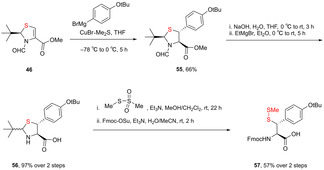
Synthesis of β‐thiol Tyr from thiazoline.

The tyrosine side chain moiety was installed by cuprate chemistry to obtain **55** [57]. In order to hydrolyze the *α*‐methyl ester with *tert*‐butoxy group on the aromatic ring intact, hydrolysis was performed under basic conditions. Subsequent formyl deprotection yielding compound **56** was achieved with a Grignard reagent. Ring opening of thiazolidine and Fmoc protection was conducted using mild electrophilic reagent MMTS as described to afford the desired protected β‐thiol tyrosine **57** (Scheme 15).

### 2.14 β‐Thiol Phenylalanine From Thiazoline Scaffold

**SCHEME 16:**
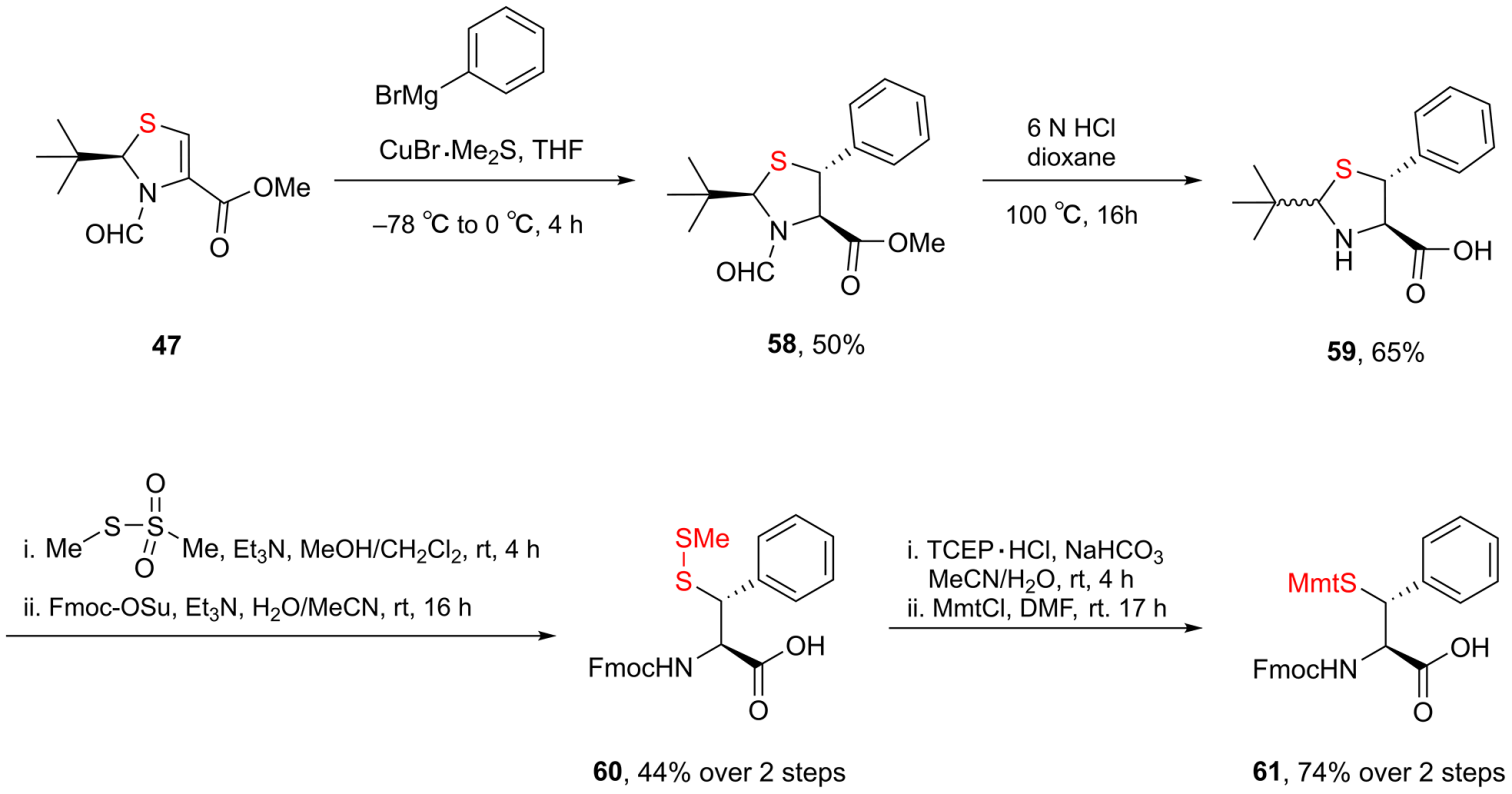
Synthesis of β‐thiol Phe from thiazoline.

Thiazoline **47** underwent cuprate addition to give **58** [57], followed by ester hydrolysis and formyl removal in acidic conditions to obtain thiazolidine **59**. To selectively form two pairs of disulfide bonds in the peptide synthesis, an orthogonal protecting group, monomethoxytrityl (Mmt), was chosen. However, an attempt to ring open thiazolidine directly with Mmt‐Cl was low‐yielding. Therefore, ring opening and Fmoc protection were performed analogously to the synthesis of β‐thiol tyrosine to afford disulfide protected β‐thiol phenylalanine **60**. The protecting group was transformed via reduction with TCEP, and the free thiol was protected with Mmt‐Cl to afford Mmt protected β‐thiol phenylalanine **61** (Scheme 16).

### 2.15 Attempted Total Chemical Synthesis of MccJ25

**SCHEME 17:**
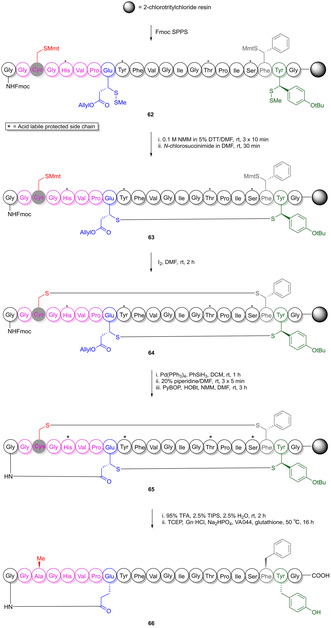
Synthesis of bisdisulfide‐containing MccJ25 analog.

Bisdisulfide‐containing prefolded MccJ25 analog was synthesized via Fmoc‐SPPS protocol using polystyrene 2‐Cl‐Trt chloride resin. Synthetic β‐thiol Tyr **57** was used at residue 20; β‐thiol Phe **61** at residue 19; β‐thiol Glu **54** at residue 8. Mmt‐protected Cys was used at residue 3, which at the later stage of the synthesis can be desulfurized to produce alanine. Once linear peptide **62** was prepared, it was subjected to selective disulfide formation between Glu_8_ and Tyr_20_ via disulfide reduction and subsequent oxidation with *N*‐chlorosuccinimide to obtain **63** [59]. The second disulfide was formed by one‐pot deprotection of Mmt groups and oxidation to link Ala_3_ and Phe_19_ producing peptide **64**. Macrocyclization was performed after allyl and Fmoc deprotections to afford **65**. The cyclized peptide was then cleaved from the resin and subjected to desulfurization conditions to give synthetic MccJ25 peptide **66** (Scheme 17). Structural elucidation of the synthetic peptide was conducted by tandem mass spectrometry; however, there was no evidence of unique fragments of “lariat” topology (Figure 3B) unfortunately indicating that the synthetic peptide had a loop‐tail topology.

**FIGURE 5:**
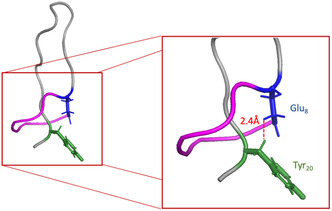
Distance between *S*‐γ‐hydrogen of Glu_8_ and *R‐*β‐hydrogen Tyr_20_ in 3D model (PDB: 1Q71).

**SCHEME 18:**
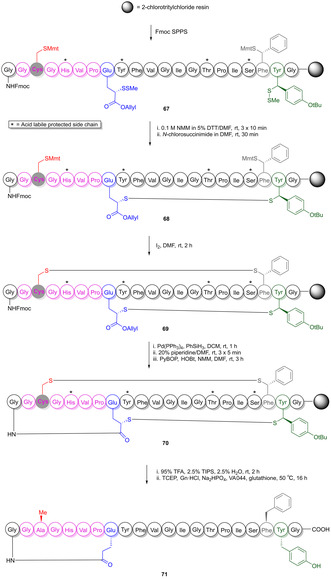
Synthesis of MccJ25 with γ‐thiol Glu.

The proposed chemical strategy was revised to increase the rigidity of the synthetic peptide. The MccJ25 model demonstrated that *S*‐γ‐hydrogen of Glu_8_ is the closest atom to Tyr_20_ residue in the natural peptide structure (Figure 5). Therefore, we proposed that the installation of *S*‐γ‐thiol Glu_8_ instead of the β‐thiol Glu_8_ would help to compress the prefolded hairpin for a higher chance of cyclizing through the C‐terminus. Protected γ‐thiol Glu was synthesized from slightly revised protocol reported by Payne's group [60] (Scheme S2) and installed at residue 8. Revised prefolded MccJ25 analog was synthesized via a similar procedure. The linear peptide with four synthetic thiol‐derived amino acids was made by SPPS, followed by sequential selective disulfide formation. The peptide containing two disulfides was cleaved from the resin, disulfide bonds were reduced, and thiols were desulfurized to produce synthetic MccJ25 **71** (Scheme 18). Unfortunately, the data from tandem mass spectrometry suggested that **71** was not successfully folded into the threaded configuration.

The failure of our methodology to successfully construct the lariat topology could be a result of there being too much conformational freedom around the loop area to undergo the desired cyclization. Further rigidifying the ring and/or the junction could potentially increase the possibility of cyclizing into threaded configuration; however, we are unsure that this method would guarantee the desired threaded topology.

**FIGURE 6:**
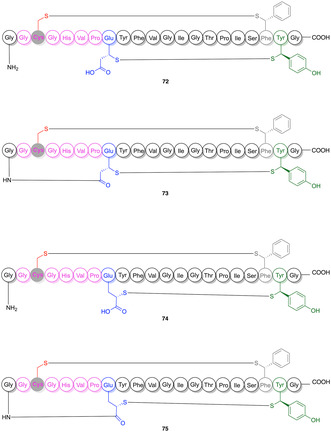
Four bisdisulfide‐containing synthetic MccJ25 analogs for spot‐on‐lawn bioactivity assay.

Four bisdisulfide‐containing synthetic analogs **72–75** (Figure 6) were tested for bioactivity using spot‐on‐lawn assay. To our regret, no activity was observed at 128 µM concentration of each of the synthetic peptides (Figure S1, Supporting Information).

## 3 Conclusion

Chemical synthesis of noncanonical β‐thiol amino acids was summarized in this article. We developed an alternative chemical strategy to synthesize protected β‐thiol Glu, Tyr, and Phe via the thiazoline scaffold. These β‐thiolated amino acids are useful for peptide synthesis applications, including peptide folding via disulfide formation. Additionally, thiazolidine‐derived amino acids can be incorporated as pseudoproline‐like building blocks to minimize polypeptide aggregation during SPPS. On the other hand, the developed relatively mild thiazolidine ring‐opening conditions using MMTS can provide an alternative approach to unmask internal peptide fragments for sequential NCL. The total chemical synthesis of MccJ25 was investigated to determine whether rigidifying the peptide structure via disulfide linkages could favor cyclization of the macrocycle ring around the prefolded hairpin to afford the desired threaded topology. Regretably, the lasso structure remains elusive with the approaches involving disulfide stapling.

## Funding

This study was supported by the Natural Sciences and Engineering Research Council of Canada (RGPIN‐2020‐03894).

## Conflicts of Interest

The authors declare no conflicts of interest.

## Supporting information

Supplementary Material

## Data Availability

The data that supports the findings of this study are available in the Supporting Information of this article.
