# Supplementary material for: Synthesis of β‐Thiolated Amino Acids: Investigation Toward the Total Chemical Synthesis of Lasso Peptide Microcin J25
Source: Chembiochem. 2026 Aug 9;27(15):e70452. doi: 10.1002/cbic.70452 (PMC13453073; doi:10.1002/cbic.70452)
Supplement: Supplementary file 1 [file CBIC-27-e70452-s001.pdf]

Supporting Information

Synthesis of β-Thiolated Amino Acids: Investigation Toward the Total Chemical Synthesis of Lasso Peptide Microcin J25

Yu-Ting Hsiao,[a] Bethan L. Heard,[a] Ilia Perov,[a] Marco J. van Belkum,[a] Sorina Chiorean,[a] Kaitlyn Towle,[a] Isaac Antwi,[a] and John C. Vederas*[a]

[a] Department of Chemistry, University of Alberta, Edmonton, Alberta, Canada T6G 2G2

*To whom correspondence should be addressed

Email: john.vederas@ualberta.ca

1) General Information **5**

1.1 Characterization 5

1.2 Reagents and Solvents 5

1.3 Purification 5

1.4 Peptide Synthesis 6

1.4.1 2-Chlorotrityl Chloride Resin Loading Procedure *6*

1.4.2 General Procedure for Reduced Loading Procedure *6*

1.4.3 General SPPS Procedure *6*

1.4.4 General Test Cleavage or Full Peptide Cleavage Procedure *7*

1.4.5 Single Disulfide Formation *7*

1.4.6 Bis-Disulfide Formation *7*

1.4.7 Allyl/Alloc Deprotection and General Cyclization Procedure *8*

1.4.8 Disulfide Reduction and Desulfurization[1] *8*

1.4.9 General Procedure for Disulfide Mapping Analysis[2] *8*

1.5 Peptide Purification 9

1.5.1 Purification of Standard Closed-Loop / Synthetic Peptide after Desulfurization *9*

1.5.2 Bis-Disulfide Containing Intermediate Peptide Purification *9*

1.5.3 Bis-Disulfide and Macrolactam Ring Containing Intermediate Peptide Purification *9*

1.5.4 Purification of Partially Reduced Nem/Nmm Alkylated Peptide *10*

2) Synthesis of enantiopure thiazolidine derivative 47 [3] **11**

2.1 Methyl (2*R*,4*R*)-2-(*tert*-butyl)-3-formylthiazolidine-4-carboxylate (**S1**) 11

2.2 Methyl (2*R*,4*R*,5*R*)-5-(4-(*tert*-butoxy)phenyl)-2-(*tert*-butyl)-3-formylthiazolidine-4-carboxylate (**47**) 12

3) Synthesis of β-Thiol Aspartic acid from Thiazoline Scaffold **13**

3.1 Dimethyl 2-((2*R*,4*R*,5*R*)-2-(*tert*-butyl)-3-formyl-4-(methoxycarbonyl)thiazolidin-5-yl)malonate (**52**) 13

3.2 (4*R*,5*R*)-5-(2-(Allyloxy)-2-oxoethyl)-2-(*tert*-butyl)thiazolidine-4-carboxylic acid (**53**) 14

3.3 (2*R*,3*R*)-2-((((9*H*-Fluoren-9-yl)methoxy)carbonyl)amino)-5-(allyloxy)-3-(methyldisulfaneyl)-5-oxopentanoic acid (**54**) 15

4) Synthesis of β-Thiol Tyrosine from Thiazoline Scaffold **16**

4.1 Methyl (2*R*,4*R*,5*R*)-5-(4-(*tert*-Butoxy)phenyl)-2-(*tert*-butyl)-3-formylthiazolidine-4-carboxylate (**55**) 16

4.2 (4*R*,5*R*)-5-(4-(*tert*-Butoxy)phenyl)-2-(*tert*-butyl)thiazolidine-4-carboxylic acid (**56**) 17

4.3 (2*R*,3*R*)-2-((((9*H*-Fluoren-9-yl)methoxy)carbonyl)amino)-3-(4-(*tert*-butoxy)phenyl)-3-(methyldisulfaneyl)propanoic acid (**57**) 18

5) Synthesis of β-Thiol Phenylalanine from Thiazoline Scaffold **19**

5.1 Methyl (2*R*,4*R*,5*R*)-2-(*tert*-butyl)-3-formyl-5-phenylthiazolidine-4-carboxylate (**58**) 19

5.2 (4*R*,5*R*)-2-(*tert*-Butyl)-5-phenylthiazolidine-4-carboxylic acid (**59**) 20

5.3 (2*R*,3*R*)-2-((((9*H*-Fluoren-9-yl)methoxy)carbonyl)amino)-3-(((4-methoxyphenyl)-diphenylmethyl)thio)-3-phenylpropanoic acid (**60**) 20

5.4 (2*R*,3*R*)-2-((((9*H*-Fluoren-9-yl)methoxy)carbonyl)amino)-3-(methyldisulfaneyl)-3-phenyl-propanoic acid (**61**) .. 21

6) Synthesis of MccJ25 analog from bis-disulfide/ β -thiol Glu strategy 23

6.1 Disulfide-containing MccJ25 analog with β-thiol-Glu (**65**) 23

6.2 MccJ25 analog from bis-disulfide/ β-thiol-Glu (**66**) 23

7) Synthesis of γ-thiol Glu 24

7.1 Synthetic Scheme of γ-thiol Glu 24

7.2 Boc-Glu(OAll)-O^{t}Bu (**S2**) 25

7.3 *S*-(2,4,6-trimethoxybenzyl)toluenethiosulfonate (**S3**) 25

7.4 Boc-(4*S*-STmob)Glu(OAll)-O^{t}Bu (**S5**) 26

7.5 1-Allyl 5-(*tert*-butyl) (2*S*,4*S*)-4-((*tert*-butoxycarbonyl)amino)-2-(methyldisulfaneyl)-pentanedioate (**S6**) 27

7.6 5-Allyl 1-(*tert*-butyl) (2*S*,4*S*)-2-((((9*H*-fluoren-9-yl)methoxy)carbonyl)amino)-4-(methyl-disulfaneyl)pentanedioate (**S7**) 28

8) Synthesis of MccJ25 analog from bis-disulfide/ γ-thiol Glu strategy 29

8.1 Disulfide-containing MccJ25 analog with γ-thiol-Glu (**70**) 29

8.2 Macrolactam ring and disulfide-containing MccJ25 analog with γ-thiol-Glu (**71**) 29

9) Synthetic MccJ25 analogues purification and characterization 30

9.1 Synthetic loop-tail MccJ25 analog (**S8**)[6] 30

9.2 Synthetic MccJ25 analog from bis-disulfide/ β-thiol-Glu (**66**) 30

9.3 Synthetic MccJ25 analog from bis-disulfide/ γ -thiol-Glu (**71**) 31

10) Synthetic MccJ25 analogues intermediates characterization 31

10.1 Disulfide-containing MccJ25 analog with β-thiol-Glu (**72**) 31

10.2 Macrolactam ring and disulfide-containing MccJ25 analog with β-thiol-Glu (**73**) 33

10.3 Disulfide-containing MccJ25 analog with γ-thiol-Glu (**74**) 34

10.4 Macrolactam ring and disulfide-containing MccJ25 analog with γ-thiol-Glu (**75**) 36

11) Bioactivity of synthetic MccJ25 analogues intermediates 38

12) MS/MS of loop-tail MccJ25 and synthetic MccJ25 analogues 40

13) Determination of disulfide connectivity of synthetic MccJ25 analogues intermediates 41

13.1 Synthesis of Nem/Nmm alkylated MccJ25 analog (**S10**) 41

13.2 MS/MS of partially reduced Nem alkylated MccJ25 analog (**S9**) 42

13.3 MS/MS of Nem/Nmm alkylated MccJ25 analog (**S10**) 44

14) High Temperature ^{1}H NMR (400 MHz, DMSO-d$_6$) of Rotamer 52 46

15) References 47

16) **NMR Spectra** **48**

1) General Information

1.1 Characterization

Nuclear magnetic resonance (NMR) spectra were obtained using an Agilent VNMRS 700 MHz, Agilent/Varian VNMRS 600 MHz, Agilent/Varian VNMRS 500 MHz, Agilent/Varian Inova 500 MHz, Agilent/Varian Inova 400 MHz, or Agilent/Varian DD2 MR 400 MHz spectrometer. For ^{1}H (700, 600, 500, 400 MHz) spectra, δ values were referenced to $CDCl_3$ (7.26 ppm) or CD_3OD (3.31 ppm); and for ^{13}C (175, 150, 125, 100 MHz) spectra, δ values were referenced to $CDCl_3$ (77.06 ppm) or CD_3OD (49.00 ppm) as the solvents. Infrared spectra (IR) were recorded on a Nicolet Magna 750 or a 20SX FT-IR spectrometer. Cast film and thin film refer to the evaporation of a solution on a NaCl or KBr plate. Mass spectra were recorded on a Agilent Technologies 6220 (high resolution electrospray (ESI)) with orthogonal TOF. LC-MS analysis was performed on an Agilent Technologies 6130 LCMS instrument equipped with +ve and –ve ion ESI ionization, and full-scan MS (high-resolution analysis) with two-point lock mass correction operating mode. All MALDI MS were acquired on an Applied Biosystems Voyager Elite MALDI-TOF with delayed extraction in reflectron mode. A two-layer method was used for the matrix using 3,5-dimethoxy-4-hydroxycinnamic acid (sinapinic acid). MS/MS fragmentation was performed on an ThermoScientific Orbitrap Exploris 240 with quadrupole orbitrap.

1.2 Reagents and Solvents

All commercially available reagents and protected amino acids were purchased and used without further purification unless otherwise noted. All the solvents used for reactions were used without further purification unless otherwise noted. Dry solvents refer to solvents freshly distilled over appropriate drying reagents prior to use. Commercially available ACS grade solvents (> 99.0% purity) were used for column chromatography without any further purification.

1.3 Purification

All reactions and fractions from column chromatography were monitored by thin layer chromatography (TLC) using glass plates (5 × 2.5 cm) pre-coated (0.25 mm) with silica gel (normal SiO_2, Merck 60 F254). Visualization of TLC plates was performed by UV fluorescence at 254 nm in

addition to staining by $KMnO_4$ or ninhydrin. Flash chromatography was performed using Merck type 60, 230-400 mesh silica gel at elevated pressures.

1.4 Peptide Synthesis

1.4.1 2-Chlorotrityl Chloride Resin Loading Procedure

A fritted SPPS vessel was used with an argon inlet for reagent and resin bubbling during resin swelling and amino acid couplings. The resin was swelled for 20 minutes in CH_2Cl_2 (10 mL/g resin) with a constant stream of argon bubbling through it. The first C-terminus Fmoc protected amino acid (4 equiv) was loaded onto 2-chlorotrityl chloride resin (0.69 mmol/g) using DIPEA (5 equiv) in CH_2Cl_2 (10 mL/g resin). The resin was bubbled under argon for 2 hours and washed with CH_2Cl_2 (3 × 10 mL). Unreacted sites were capped by a solution of MeOH/DIPEA/DMF (1:1:8, 10 mL) and agitating the mixture for 1 hour bubbling argon through. Resin was washed with DMF (3 × 10 mL) and then CH_2Cl_2 (3 × 10 mL) to give the loaded resin.

1.4.2 General Procedure for Reduced Loading Procedure

The first amino acid loaded resin (0.69 mmol/g) was subjected to Fmoc deprotection by bubbling a solution of 20% piperidine in DMF using argon for 5 minutes (3 × 10 mL). After deprotection, the resin was washed with DMF (3 × 10 mL) and then CH_2Cl_2 (3 × 10 mL). Fmoc protected tyrosine or β-SMmt tyrosine (0.1 mmol/g resin) and Boc-Ala-OH (0.5 mmol/g resin) were pre-activated with HATU (0.6 mmol/g resin), HOAt (0.6 mmol/g resin), DIPEA (10.0 mmol/g resin) in DMF (10 mL/g resin) for 10 minutes. Then the coupling solution was added to the resin and agitated for 3 hours by bubbling argon through. The resin was washed with DMF (3 × 10 mL) and then CH_2Cl_2 (3 × 10 mL). Unreacted sites were capped by a solution of Ac_2O/Pyridine/DMF (1:2:7, 30 mL) and agitating the mixture for 1 hour bubbling argon through. Resin was washed with DMF (3 × 10 mL) and then CH_2Cl_2 (3 × 10 mL) to give the reduced loading resin (0.1 mmol/g).

1.4.3 General SPPS Procedure

All amino acids are of L-configuration unless otherwise stated. Unless noted otherwise, SPPS was performed in fritted glass reaction vessel equipped with an argon inlet for resin/reagent agitation and an outlet for draining. For peptide structures, asterisk sites indicate that the amino acid residue is protected with an acid-labile group (*t*Bu for Ser and Thr and Tyr; Trt for His).

Fmoc deprotection was performed by bubbling a solution of 20% piperidine in DMF using argon for 5 minutes (3 × 10 mL). After deprotection, resin was washed with DMF (3 × 10 mL) and then CH_2Cl_2

(3 × 10 mL). The appropriate Fmoc-protected amino acid to be coupled (5.0 equiv) was pre-activated with HATU (4.9 equiv), HOAt (4.9 equiv), DIPEA (4.9 equiv) in DMF (10 mL) for 10 minutes. Then the coupling solution was added to the resin and agitated for 2 hours by bubbling argon through. The resin was washed with DMF (3 × 10 mL) and then CH_2Cl_2 (3 × 10 mL). Unreacted sites were capped by a solution of Ac_2O/Pyridine/DMF (1:2:7, 30 mL) and agitating the mixture for 20 minutes bubbling argon through. Resin was washed with DMF (3 × 10 mL) and then CH_2Cl_2 (3 × 10 mL).

1.4.4 General Test Cleavage or Full Peptide Cleavage Procedure

Peptide was cleaved from resin using a mixture of TFA:H_2O:TIPS (95:2.5:2.5 v:v:v, 10 mL/g of resin). For testing cleavage, 10 mg of resin was used and cleavage solution was shaken for 10 minutes; for complete peptide cleavage, the cleavage solution was shaken for 16 hours. After cleavage, the solution was filtered through a pipette with a cotton plug and rinsed with CH_2Cl_2 and concentrated under argon. Cold diethyl ether addition caused the peptide to precipitate and the solid peptide was centrifuged and the mother liquor was decanted.

1.4.5 Single Disulfide Formation

SSMe groups on Tyr and Glu residues were reduced from the resin bound peptide using PBu_3 (10.0 equiv) and thiophenol (5.0 equiv) in THF:H_2O (9:1 v:v, 10 mL/g of resin). The solution mixture was shaken at room temperature for 16 hours. After the disulfide was reduced to free thiols, the resin was washed with DMF (3 × 10 mL) and then CH_2Cl_2 (3 × 10 mL), followed by iodine (10.0 equiv) oxidation in DMF (10 mL/g of resin) bubbling through argon for 3 hours. After the reaction time, the resin was washed with DMF (3 × 10 mL) and then CH_2Cl_2 (3 × 10 mL) to afford the disulfide containing peptide.

1.4.6 Bis-Disulfide Formation

SSMe groups on Tyr and Glu residues were reduced from the resin bound peptide using dithiothreitol (5.0% in DMF 10 mL/g of resin) and NMM (0.1 M). The solution mixture was bubbled through argon (3 × 10 min). After the disulfide was reduced to free thiols, the resin was washed with DMF (3 × 10 mL) and then CH_2Cl_2 (3 × 10 mL), Oxidation was performed using *N*-chlorosuccinimide (NCS) (4.0 equiv) in DMF (10 mL/g of resin) bubbling through argon for 30 minutes. After the reaction time, the resin was washed with DMF (3 × 10 mL) and then CH_2Cl_2 (3 × 10 mL). The second disulfide bond was formed between β-Phe and β-Ala with Mmt protecting groups. Deprotection and disulfide formation were performed in one-pot using iodine (10.0 equiv) in DMF (10 mL/g of resin) for 2 hours with a constant stream of argon bubbling through it.

1.4.7 Allyl/Alloc Deprotection and General Cyclization Procedure

Allyl and alloc groups were removed from the resin bound peptide using $Pd(PPh_3)_4$ (2.0 equiv) and $PhSiH_3$ (10 equiv) in DCM (10 mL/g of resin). The reaction vessel was covered in aluminum foil and shaken at room temperature for 1 hour. The resin was washed sequentially with DMF (3 × 10 mL), CH_2Cl_2 (3 × 10 mL), 0.5% sodium diethyldithiocarbamate in DMF (3 × 10 mL), and then again with DMF (3 × 10 mL) and CH_2Cl_2 (3 × 10 mL). The N-terminus Fmoc was then deprotected with 20% piperidine as previously described. The peptide was cyclized by adding a solution of PyBOP (5.0 equiv), HOBt (5.0 equiv) and NMM (10.0 equiv) in DMF (10 mL/g of resin) and bubbling under argon for 3 h. The resin was washed with DMF (3 × 10 mL) and then CH_2Cl_2 (3 × 10 mL).

1.4.8 Disulfide Reduction and Desulfurization[1]

Following peptide cleavage procedure, peptide was then dissolved in minimal volume of ACN (~10 µL/ mg crude peptide). A phosphate buffer (500 mM TCEP·HCl, 6 M Gn·HCl, and 100 mM Na_2HPO_4, 100 µL/ mg crude peptide) was adjusted with 5 M NaOH to pH 7.0. The PBS buffer was then added to the peptide mixture then de-gassed with argon. A radical initiator VA-044 (400 mM) and glutathione (200 mM) were added as solid to the reaction mixture. The reaction was stirred at 50 ℃ for 16 hours. After the reaction was completed, the reaction mixture was concentrated *in vacuo* and redissolved in MeOH for purification.

1.4.9 General Procedure for Disulfide Mapping Analysis[2]

(1) Partial reduction with TCEP

Bis-disulfide peptide dissolved in MeCN:H_2O (1:1 v:v, 2.0 mg/mL) was incubated with an equivalent volume of 20 mM TCEP·HCl, 0.2 M citrate buffer, pH 3.0 at room temperature for 3 minutes. The reaction was stopped by immediate injection to an analytical ZORBAX 300SB-C3 column, eluted with RP-HPLC partially reduced Nem/Nmm alkylated peptide method. Peptide was eluted at 8.50 minutes as monitored using LCMS.

(2) *N*-ethylmaleimide (Nem) alkylation

The partially reduced peptide was collected, and a solution of 60 mM Nem (same volume as collected HPLC fraction) in 0.2 M citrate buffer at pH 3.0 was added at room temperature. The peptide solution was incubated for 2 hours. Alkylated product was purified by RP-HPLC using an analytical ZORBAX 300SB-C3 column, eluted with RP-HPLC partially reduced Nem/Nmm alkylated peptide method. The peptide was eluted at 12.14 minutes and checked by LCMS/MS.

(3) Complete reduction and *N*-methylmaleimide (Nmm) alkylation

The HPLC fraction containing Nem-alkylated peptide was treated with the same volume of 20 mM TCEP·HCl, 0.2 M citrate buffer, pH 3.0 at room temperature for 1 hour. A solution of 60 mM Nem (same volume as collected HPLC fraction) in 0.2 M citrate buffer at pH 3.0 was added at room temperature. The peptide solution was incubated for 2 hours and subjected to LCMS/MS analysis.

1.5 Peptide Purification

1.5.1 Purification of Standard Closed-Loop / Synthetic Peptide after Desulfurization

Crude peptides were purified by semi-preparative RP-HPLC using a Vydac 219TP 5 µm biphenyl column with a flow rate of 5 mL/min. Detection was done at 220, 254 and 280 nm. Purification was conducted with aqueous 0.1% TFA (solvent A) and 0.1% TFA in acetonitrile (solvent B) as eluents. Gradient began with 15% MeCN/85% H_2O (0.1% TFA) for the first 5 min. Gradient ramped up to 33% MeCN over 30 min, then final ramp up to 100% MeCN over the next 1 min and held for 5 min.

1.5.2 Bis-Disulfide Containing Intermediate Peptide Purification

Crude peptides were purified by semi-preparative RP-HPLC using a Vydac 219TP 5 µm biphenyl column with a flow rate of 5 mL/min. Detection was done at 220, 254 and 280 nm. Purification was conducted with aqueous 0.1% TFA (solvent A) and 0.1% TFA in acetonitrile (solvent B) as eluents. Gradient began with 15% MeCN/85% H_2O (0.1% TFA) for the first 5 min. Gradient ramped up to 33% MeCN over 30 min, then final ramp up to 100% MeCN over the next 1 min and held for 5 min. Fractions containing the peptide, as checked by MALDI, were pooled, lyophilized, and re-dissolved in MeOH.

Semi-pure peptides were re-injected and purified by analytical RP-HPLC using a ZORBAX 300SB-C3 column with a flow rate of 1 mL/min. Gradient began with 20% MeCN/80% H_2O (0.1% TFA) for the first 2 min. Gradient ramped up to 38% MeCN over 23 min, then final ramp up to 100% MeCN over the next 1 min and held for 5 min.

1.5.3 Bis-Disulfide and Macrolactam Ring Containing Intermediate Peptide Purification

Crude peptides were purified by semi-preparative RP-HPLC using a Vydac 219TP 5 µm biphenyl column with a flow rate of 5 mL/min. Detection was done at 220, 254 and 280 nm. Purification was conducted with aqueous 0.1% TFA (solvent A) and 0.1% TFA in acetonitrile (solvent B) as eluents. Gradient began with 15% MeCN/85% H_2O (0.1% TFA) for the first 5 min. Gradient ramped up to 33% MeCN over 30 min, then final ramp up to 100% MeCN over the next 1 min and held for 5 min.

Fractions containing the peptide, as checked by MALDI, were pooled, lyophilized, and re-dissolved in MeOH.

Semi-pure peptides were re-injected and purified by analytical RP-HPLC using a ZORBAX 300SB-C3 column with a flow rate of 1 mL/min. Gradient was ramped up from 15% MeCN/85% H_2O (0.1% TFA) to 25% MeCN/75% H_2O (0.1% TFA) for the first 2 min. Gradient ramped up to 35% MeCN over 18 min, then final ramp up to 100% MeCN over the next 1 min and held for 5 min.

1.5.4 Purification of Partially Reduced Nem/Nmm Alkylated Peptide

Crude peptides were purified by analytical RP-HPLC using a ZORBAX 300SB-C3 column with a flow rate of 1 mL/min. Detection was done at 220, 254 and 280 nm. Purification was conducted with aqueous 0.1% TFA (solvent A) and 0.1% TFA in acetonitrile (solvent B) as eluents. Gradient began with 20% MeCN/80% H_2O (0.1% TFA) for the first 2 min. Gradient ramped up to 70% MeCN over 15 min, then final ramp up to 100% MeCN over the next 1 min and held for 5 min. Fractions containing the peptide, as checked by LCMS.

2) Synthesis of enantiopure thiazolidine derivative 47 [3]

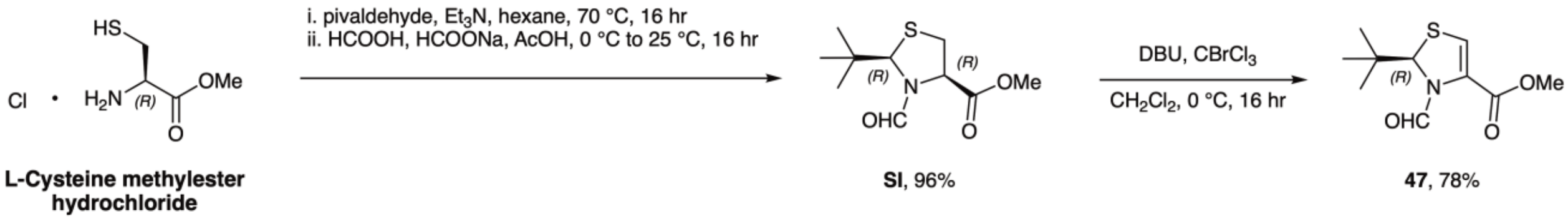

Scheme S1. Synthesis of thiazoline derivative **47**

2.1 Methyl (2*R*,4*R*)-2-(*tert*-butyl)-3-formylthiazolidine-4-carboxylate (S1)

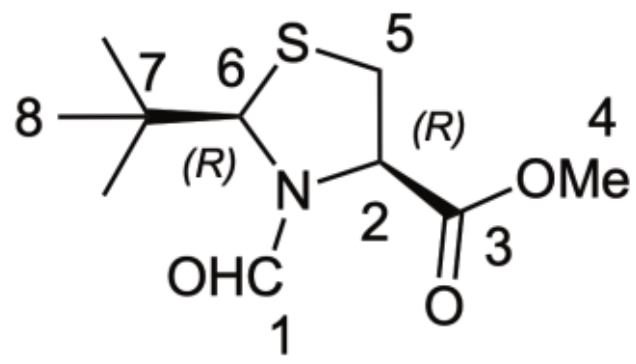

S1

The following was prepared according to a literature procedure.[3] Triethylamine (0.64 mL, 4.57 mmol, 1.1 equiv) was added to a solution of L-cysteine methyl ester hydrochloride (0.71 g, 4.15 mmol, 1.0 equiv) and pivaldehyde (0.50 mL, 4.57 mmol, 1.1 equiv) in hexanes (5 mL) and heated to reflux with Dean-Stark trap. After 16 h, the reaction was cooled to room temperature and the solids were removed by gravity filtration. The solvent was evaporated under reduced pressure and the resulting yellow oil was cooled to 0 °C. Formic acid (10 mL) was added followed by sodium formate (0.34 g, 5.00 mmol, 1.2 equiv). After 10 mins at 0 °C, acetic anhydride (1.18 mL, 12.5 mmol, 3.0 equiv) was added dropwise over 5 mins and the solution was allowed to warm up to room temperature. After 16 h, the solvent was removed under reduced pressure, and then the residual acid was quenched by the addition of sat. $NaHCO_{3(aq)}$. The solution was extracted with EtOAc (3 × 10 mL). The organic layers were combined, dried over Na_2SO_4, filtered and concentrated *in vacuo*. The resulting oil was purified by column chromatography (10-20% EtOAc/hexanes) to give the pure product **S1** as a colorless oil (0.92 g, 96%); R_f = 0.20 (20% EtOAc/hexanes).

^{1}H NMR (600 MHz, $CDCl_3$) δ 8.37 (1 H, s, H1), 4.91 (1 H, app t, J = 9.0 Hz, H2), 4.77 (1 H, s, H6), 3.79 (3 H, s, H4), 3.33 (1 H, dd, J = 12.0, 9.0 Hz, H5), 3.29 (1 H, dd, J = 12.0, 9.0 Hz, H5), 1.06 (9 H, s, H8).

^{13}C NMR (150 MHz, $CDCl_3$) δ 170.0 (C3), 162.6 (C1), 75.1(C6), 61.4 (C2), 52.6 (C4), 38.5 (C7), 32.8 (52), 26.3 (C8).

IR (cast film, ν_{max} / cm^{-1}) 2958, 2917, 2872, 1753, 1678, 1484, 1437, 1395, 1365, 1197, 1177.

OR: $[\alpha]_D^{26}$ = –117.07 (c = 0.300, DCM)

HRMS (ESI–TOF) $[M+Na]^+$ calc'd for $C_{10}H_{17}NNaO_3S$ 254.0929, found 254.0821.

Data in accordance with literature.[3]

2.2 Methyl (2*R*,4*R*,5*R*)-5-(4-(*tert*-butoxy)phenyl)-2-(*tert*-butyl)-3-formylthiazolidine-4-carboxylate (47)

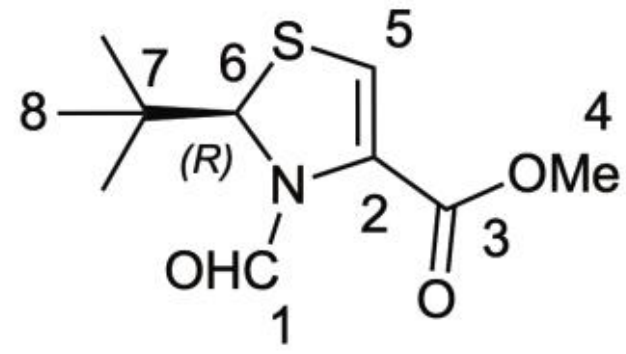

47

The following was prepared according to a literature procedure.[3] DBU (0.71 g, 4.67 mmol, 1.8 equiv) was added to a solution of saturated ester **S1** (0.60 g, 2.60 mmol, 1.0 equiv) in dichloromethane (6.0 mL) at 0 °C. Bromotrichloromethane (0.33 mL, 3.37 mmol, 1.3 equiv) was added and the solution stirred at 0 °C for 16 hours. After this time the reaction was quenched by addition of sat. $NaHCO_{3(aq)}$ (6.0 mL) and then warmed to room temperature. The solution was extracted with CH_2Cl_2 (3 × 10 mL). The organic layers were combined, dried over Na_2SO_4, filtered and concentrated *in vacuo*. The resulting oil was purified by column chromatography (20% EtOAc/hexanes) to give the pure product **47** as a colorless oil (0.46 g, 78%); R_f = 0.30 (20% EtOAc/hexanes).

^{1}H NMR (600 MHz, $CDCl_3$) δ 8.87 (1 H, s, H1), 7.08 (1 H, s, H5), 5.85 (1 H, s, H6), 3.84 (3 H, s, H4), 0.97 (9 H, s, H8).

^{13}C NMR (150 MHz, $CDCl_3$) δ 164.0 (C1), 159.3 (C3), 128.7 (C2) 127.6 (C5), 74.4 (C6), 52.5 (C4), 39.3 (C7), 24.6 (C8).

IR (cast film, ν_{max} / cm^{-1}) 3098, 2967, 2907, 2870, 1717, 1684, 1578, 1477, 1439, 1328, 1289, 1243.

OR: $[\alpha]_D^{26}$ = +170.52 (c = 0.850, DCM)

HRMS (ESI–TOF) $[M+Na]^+$ calc'd for $C_{10}H_{15}NNaO_3S$ 252.0665, found 252.0662.

Data in accordance with literature.[3]

3) Synthesis of β-Thiol Aspartic acid from Thiazoline Scaffold

3.1 Dimethyl 2-((2*R*,4*R*,5*R*)-2-(*tert*-butyl)-3-formyl-4-(methoxycarbonyl)thiazolidin-5-yl)malonate (52)

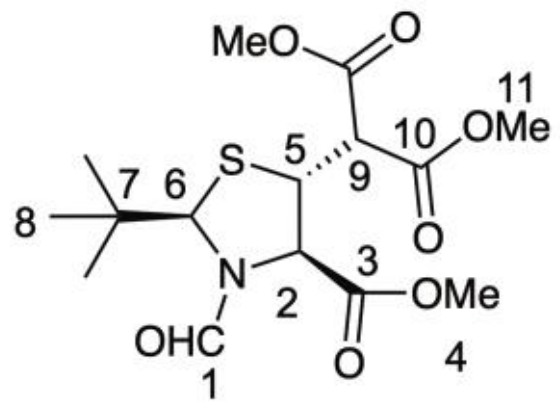

52

A 60% dispersion of sodium hydride in mineral oil (192 mg, 4.80 mmol) was added in anhydrous THF (12.0 mL) at 0 °C. Dimethyl malonate (0.997 mL, 8.72 mmol) was added dropwise followed by thiazoline analog **47** (1.00 g, 4.36 mmol) in anhydrous THF (4.00 mL) dropwise. The reaction mixture was allowed to warm to room temperature. After 16 hours, the solution was quenched by saturated aqueous NH_4Cl then extracted with ethyl acetate (3 × 20 mL). The combined organic extracts were dried over Na_2SO_4, filtered and concentrated *in vacuo* to give a yellow liquid. The crude mixture was purified *via* flash column chromatography (20–30% EtOAc/hexanes) to yield the pure product **52** (1.54 g, 98%) as a colorless oil (1:0.42 mixture of rotamers, data given for major rotamer); R_f = 0.20 (30% EtOAc/hexanes).

High temperature ^{1}H NMR (400 MHz, DMSO-d_6) spectra were provided in **Section 14**.

^{1}H NMR (500 MHz, $CDCl_3$) δ 8.34 (1 H, s, H1), 5.36 (1 H, d, J = 3.0 Hz, H2), 4.87 (1 H, s, H6), 4.57 (1 H, dd, J = 3.0, 8.5 Hz, H5), 3.76 (6 H, s, H11), 3.75 (3 H, s, H4), 3.57 (1 H, d, J = 8.5 Hz, H9), 0.96 (9 H, s, H8).

^{13}C NMR (125 MHz, $CDCl_3$) δ 169.2 (C3), 167.3 (C10), 167.1 (C10), 162.9 (C1), 75.2 (C6), 71.165 (C5), 63.1 (C2), 55.9 (C9), 53.1 (C4), 53.0 (C11), 52.9 (C1), 46.2 (C), 37.4 (C7), 26.2 (C8).

IR (cast film, ν_{max} / cm^{-1}) 2957, 1741, 1684, 1482, 1426, 1390, 1367, 1317, 1253, 1179.

OR: $[\alpha]_D^{26}$ = +14.12 (c = 0.330, DCM)

HRMS (ESI–TOF) $[M+Na]^+$ calc'd for $C_{15}H_{23}NNaO_7S$ 384.1087, found 384.1085.

3.2 (4*R*,5*R*)-5-(2-(Allyloxy)-2-oxoethyl)-2-(*tert*-butyl)thiazolidine-4-carboxylic acid (53)

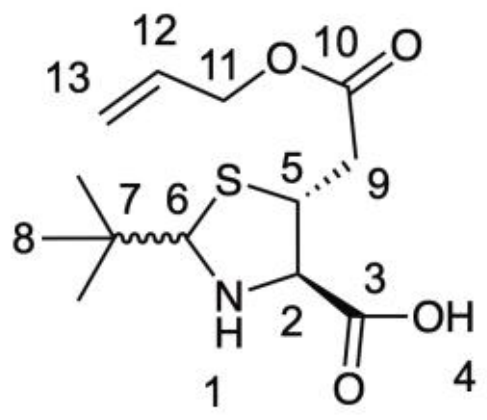

53

Compound **52** (100 mg, 0.277 mmol) was dissolved in 1,4-dioxane (2.0 mL) followed by 6 N aq. HCl (2.00 mL). The reaction mixture was heated with an oil bath at 100 °C. After 16 hours, the solvent was removed under reduced pressure to give a yellow oil which was used in the next step without purification. The crude product was re-dissolved with allyl alcohol (1.39 mL). Next, chlorotrimethylsilane (0.11 mL, 0.886 mmol) was added dropwise *via* syringe. The reaction mixture was allowed to stir at room temperature for 3 hours then quenched with H_2O and extracted with Et_2O (3 × 10 mL). The organic layer was dried over Na_2SO_4, filtered and concentrated *in vacuo* to afford a yellow oil. The crude product was purified *via* flash column chromatography (30% acetone/hexanes + 1% AcOH) to give the pure product **53** (38.2 mg, 48% in two steps) as a yellow oil; R_f = 0.20 (30% acetone/hexanes + 1% AcOH).

^{1}H NMR (500 MHz, $CDCl_3$) δ 5.99 – 5.91 (1 H, m, H12), 5.37 (1 H, app d, J = 17.0 Hz, *trans* H13) 5.30 – 5.27 (1 H, m, *cis* H13), 4.65 – 4.60 (2 H, m, H11), 4.65 (1 H, m, H6), 4.30 – 4.21 (1 H, m, H5), 3.89 – 3.79 (1 H, m, H2), 3.21 – 3.18 (1 H, m, H4), 2.83 – 2.77 (2 H, m, H9), 1.12 (9 H, s, H8).

^{13}C NMR (125 MHz, CD_3OD) δ 172.3 (C3), 171.0 (C10), 131.9 (C12), 118.6 (C13), 77.4 (C6), 68.9 (C2), 65.7 (C11), 48.3(C5), 40.3 (C9), 34.4 (C7), 26.0 (C8)

IR (cast film, ν_{max} / cm^{-1}) 3084, 2960, 2876, 1739, 1643, 1550, 1476, 1373, 1296, 1237, 1221, 1177.

HRMS (ESI–TOF) $[M–H]^–$ calc'd for $C_{13}H_{20}NO_4S$ 286.1119, found 286.1118.

3.3 (2*R*,3*R*)-2-((((9*H*-Fluoren-9-yl)methoxy)carbonyl)amino)-5-(allyloxy)-3-(methyldisulfaneyl)-5-oxopentanoic acid (54)

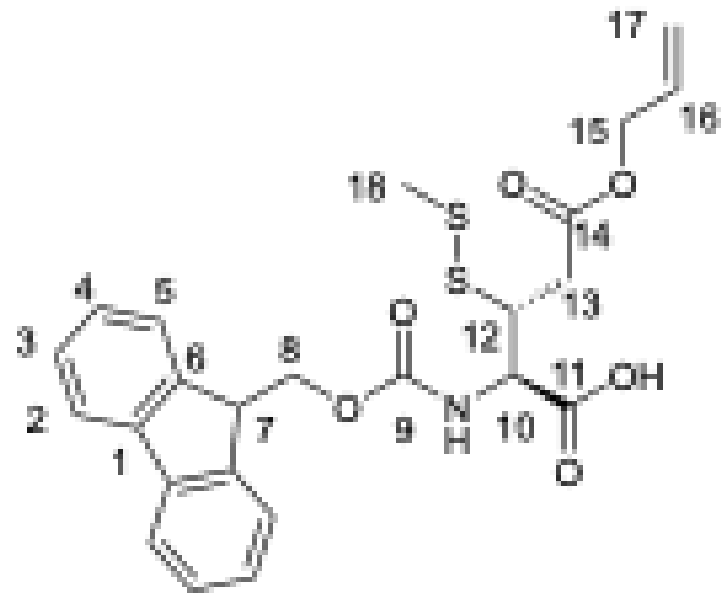

54

Compound **53** (80 mg, 0.278 mmol) was dissolved in MeOH (1.39 mL) and CH_2Cl_2 (1.39 mL). *S*-methyl methanethiosulfonate (60.4 µL, 0.639 mmol) was added dropwise *via* syringe followed by triethylamine (58.1 µL, 0.417 mmol). The reaction mixture was allowed to stir at 60 °C for 16 hours. After that time, the solvent was removed under reduced pressure and then re-dissolved in H_2O (0.695 mL). Fmoc *N*-hydroxysuccinimide ester (93.8 mg, 0.278 mmol) in acetonitrile (0.695 mL) was added as a solution, followed by triethylamine (77.5 µL, 0.556 mmol). The reaction mixture was allowed to stir at room temperature. After 5 hours, the reaction solution was quenched with 1 M HCl then extracted with ethyl acetate (3 × 10 mL). The organic layer was dried over Na_2SO_4, filtered and concentrated *in vacuo* to afford a brown oil. The crude product was purified *via* flash column chromatography (20% EtOAc/toluene + 2% AcOH) to give the pure product **54** (70.0 mg, 52% in two steps) as a white solid; R_f = 0.20 (30% EtOAc/hexanes).

^{1}H NMR (500 MHz, $CDCl_3$) δ 7.80 (2 H, d, *J* = 7.0 Hz, Ar-H), 7.63 – 7.60 (2 H, m, Ar-H), 7.42–7.37 (2 H, m, Ar-H), 7.33 – 7.30 (2 H, m, Ar-H), 5.95 – 5.90 (1 H, m, C16), 5.64 (1 H, d, *J* = 9.0 Hz, NH), 5.34 (1 H, d, *J* = 17.0 Hz, *trans* H17), 5.25 (1 H, d, *J* = 10.5 Hz, *cis* H17), 4.89 (1 H, app d, *J* = 6.5 Hz, H10), 4.61 – 4.64 (2 H, m, H15), 4.44 – 4.43 (2 H, m, H8), 4.24 (1 H, app t, *J* = 7.2 Hz, H7), 3.96 – 3.94 (1 H, m, H12), 2.91 – 2.77 (2 H, m, H13), 2.42 (3 H, s, H18).

^{13}C NMR (176 MHz, $CDCl_3$) δ 174.2 (C11), 170.2 (C14), 156.4 (C9), 143.8 (Ar-C), 141.5 (Ar-C), 141.4 (Ar-C), 131.8 (C16), 127.8 (Ar-C), 127.2 (Ar-C), 125.1 (Ar-C), 120.1 (Ar-C), 118.8 (C17), 67.5 (C8), 65.9 (C15), 56.6 (C10), 48.6 (C12), 47.1 (C7), 36.9 (C13), 23.0 (C18).

IR (cast film, ν_{max} / cm^{-1}) 3324, 3069, 2954, 1732, 1516, 1478, 1450, 1249, 1232.

OR: $[\alpha]_D^{26}$ = +25.2 (c = 0.150, DCM)

HRMS (ESI–TOF) $[M–H]^-$ calc'd for $C_{24}H_{24}NO_6S_2$ 486.1051, found 486.1036.

4) Synthesis of β-Thiol Tyrosine from Thiazoline Scaffold

4.1 Methyl (2*R*,4*R*,5*R*)-5-(4-(*tert*-Butoxy)phenyl)-2-(*tert*-butyl)-3-formylthiazolidine-4-carboxylate (55)

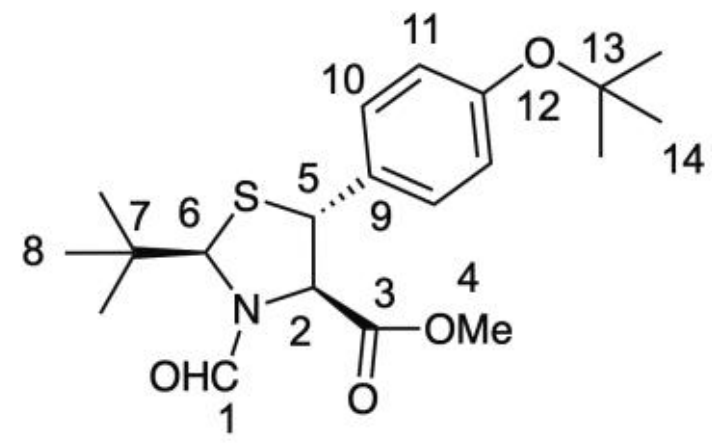

55

A solution of Grignard reagent was prepared as follows; 4-*tert*-butoxyphenyl bromide (220 mg, 0.96 mmol) was added to a suspension of magnesium (27 mg, 1.10 mmol) in anhydrous THF (0.5 mL). A single crystal of iodine was added, and the solution heated to ca. 50 °C until the colour had changed from orange/brown to cloudy, and most of the magnesium had reacted (ca. 2.5 h). The Grignard solution was cooled to 0 °C and was added to a solution of copper bromide dimethyl sulfide (90 mg, 0.44 mmol) in anhydrous THF (1.0 mL) at –70 °C. The solution was stirred at –70 °C for 10 mins before warming to 0 °C for 40 mins and then cooling back to –40 °C.

A solution of unsaturated ester **47** (50 mg, 0.22 mmol) in anhydrous THF (0.60 mL) was added dropwise and the solution changed from pale yellow to deep red in colour. After 40 mins, the reaction was complete by TLC (20% EtOAc/hexanes) and was quenched with sat. $NH_4Cl_{(aq)}$ solution (5 mL) then extracted with CH_2Cl_2 (3 × 10 mL). The combined organic layers were dried ($MgSO_4$), filtered, and the solvent removed under reduced pressure. The resulting oil was purified by column chromatography (5-20% EtOAc/hexanes) to give the pure product **55** (40 mg, 60%) as a colourless oil (1:0.15 mixture of rotamers, data given for major rotamer).

^{1}H NMR (500 MHz, $CDCl_3$) δ 8.38 (1 H, s, H1), 7.28 (2 H, d, *J* = 8.6 Hz, Ar-H), 6.94 (2 H, d, *J* = 8.6 Hz, Ar-H), 4.90 (1 H, s, H6), 4.85 (1 H, d, *J* = 8.8 Hz, H5), 4.81 (1 H, d, *J* = 8.8 Hz, H2), 3.66 (3 H, s, H4), 1.34 (9 H, s, H14), 1.11 (9 H, s, H8)

^{13}C NMR (125 MHz, $CDCl_3$) δ 170.0 (C3), 162.6 (C1), 155.8 (C12), 131.1 (Ar-C), 128.5 (Ar-C), 124.2 (Ar-C), 78.8 (C13), 75.7 (C6), 68.6 (C2), 52.7 (C5), 52.5 (C4), 38.7 (C7), 28.9 (C14), 26.5 (C8)

IR (cast film, ν_{max} / cm^{-1}) 2976, 2871, 1743, 1680, 1606, 1507, 1366, 1239, 1164.

OR: $[\alpha]_D^{26}$ = +28.39 (c = 0.25, Chloroform)

HRMS (ESI–TOF) $[M+H]^+$ calc'd for $C_{20}H_{30}NO_4S$ 380.1890, found 380.1893.

4.2 (4*R*,5*R*)-5-(4-(*tert*-Butoxy)phenyl)-2-(*tert*-butyl)thiazolidine-4-carboxylic acid (56)

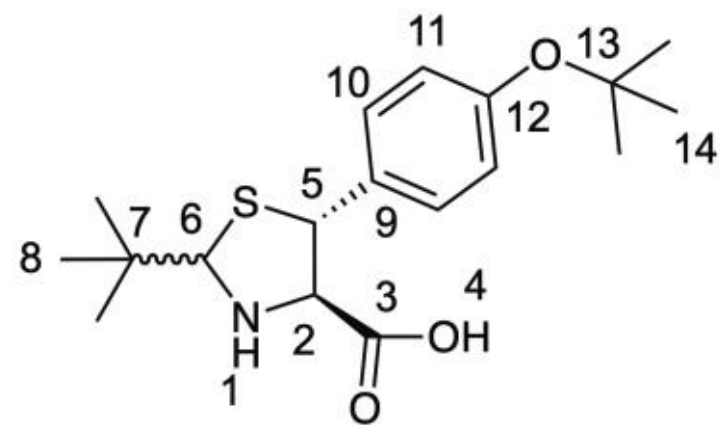

56

55 (28 mg, 0.073 mmol) was dissolved in THF (1.3 mL) and cooled to 0 °C. Sodium hydroxide (aq. 0.4 M, 0.55 mL) was added and the solution stirred at 0 °C for 1 h before warming to room temperature. After a further 2 h, the solvent was evaporated under reduced pressure and hydrochloric acid (1 M, 1 mL) was added. The solution was extracted with ethyl acetate (3 × 5 mL) and the combined organic layers was dried over magnesium sulfate, filtered, and the solvent removed under reduced pressure to give **23** (26.2 mg, 98%) as a yellow solid which was carried through to the next step without further purification

Intermediate acid (150 mg, 0.41 mmol) was dissolved in anhydrous THF and cooled to 0 °C. Ethyl magnesium bromide (0.54 mL, 1.64 mmol) was added dropwise over 5 mins and the solution was allowed to warm to room temperature. After 5 h, the reaction was quenched by the addition of water (4 drops) and then the solvent was removed under reduced pressure. The resulting residue was purified by column chromatography (10% methanol/dichloromethane) to give the pure product **56** (0.15 g, quant.) as a colourless solid.

^{1}H NMR (400 MHz, CD_3OD) δ 7.33 (2 H, d, J = 8.5 Hz, Ar-H), 6.92 – 6.87 (2 H, m, Ar-H), 4.79 (1 H, s, H6), 4.40 (1 H, d, J = 8.8 Hz, H5), 3.62 (1 H, d, J = 8.8 Hz, H2), 1.31 (9 H, s, H14), 1.09 (9 H, s, H8).

^{13}C NMR (100 MHz, CD_3OD) δ 176.3 (C3), 154.3 (Ar-C), 135.5 (Ar-C), 128.4 (Ar-C), 123.6 (Ar-C), 80.8 (C6), 78.2 (C13), 76.1 (C2), 58.8 (C5), 33.7 (C7), 27.8 (C14), 26.0 (C8)

IR (cast film, ν_{max} / cm^{-1}) 2974, 2937, 2914, 2872, 1724, 1607, 1506, 1366, 1162.

HRMS (ESI–TOF) $[M+H]^+$ calc'd for $C_{18}H_{26}NO_3S$ 336.1639, found 336.1628.

4.3 (2*R*,3*R*)-2-((((9*H*-Fluoren-9-yl)methoxy)carbonyl)amino)-3-(4-(*tert*-butoxy)phenyl)-3-(methyldisulfaneyl)propanoic acid (57)

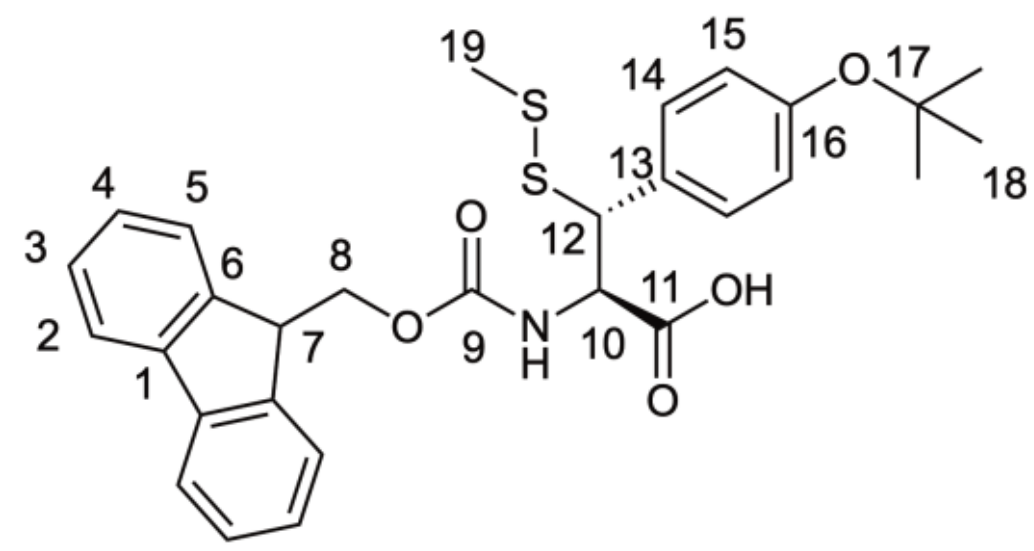

57

S-Methyl methanethiosulfonate (64 µL, 0.68 mmol) and triethylamine (62 µL, 0.44 mmol) were added to **56** in methanol (2.5 mL) and dichloromethane (2.5 mL) at room temperature. After 22 h, the solvent was evaporated, and the residue was re-dissolved in water (0.7 mL). Triethylamine (87 µL, 0.62 mmol) was added, followed by a solution of Fmoc-succinimide (110 mg, 0.33 mmol) in acetonitrile (0.7 mL). The reaction was stirred at room temperature for 1 h and 45 mins before quenching with aqueous hydrochloric acid (1 M, 2 mL), and then extracting with ethyl acetate (3 × 2 mL). The combined organic layers were dried (Na_2SO_4), filtered, and the solvent evaporated to give a yellow residue. The product was purified by column chromatography (30% EtOAc/hexanes + 0.5% AcOH) to give the product **57** (68 mg, 43%) as a colourless oil.

^{1}H NMR (700 MHz, $CDCl_3$) δ 7.80 (2 H, d, *J* = 7.6 Hz, Ar-H), 7.64 (2 H, t, *J* = 6.7 Hz, Ar-H), 7.44 (2 H, t, *J* = 7.5 Hz, Ar-H), 7.35 (2 H, t, *J* = 7.4 Hz, Ar-H), 7.30 – 7.25 (2 H, m, Ar-H), 6.99–6.93 (2 H, m, Ar-H), 5.70 (1 H, d, *J* = 8.8 Hz, NH), 4.95 (1 H, t, *J* = 7.3 Hz, H10), 4.56 (1 H, d, *J* = 5.8 Hz, H12), 4.50 – 4.41 (2 H, m, H8), 4.28 (1 H, t, *J* = 7.2 Hz, H7), 2.18 (3 H, s, H19), 1.35 (9 H, s, 9H, H18).

^{13}C NMR (176 MHz, $CDCl_3$) δ 172.3 (C11), 156.0 (C9), 155.7 (Ar-C), 143.6 (Ar-C), 141.3 (Ar-C), 131.7 (Ar-C), 129.0 (Ar-C), 127.8 (Ar-C), 127.1 (Ar-C), 125.1 (Ar-C), 124.0 (Ar-C), 120.0 (Ar-C), 78.8 (C17), 67.5 (C8), 57.3 (C10), 55.6 (C12), 47.1 (C7), 28.8 (C19), 23.0 (C18)

IR (cast film, ν_{max} / cm^{-1}) 3065, 3042, 2974, 2957, 2925, 2874, 2856, 1720, 1505, 1236, 1160, 1049.

OR: $[\alpha]_D^{26}$ = –21.94 (c = 0.310, Chloroform)

HRMS (ESI–TOF) $[M–H]^-$ calc'd for $C_{29}H_{30}NO_5S_2$ 536.1571, found 536.1567.

5) Synthesis of β-Thiol Phenylalanine from Thiazoline Scaffold

5.1 Methyl (2*R*,4*R*,5*R*)-2-(*tert*-butyl)-3-formyl-5-phenylthiazolidine-4-carboxylate (58)

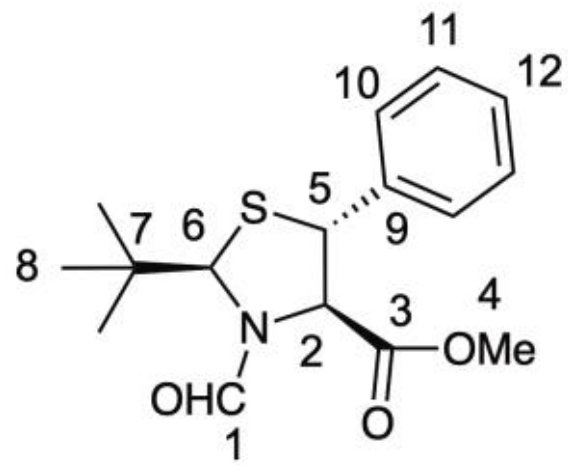

58

Grignard reagent was prepared by adding bromobenzene (1.0 mL, 9.60 mmol) to a suspension of magnesium (0.27 g, 10.9 mmol) in anhydrous THF (15 mL) at room temperature. A single crystal of iodine was added to aid activation and the solution decolourised after around 3 minutes. The solution was left for 1.5 h at room temperature before cooling to –78 °C. Copper bromide dimethylsulfide complex (0.90 g, 4.36 mmol) was added in one portion and the suspension was stirred at –78 °C for 20 mins. The reaction was then warmed to 0 °C for 30 mins before cooling to –50 °C. A solution of **47** (0.50 g, 2.18 mmol) in anhydrous THF (3 mL) was then added dropwise causing the solution to turn from white to bright yellow. After 45 minutes the reaction was complete by TLC and quenched with saturated aqueous ammonium chloride (10 mL) and then warmed to room temperature. The solution was extracted with ethyl acetate (3 × 20 mL) and the combined organic layers was dried over magnesium sulfate, filtered, and the solvent was removed under reduced pressure. The crude product was purified by column chromatography (20% ethyl acetate/hexanes) to give the pure product **58** (0.34 g, 50%) as a yellow oil which solidified on standing; (5:1 mixture of rotamers, data given for major rotamer)

^{1}H NMR (400 MHz, $CDCl_3$) δ 8.40 (1 H, s, H1), 7.43 – 7.27 (5 H, m, Ar-H), 4.93 (1 H, s, H6), 4.90 (1 H, d, J = 8.9 Hz, H2/H5), 4.88 (1 H, d, J = 8.9 Hz, H2/H5), 3.68 (3 H, s, H4), 1.12 (9 H, s, H8).

^{13}C NMR (101 MHz, $CDCl_3$) δ 169.5 (C3), 162.2 (C1), 136.4 (Ar-C), 128.5 (Ar-C), 128.2 (Ar-C), 127.5 (Ar-C), 75.4 (C6), 68.0 (C2/C5), 52.7 (C4), 52.1 (C2/C5), 38.3 (C7), 26.0 (C8).

IR (cast film, ν_{max} / cm^{-1}) 3031, 2958, 2910, 2874, 1751, 1677, 1395, 1364, 1310, 1201, 1175, 1015, 766, 699.

HRMS (ESI–TOF) $[M+Na]^+$ calc'd for $C_{16}H_{21}NNaO_3S$ 330.1134, found 330.11334.

5.2 (4*R*,5*R*)-2-(*tert*-Butyl)-5-phenylthiazolidine-4-carboxylic acid (59)

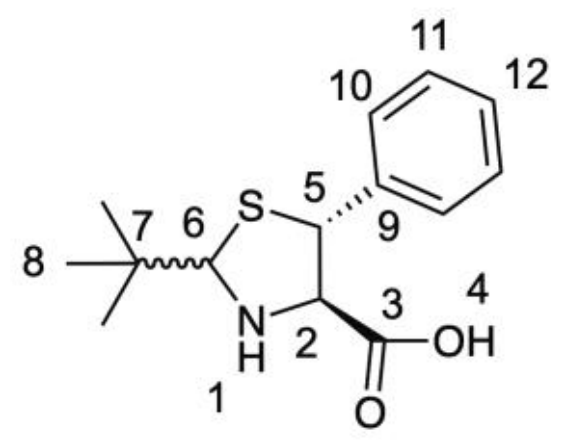

59

Methyl ester **58** (300 mg, 0.976 mmol) was dissolved in 1,4-dioxane (1 mL) and aqueous hydrochloric acid (6 M, 1 mL) was added and the solution heated to 100 °C for 16 h. After this time the product was purified by column chromatography (20% acetone/toluene + 1% AcOH) to give **59** (170 mg, 65%) as a colourless solid; (2:1 mixture of rotamers - data given for major rotamer)

^{1}H NMR (400 MHz, CD_3OD) δ 7.52 – 7.35 (2 H, m, Ar-H), 7.35 – 7.19 (3 H, m, Ar-H), 4.84 (1 H, d, J = 2.3 Hz, H6), 4.43 (1 H, d, J = 8.5 Hz, H5), 3.80 (1 H, d, J = 8.5 Hz, H2), 1.10 (9 H, d, J = 2.1 Hz, H8).

^{13}C NMR (101 MHz, CD_3OD) δ 173.1 (C3), 140.8 (Ar-C), 129.1 (Ar-C), 128.7 (Ar-C), 128.2 (Ar-C), 81.9 (C6), 74.4 (C2), 59.7 (C5), 34.8 (C7), 26.9 (C8).

IR (cast film, ν_{max} / cm^{-1}) 3276, 2959, 2870, 2469 (br), 1717, 1472, 1368, 1273, 1075, 931, 788, 698.

HRMS (ESI–TOF) $[M–H]^-$ calc'd for $C_{14}H_{18}N_2O_2S$ 264.1064, found 264.1065.

5.3 (2*R*,3*R*)-2-((((9*H*-Fluoren-9-yl)methoxy)carbonyl)amino)-3-(((4-methoxyphenyl)-diphenylmethyl)thio)-3-phenylpropanoic acid (60)

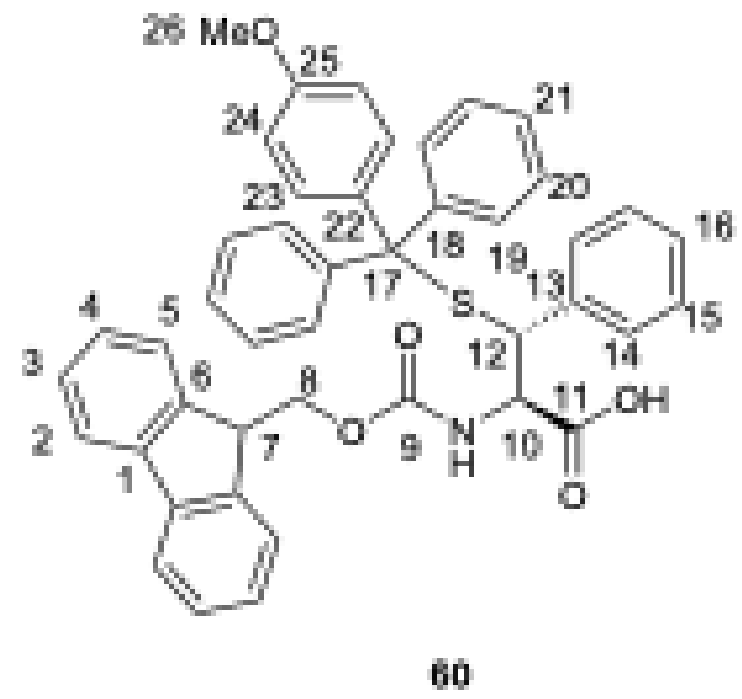

60

Acid **59** (30 mg, 0.113 mmol) was suspended in dichloromethane (1.5 mL) and triethylamine (33 µL, 0.232 mmol) was added causing dissolution. Monomethoxytrityl chloride (80 mg, 0.260 mmol) was added and the solution was stirred at room temperature for 3 h. The solvent was then evaporated under reduced pressure and the resulting residue was dried under high vacuum. Water (0.6 mL) was added

followed by triethylamine (33 μL, 0.232 mmol) and then a solution of Fmoc succinimide (42 mg, 0.124 mmol) in acetonitrile (0.6 mL). The solution was stirred for 16 h at room temperature before addition of hydrochloric acid (aq. 1.0 M, 1 mL). The solution was extracted with ethyl acetate (3 × 5 mL) and the combined organics dried over magnesium sulfate, filtered, and the solvent removed under reduced pressure. The product was purified by column chromatography (5% ethyl acetate + 1% acetic acid/hexanes) to give the product **60** (15 mg, 19%) as a bright yellow oil.

^{1}H NMR (500 MHz, $CDCl_3$) δ 7.78 (2 H, dd, J = 13.4, 7.6 Hz, Ar-H), 7.57 (2 H, t, J = 8.5 Hz, Ar-H), 7.48 – 7.25 (9 H, m, Ar-H), 7.24 – 7.13 (10 H, m, Ar-H), 7.01 (2 H, d, J = 7.4 Hz, Ar-H), 6.66 (2 H, d, J = 8.9 Hz, Ar-H), 4.95 (1 H, d, J = 8.8 Hz, NH), 4.57 (1 H, t, J = 7.4 Hz, H12), 4.47 (1 H, t, J = 8.8 Hz, H8), 4.38 – 4.30 (1 H, m, H8), 4.20 (1 H, t, J = 6.9 Hz, H7), 3.74 (3 H, s, H26), 3.74 – 3.72 (1 H, m, H10);

^{13}C NMR (125 MHz, $CDCl_3$) δ 173.7 (C11), 158.2 (C25), 155.5 (C9), 144.4 (Ar-C), 144.3 (Ar-C), 143.8 (Ar-C), 143.7 (Ar-C), 141.4 (Ar-C), 141.3 (Ar-C), 139.1 (Ar-C), 135.9 (Ar-C), 131.1 (Ar-C), 129.7 (Ar-C), 129.6 (Ar-C), 128.7 (Ar-C), 128.2 (Ar-C), 127.9 (Ar-C), 127.8 (Ar-C), 127.1 (Ar-C), 127.1 (Ar-C), 126.8 (Ar-C), 126.8 (Ar-C), 125.1 (Ar-C), 125.0 (Ar-C), 120.0 (Ar-C), 120.0 (Ar-C), 113.0 (Ar-C), 69.2 (C17), 67.0 (C8), 59.2 (C12), 55.2 (C26), 51.2 (C10), 47.2 (C7).

IR (cast film, ν_{max} / cm^{-1}) 3401, 3057, 3029, 2954, 2936, 1720, 1508, 1450, 1251, 1181, 1034, 738, 699.

OR: $[\alpha]_D^{26}$ = –90.09 (c = 0.890, DCM)

HRMS (ESI–TOF) $[M–H]^-$ calc'd for $C_{44}H_{36}NO_5S$ 690.2320, found 690.2315.

5.4 (2*R*,3*R*)-2-((((9*H*-Fluoren-9-yl)methoxy)carbonyl)amino)-3-(methyldisulfaneyl)-3-phenyl-propanoic acid (61)

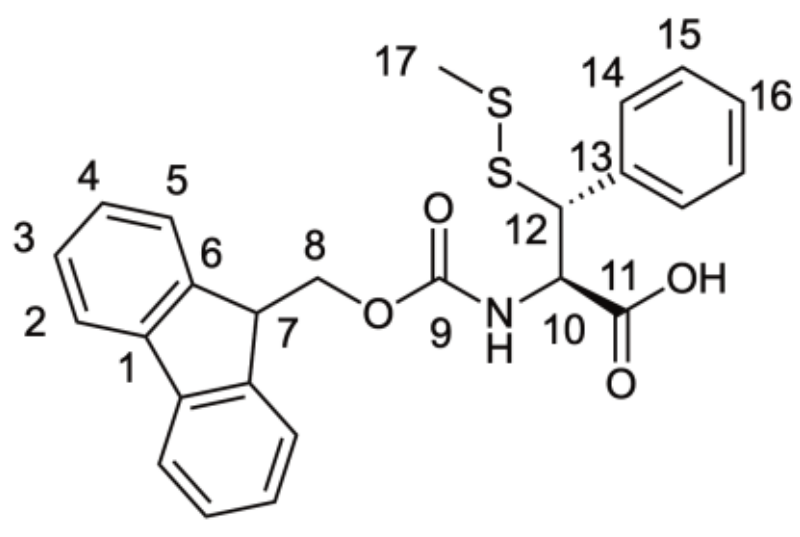

61

Acid **60** (1.17 g, 2.51 mmol) was dissolved in acetonitrile (23 mL) and a solution of TCEP hydrochloride (3.35 g, 11.7 mmol) in water (23 mL) was added, followed by saturated aqueous sodium hydrogen carbonate solution (4.1 mL). The solution was stirred at room temperature and after 4 h,

LCMS showed the reaction to be complete. Brine (40 mL) was added, then argon was bubbled through the solution for 20 mins to remove any residual methanethiol. The solution was then extracted with ethyl acetate (2 × 20 mL) then the combined organics dried (Na_2SO_4), filtered, and the solvent evaporated. The resulting residue was redissolved in DMF and Mmt-Cl (1.0 g, 3.26 mmol) was added and the solution was left to stir at room temperature. After 17 h, the reaction was partitioned between ethyl acetate and water and the organics extracted with ethyl acetate (3 × 20 mL). The combined organics were washed with water (2 × 50 mL), dried (Na_2SO_4), filtered, and the solvent evaporated. The product was purified by column chromatography (5-10% ethyl acetate/hexanes) to give the product **61** (1.01 g, 58%) as an off-white solid.

^{1}H NMR (500 MHz, $CDCl_3$) δ 7.83 – 7.76 (2 H, m, Ar-H), 7.68 – 7.60 (2 H, m, Ar-H), 7.48 – 7.41 (2 H, m, Ar-H), 7.41 – 7.30 (7 H, m, Ar-H), 5.71 (1 H, d, *J* = 9.0 Hz, NH), 4.99 (1 H, dd, *J* = 9.0, 5.5 Hz, H10), 4.58 (1 H, d, *J* = 5.6 Hz, H12), 4.52 – 4.41 (2 H, m, H8), 4.27 (1 H, t, *J* = 7.4 Hz, H7), 2.20 (3 H, s, H17).

^{13}C NMR (125 MHz, $CDCl_3$) δ 174.1 (C11), 156.0 (C9), 143.8 (Ar-C), 143.7 (Ar-C), 141.4 (Ar-C), 137.2 (Ar-C), 128.8 (Ar-C), 128.5 (Ar-C), 128.4 (Ar-C), 127.8 (Ar-C), 127.2 (Ar-C), 127.1 (Ar-C), 125.1 (Ar-C), 125.0 (Ar-C), 120.1 (Ar-C), 67.5 (C8), 57.2 (C10), 56.4 (C12), 47.1 (C7), 23.0 (C17).

IR (cast film, ν_{max} / cm^{-1}) 3302, 3066, 2918, 1720, 1514, 1451, 1265, 759, 740.

OR: $[\alpha]_D^{26}$ = –24.57 (c = 0.140, DCM)

HRMS (ESI–TOF) $[M–H]^-$ calc'd for $C_{25}H_{23}NO_4S_2$ 464.0996, found 464.0992.

6) Synthesis of MccJ25 analog from bis-disulfide/ β -thiol Glu strategy

6.1 Disulfide-containing MccJ25 analog with β-thiol-Glu (65)

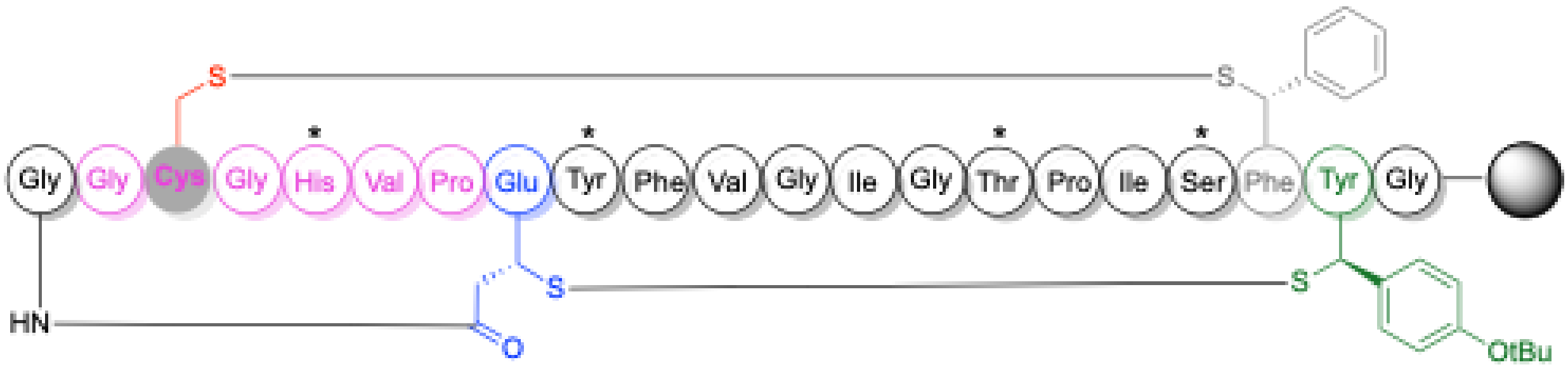

65

Synthetic MccJ25 analog **62** was synthesized according to the general SPPS protocol. Orthogonally protected β-thiol Tyr **57** was incorporated into position 20 by reduced loading procedure. Fmoc-β-thiol-Phe(Mmt)-OH **61** was (3 equiv) incorporated into position 19 using HATU (2.9 equiv), HOAt (2.9 equiv), and DIPEA (15 equiv), and allowed to bubble with argon for 3 hours. Orthogonally protected β-thiol Glu **54** (2 equiv) was incorporated into position 8 using DIC (2 equiv) and HOBt (2 equiv), and allowed to bubble with argon for 3 hours. Fmoc-Cys(Mmt)-OH was incorporated into position 3 according to the general SPPS protocol. After incorporation of 21 amino acids on the resin, bis-disulfide formation was performed to give peptide **64**. Allyl deprotection was performed, followed by Fmoc removal to obtain peptide for macrocyclization. Side chain to tail cyclization was performed according to general cyclization procedure to afford peptide **65**.

6.2 MccJ25 analog from bis-disulfide/ β-thiol-Glu (66)

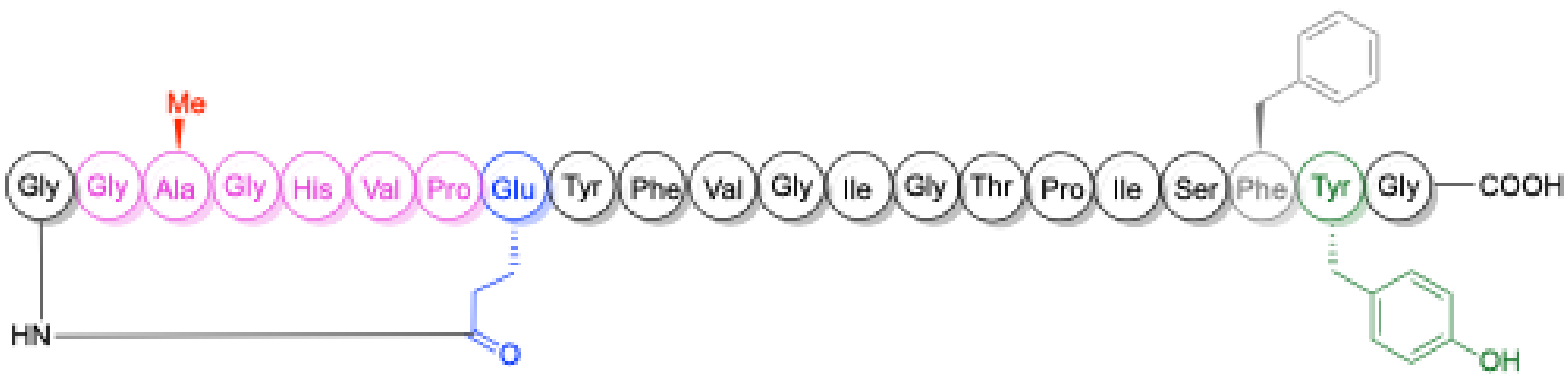

66

Bis-disulfide containing peptide **65** was cleaved off the resin and subjected to disulfide reduction and desulfurization according to protocol 1.4.8 to afford MccJ25 analog **66**.

7) Synthesis of γ-thiol Glu

7.1 Synthetic Scheme of γ-thiol Glu

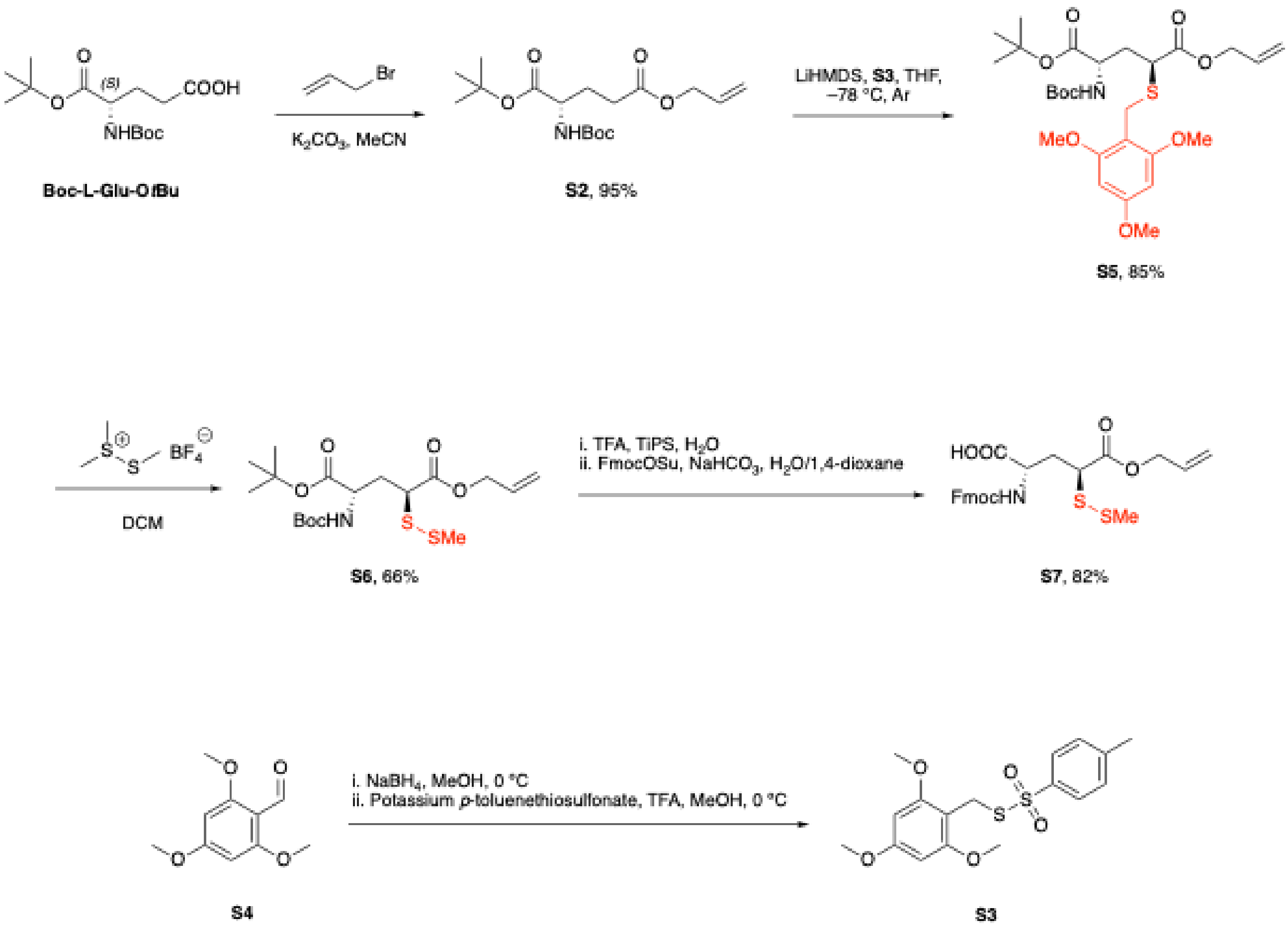

Scheme S2. Synthesis of γ-thiol Glu

The synthesis of the γ-thiol glutamic acid was done following the previously reported literature procedure.[4]

7.2 Boc-Glu(OAll)-O^{t}Bu (S2)

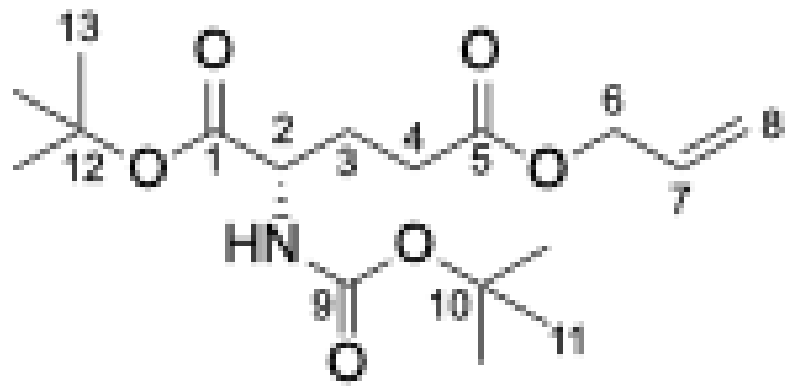

S2

To a solution of Boc-Glu-O^{t}Bu (2.4 g, 7.9 mmol) in acetonitrile (64 mL, 0.12 M), K_2CO_3 (2.0 g, 14.5 mmol) and allyl bromide (1.26 mL, 1.76 g, 14.5 mmol) were added portion wise and stirred overnight (appx. 16 h) at room temperature. Reaction was filtered through a short pad of Celite 545 and concentrated under reduced pressure. The crude residue was purified by column chromatography (10% EtOAc in hexanes) to furnish **S2** (2.6 g, 7.3 mmol, 95% yield) as a clear oil.

^{1}H NMR (700 MHz, $CDCl_3$) δ 5.91(ddt, 1H, J = 17.1 Hz, 10.5 Hz, 5.8 Hz, H-7), 5.31 (ddt, 1H, J = 17.1 Hz, 1.6 Hz, 1.6 Hz, H-8), 5.23 (ddt, 1H, J = 10.5 Hz, 1.5 Hz, 1.3 Hz, H-8), 5.06 (d, 1H, J = 8.0 Hz, *N*H), 4.58 (ddd, 2H, J = 5.9 Hz, 1.5 Hz, 1.3 Hz, H-6), 4.20 (dt, 1H, J = 8.0 Hz, 5.0 Hz, H-2), 2.45 (ddd, 1H, J = 16.6 Hz, 9.5 Hz, 6.5 Hz, H-4), 2.38 (ddd, 1H J = 16.4 Hz, 9.2 Hz, 6.2 Hz, H-4), 2.16 (m, 1H, H-3), 1.92 (m, 1H, H-3), 1.46 (s, 9H, H11/13), 1.43 (s, 9H, H11/13) ppm

^{13}C NMR (175 MHz, $CDCl_3$) δ 172.7, 171.4, 155.5 (C-9), 132.2 (C-7), 118.4 (C-8), 82.3 (C-10/12), 79.9 (C-10/12), 65.4 (C-6), 53.5 (C-2), 30.4 (C-4), 28.4 (C-11/13), 28.2 (C-11/13), 28.1 (C-3) ppm;

HRMS (ESI): calcd. for $C_{17}H_{29}NNaO_6$ 366.1887; found 366.1886.

Data agrees with the literature.[5]

7.3 *S*-(2,4,6-trimethoxybenzyl)toluenethiosulfonate (S3)

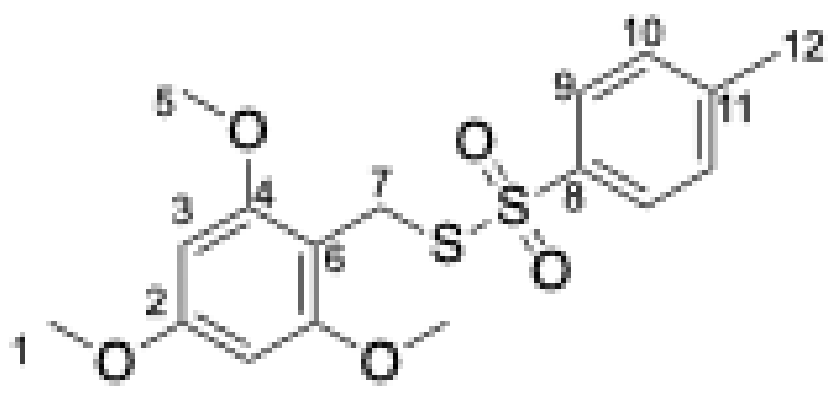

S3

To a solution of 2,4,6-trimethoxybenzaldehyde (0.5 g, 2.54 mmol) in MeOH (7 mL, 0.36 M) was added $NaBH_4$ (150 mg, 3.96 mmol) at 0 ºC. Reaction was stirred for 2 h at 0 ºC, diluted with Et_2O (20 mL), and washed with water and brine. The organic layer was dried over $MgSO_4$, filtered and concentrated under reduced pressure. The intermediate 2,4,6-trimethoxybenzyl alcohol was dissolved

in MeOH (7 mL, 0.36 M), and potassium *p*-toluenethiosulfonate (575 mg, 2.54 mmol), followed by TFA (0.2 mL, 0.3 g, 2.63 mmol), was added at 0 ºC. After 15 min, white precipitate started to form, which was separated by vacuum filtration. Crude crystalline product was purified by recrystallization from EtOAc to afford **S3** as white shiny crystals (580 mg, 1.57 mmol, 62% yield).

^{1}H NMR (700 MHz, $CDCl_3$) δ 7.85 (d, 2H, *J* = 8.4 Hz, H-9), 7.32 (d, 2H, *J* = 8.4 Hz, H-10), 6.01 (s, 2H, H-3), 4.29 (s, 2H, H-7), 3.77 (s, 3H, H-1), 3.69 (s, 6H, H-5), 2.45 (s, 3H, H-12) ppm

^{13}C NMR (175 MHz, $CDCl_3$): 161.4 (C-2), 159.1 (C-4), 143.8 (C-11), 142.4 (C-8), 129.4 (C-10), 127.1 (C-9), 102.5 (C-6), 90.3 (C-3), 55.6 (C-5), 55.3 (C-1), 29.0 (C-7), 21.5 (C-12) ppm

HRMS (ESI): calcd. for $C_{17}H_{20}NaO_5S_2$ 391.0644; found 391.0643.

IR (DCM cast film): 3049, 3000, 2970, 2944, 2887, 2839, 1594, 1499 cm^{-1}.

Data agrees with the literature.[4]

7.4 Boc-(4*S*-STmob)Glu(OAll)-O^{t}Bu (S5)

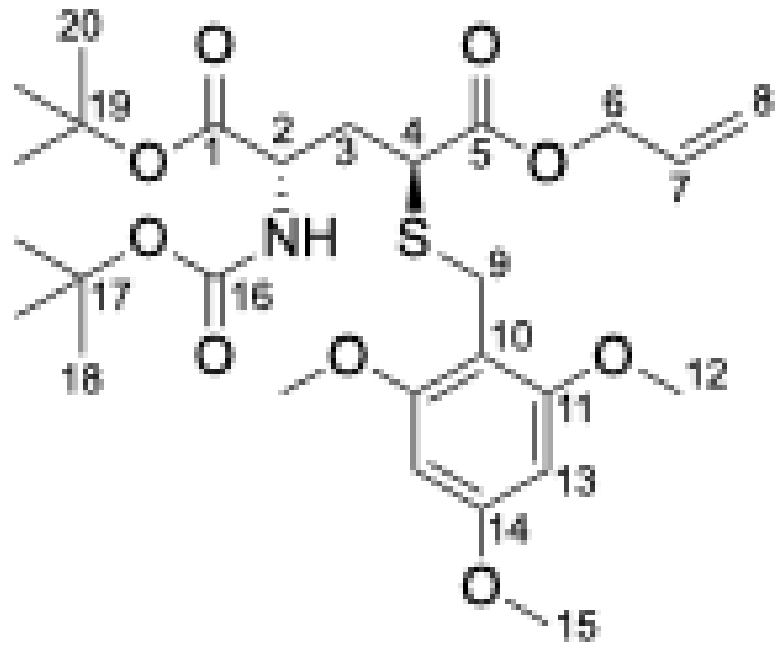

S5

To a solution of **S2** (0.97 g, 2.8 mmol) in THF (20 mL, 0.14 M) at -78 °C was added LiHMDS (1M in THF, 6.0 mL, 6.0 mmol) and stirred for 1 h at -78 °C. A solution of *S*-(2,4,6-trimethoxybenzyl)toluenethiosulfonate **S3** (1.2 g, 3.25 mmol) in THF (8 mL, 0.4 M) was then added dropwise over 10 min. After 2 h at -78 °C, the reaction was quenched with saturated aqueous NH_4Cl (20 mL) and the aqueous layer was extracted with DCM (3 × 20 mL). The organic layer was dried over anhydrous Na_2SO_4, filtered and concentrated under reduced pressure. The resulting residue was purified by column chromatography (5% EtOAc in hexanes) to afford **S5** as a clear oil (1.33 g, 2.4 mmol, 85% yield).

^{1}H NMR (700 MHz, $CDCl_3$) δ 6.10 (s, 2H, H-13), 5.93 (ddt, 1H, *J* = 17.0 Hz, 10.9 Hz, 5.8 Hz, H-7), 5.36 (ddt, 1H, *J* = 17.2 Hz, 1.6 Hz, 1.4 Hz, H-8), 5.23 (ddt, 1H, *J* = 10.5 Hz, 1.2 Hz, 1.2 Hz, H-8), 4.91 (d, 1H, *J* = 9.7 Hz, *N*H), 4.64 (ddt, 1H, *J* = 13.6 Hz, 5.6 Hz, 1.7 Hz, H-6), 4.58 (dd, 1H, *J* = 13.5 Hz, 5.6 Hz, H-6), 4.31 (m, 1H, H-2), 3.90 (d, 1H, *J* = 12.5 Hz, H-9), 3.88 (d, 1H, *J* = 12.5 Hz, H-9),

3.81 (s, 6H, H-12), 3.79 (s, 3H, H-15), 3.48 (t, 1H, *J* = 7.6 Hz, H-4), 2.21 (m, 2H, H-3), 1.44 (s, 9H, H-18/20), 1.43 (s, 9H, H-18/20) ppm

^{13}C NMR (175 MHz, $CDCl_3$) δ 172.3 (C-5), 171.2 (C-1), 160.5 (C-14), 159.1 (C-16), 158.9 (C-11), 132.0 (C-7), 118.2 (C-8), 106.7 (C-10), 90.5 (C-13), 81.8 (C-17/19), 79.6 (C-17/19), 65.7 (C-6), 55.7 (C-12), 55.2 (C-15), 53.0 (C-2), 44.3 (C-4), 34.0 (C-3), 28.3 (C-18/20), 27.9 (C-18/20), 24.1 (C-9) ppm

HRMS (ESI): calcd. for $C_{27}H_{41}NNaO_9S$ $[M+Na]^+$ 578.2394; found 578.2394.

IR (DCM cast film): 3365, 2977, 2938, 2839, 1728, 1610, 1595, 1498, 1456 cm^{-1}

$[\alpha]^{25}_{D}$ –35.6° (c 0.67, DCM).

7.5 1-Allyl 5-(*tert*-butyl) (2*S*,4*S*)-4-((*tert*-butoxycarbonyl)amino)-2-(methyldisulfaneyl)-pentanedioate (S6)

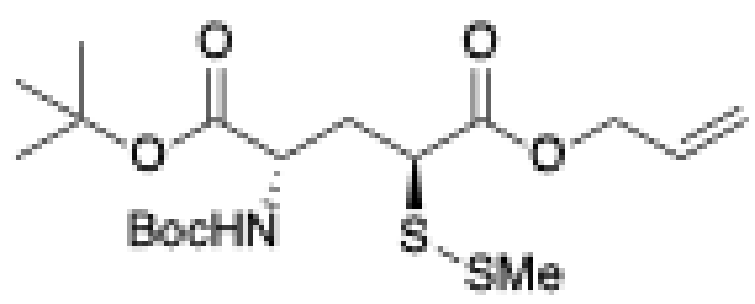

S6

S5 (1.00 g, 1.80 mmol) was added to a solution of methyl thiomethylsulfonium tetrafluoroborate (0.39 g, 1.98 mmol) in dichloromethane (20 mL) at room temperature. The reaction was stirred for 20 minutes before quenching with water (5 mL) followed by saturated aqueous sodium bicarbonate (5 mL). The solution was extracted with dichloromethane (3 × 5 mL) and the combined organics dried (Na_2SO_4), filtered, and the solvent evaporated. The product was purified by column chromatography (10-20% EtOAc/hexanes) to give the product **S6** (0.50 g, 66%) as a yellow oil

^{1}H NMR (600 MHz, $CDCl_3$) δ 5.94 (ddt, *J* = 17.2, 10.4, 5.8 Hz, 1H, $CH_2CH{=}CH_2$), 5.38 (dq, *J* = 17.2, 1.5 Hz, 1H, $CH_2CH{=}CH_2$), 5.27 (dq, *J* = 10.4, 1.3 Hz, 1H, $CH_2CH{=}CH_2$), 4.94 (d, *J* = 8.9 Hz, 1H, N*H*), 4.67 (ddt, *J* = 13.2, 5.8, 1.4 Hz, 1H, $CH_2CH{=}CH_2$), 4.61 (ddt, *J* = 13.2, 5.7, 1.4 Hz, 1H, $CH_2CH{=}CH_2$), 4.37 (s, 1H, α-C*H*), 3.51 (dd, *J* = 7.9, 6.5 Hz, 1H, γ-C*H*), 2.43 (s, 3H, -SSCH_3), 2.38 – 2.29 (m, 1H, CH_2), 2.22 – 2.15 (m, 1H, CH_2), 1.47 (s, 9H, $C(CH_3)_3$), 1.44 (s, 9H, $C(CH_3)_3$)

^{13}C NMR (126 MHz, $CDCl_3$) δ 171.3 (*C*=O), 171.2 (*C*=O), 155.5 (Boc *C*=O), 131.7 ($CH_2CH{=}CH_2$), 118.9 ($CH_2CH{=}CH_2$), 82.5 ($C(CH_3)_3$), 80.1 ($C(CH_3)_3$), 66.1 ($CH_2CH{=}CH_2$), 52.1 (α-*C*H), 48.4 (γ-*C*H), 33.7 (CH_2), 28.3 ($C(CH_3)_3$), 28.0 ($C(CH_3)_3$), 23.6 (SS*C*H3)

HRMS (ESI^+) 444.1483 $[M + Na]^+$ ($C_{18}H_{31}NNaO_6S_2$ requires 444.1485)

IR (ν_{max} / cm^{-1}) 3372, 2979, 2932, 1734, 1505, 1368, 1256, 1154

OR -42.04 (c = 0.50 g/100 mL).

7.6 5-Allyl 1-(*tert*-butyl) (2*S*,4*S*)-2-((((9*H*-fluoren-9-yl)methoxy)carbonyl)amino)-4-(methyl-disulfaneyl)pentanedioate (S7)

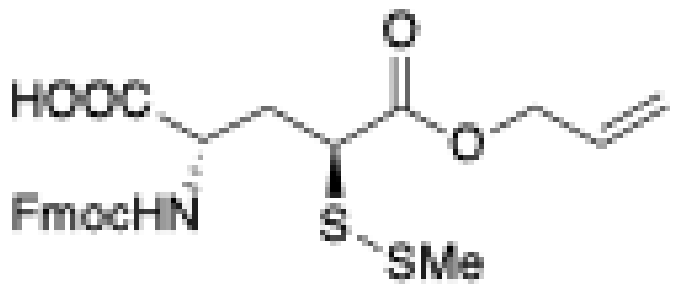

S7

Allyl ester **S6** (200 mg, 0.474 mmol) was dissolved in a solution of TFA/TIPS/H_2O (9.5:0.25:0.25, 2 mL) and left to stir at room temperature for 6 h. The solvent was evaporated under reduced pressure and the resulting residue redissolved in water (5 mL) and solid sodium bicarbonate added until pH 9. A solution of FmocOSu (192 mg, 0.569 mmol) in 1,4-dioxane (5 mL) was added and the solution left to stir at room temperature. After 24 h, the reaction was quenched with HCl (1 M, 5 mL) then extracted with ethyl acetate (3 × 10 mL) and the combined organics dried (Na_2SO_4), filtered, and the solvent evaporated under reduced pressure. The product was purified by column chromatography to give the product **S7** (0.211 g, 82%) as a colourless oil

^{1}H NMR (500 MHz, $CDCl_3$) δ 7.75 (d, *J* = 7.5 Hz, 2H, FmocC*H*), 7.59 (t, *J* = 7.4 Hz, 2H, FmocC*H*), 7.39 (t, *J* = 7.4 Hz, 2H, FmocC*H*), 7.31 (t, *J* = 7.5 Hz, 2H, FmocC*H*), 5.91 (ddt, *J* = 16.4, 10.8, 5.7 Hz, 1H, C*H*=CH_2), 5.48 (s, 1H, N*H*), 5.35 (d, *J* = 17.2 Hz, 1H, CH=CH_2), 5.24 (d, *J* = 10.3 Hz, 1H, CH=CH_2), 4.62 (m, 3H CH_2CH=CH_2 & C*H*NHFmoc), 4.42 (m, 2H, FmocCH_2), 4.21 (t, *J* = 6.9 Hz, 1H, FmocC*H*), 3.52 (m, 1H, C*H*SSCH_3), 2.54 (s, 1H, CH_2), 2.39 (m, 4H, SCH_3 & CH_2)

^{13}C NMR (126 MHz, $CDCl_3$) δ 176.0 (*C*O_2H), 171.3 (*C*O_2Allyl), 156.4 (Fmoc *C*=O), 143.8 (Fmoc*C*), 143.6 (Fmoc*C*), 141.3 (Fmoc*C*), 131.5 (*C*H=CH_2), 127.8 (Ar*C*H), 127.8 (Ar*C*H), 127.2 (Ar*C*H), 125.2 (Ar*C*H), 125.1 (Ar*C*H), 120.1 (Ar*C*H), 119.1 (CH=*C*H_2), 67.4 (Fmoc*C*H_2), 66.3 (*C*H_2CH=CH_2), 51.9 (*C*HNHFmoc), 48.2 (*C*HSSCH_3), 47.1 (Fmoc*C*H), 32.8 (*C*H_2), 23.6 (S*C*H_3)

HRMS (ESI$^+$) 510.1010 $[M + Na]^+$ ($C_{24}H_{25}NNaO_6S_2$ requires 510.1016)

IR (ν_{max} / cm^{-1}) 3326, 3066, 3046, 3019, 2956, 2920, 1730, 1525, 1450, 1272, 1231, 1052, 759, 741

OR -25.25 (c = 0.67 g/100 mL).

8) Synthesis of MccJ25 analog from bis-disulfide/ γ-thiol Glu strategy

8.1 Disulfide-containing MccJ25 analog with γ-thiol-Glu (70)

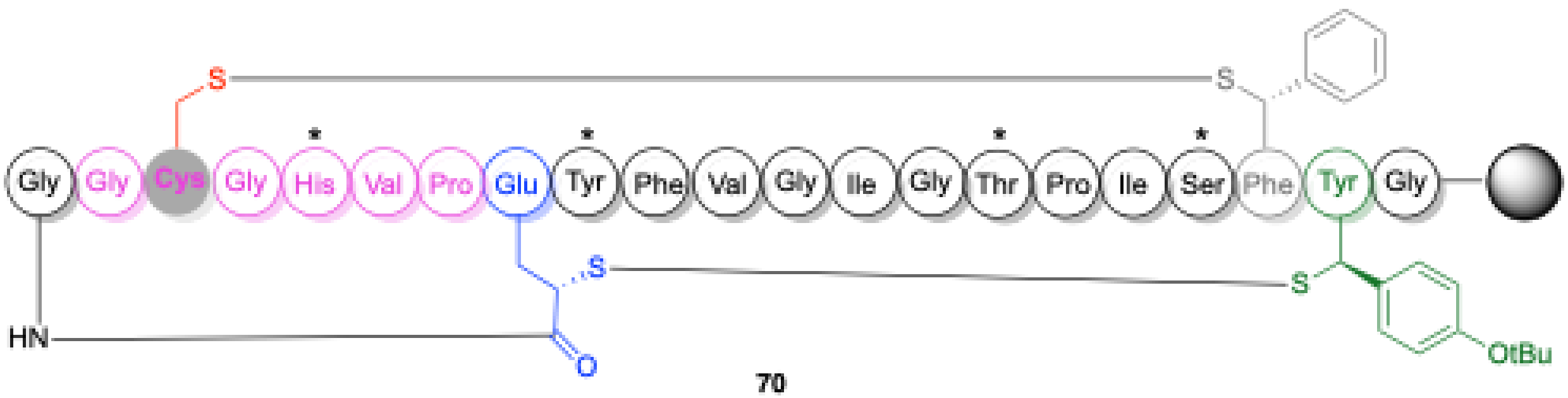

70

Synthetic MccJ25 analog **67** was synthesized according to the general SPPS protocol. Orthogonally protected β-thiol Tyr **57** was incorporated into position 20 by reduced loading procedure. Fmoc-β-thiol-Phe(Mmt)-OH **61** was (3 equiv) incorporated into position 19 using HATU (2.9 equiv), HOAt (2.9 equiv), and DIPEA (15 equiv), and allowed to bubble with argon for 3 hours. Orthogonally protected γ-thiol Glu (2 equiv) **S7** was incorporated into position 8 using DIC (2 equiv) and HOBt (2 equiv), and allowed to bubble with argon for 3 hours. Fmoc-Cys(Mmt)-OH was incorporated into position 3 according to the general SPPS protocol. After incorporation of 21 amino acids on the resin, bis-disulfide formation was performed. Allyl deprotection was performed, followed by Fmoc removal to obtain peptide for macrocyclization. Side chain to tail cyclization was performed according to general cyclization procedure to afford peptide **70**.

8.2 Macrolactam ring and disulfide-containing MccJ25 analog with γ-thiol-Glu (71)

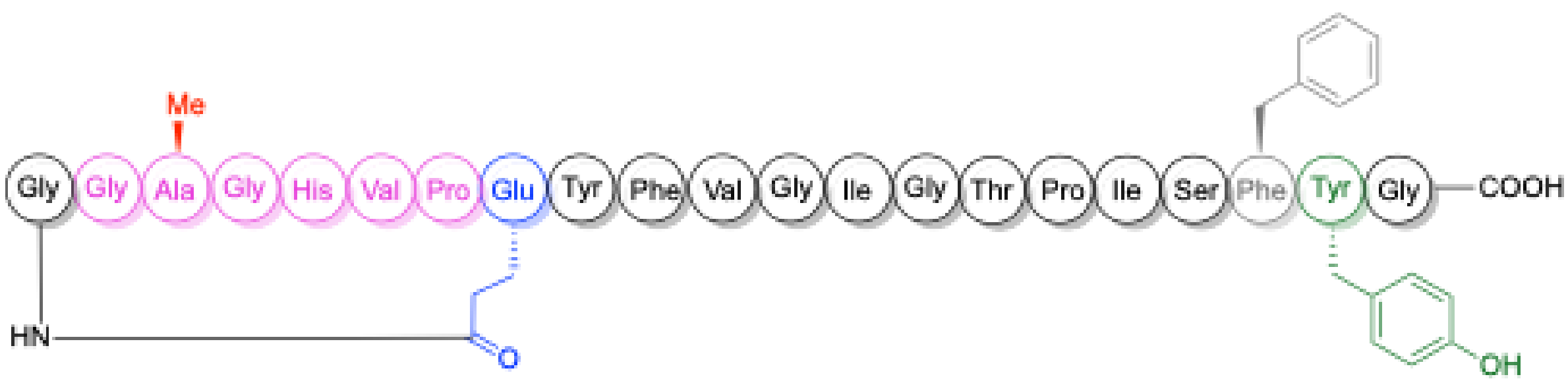

71

Bis-disulfide containing peptide **70** was cleaved off the resin and subjected to disulfide reduction and desulfurization according to protocol 1.4.8 to afford MccJ25 analog **71**.

9) Synthetic MccJ25 analogues purification and characterization

9.1 Synthetic loop-tail MccJ25 analog (S8)[6]

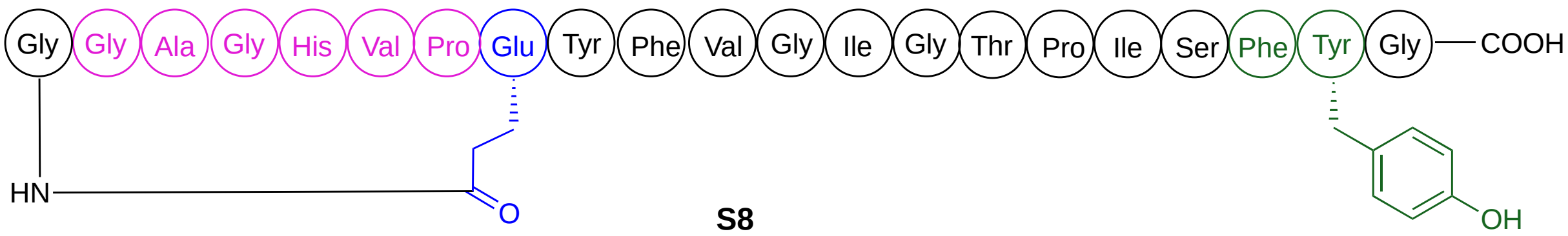

S8

Close-loop MccJ25 analog **S8** was synthesized according to the general SPPS protocol. Fmoc-Tyr(O*t*Bu)-OH was incorporated into position 20 by reduced loading procedure. Fmoc-Glu(OAll)-OH was incorporated into position 8 according to the general SPPS protocol. After incorporation of 21 amino acids on the resin, allyl/alloc deprotection was performed, followed by macrocyclization protocol. The peptide was then cleaved off the resin and purified by semi-preparative RP-HPLC following the standard close-loop protocol (retention time = 23.2 min) to give peptide **S8** (1.68 mg, 8% yield, relative to 100 mg of crude peptide) as a fluffy white solid.

LCMS (ESI–TOF) $[M+2H]^{2+}$ calc'd for $C_{101}H_{141}N_{23}O_{27}$ 1054.0178, found 1054.0192.

9.2 Synthetic MccJ25 analog from bis-disulfide/ *β*-thiol-Glu (66)

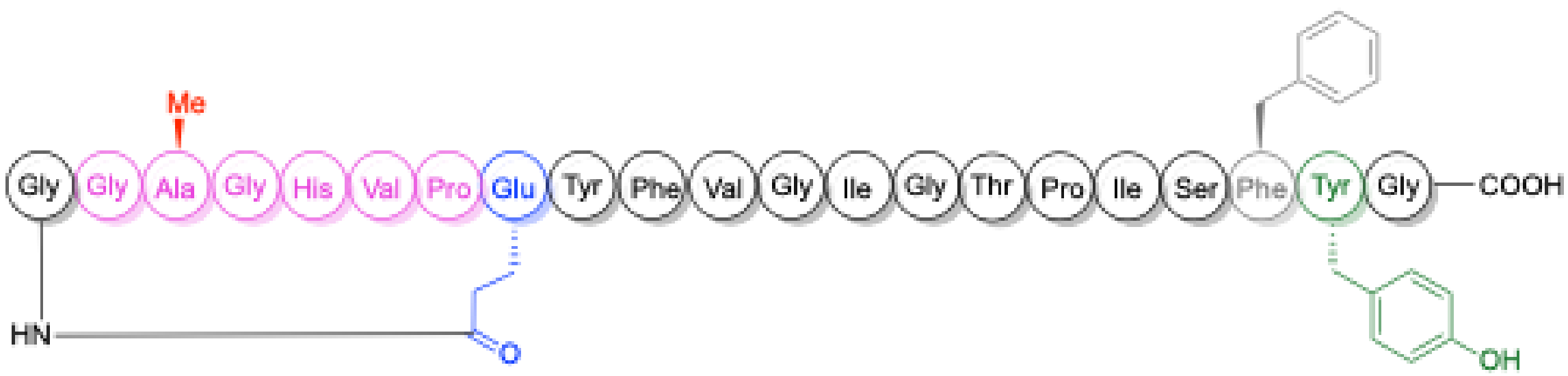

66

After desulfurization, synthetic MccJ25 analog was purified by semi-preparative RP-HPLC following the standard close-loop protocol (retention time = 23.3 min) to give peptide **66** (0.63 mg, 3% yield, relative to 100 mg of crude peptide) as a fluffy white solid.

9.3 Synthetic MccJ25 analog from bis-disulfide/ γ -thiol-Glu (71)

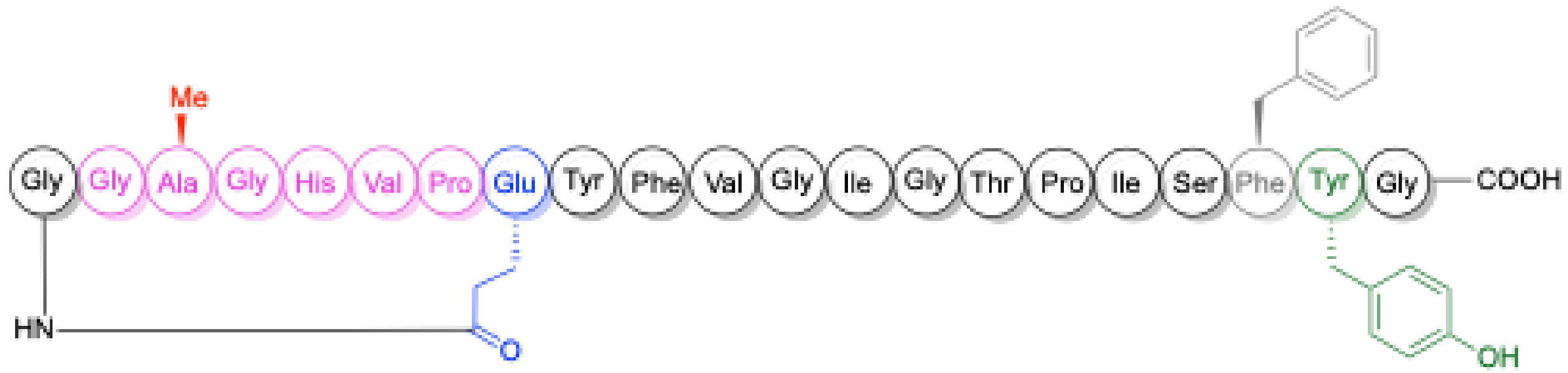

71

After desulfurization, synthetic MccJ25 analog was purified by semi-preparative RP-HPLC following the standard close-loop protocol (retention time = 23.3 min) to give peptide **71** (0.40 mg, 2% yield, relative to 100 mg of crude peptide) as a fluffy white solid.

10) Synthetic MccJ25 analogues intermediates characterization

10.1 Disulfide-containing MccJ25 analog with β-thiol-Glu (72)

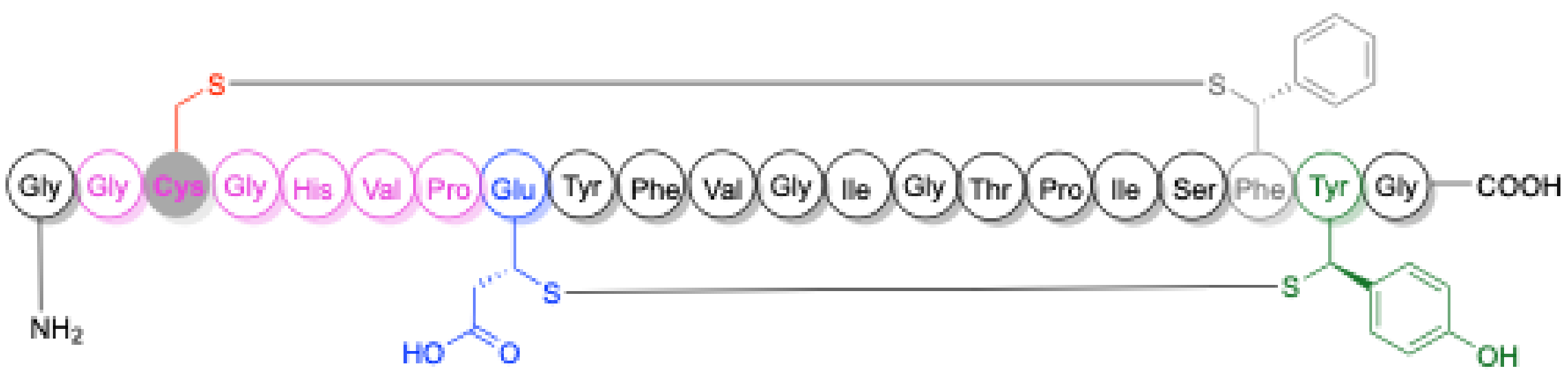

72

Synthetic MccJ25 analog **62** was synthesized according to the general SPPS protocol. Orthogonally protected β-thiol Tyr **57** was incorporated into position 20 by reduced loading procedure. Fmoc-β-thiol-Phe(Mmt)-OH **61** was (3 equiv) incorporated into position 19 using HATU (2.9 equiv), HOAt (2.9 equiv), and DIPEA (15 equiv), and allowed to bubble with argon for 3 hours. Orthogonally protected β-thiol Glu **54** (2 equiv) was incorporated into position 8 using DIC (2 equiv) and HOBt (2 equiv), and allowed to bubble with argon for 3 hours. Fmoc-Cys(Mmt)-OH was incorporated into position 3 according to the general SPPS protocol. After incorporation of 21 amino acids on the resin, bis-disulfide formation was performed to give peptide **64**. Allyl deprotection was performed, followed by Fmoc removal to obtain bis-disulfide containing intermediate peptide without macrocycle ring. The peptide was cleaved off the resin and purified by RP-HPLC following bis-disulfide and macrolactam ring containing intermediate peptide purification method (retention time = 14.1 min) to give peptide

72 (0.21 mg, 1% yield, relative to 100 mg of crude peptide) as a fluffy white solid. LCMS (ESI–TOF) $[M+2H]^{2+}$ calc'd for $C_{101}H_{139}N_{23}O_{28}S_4$ 1124.9516, found 1124.9513.

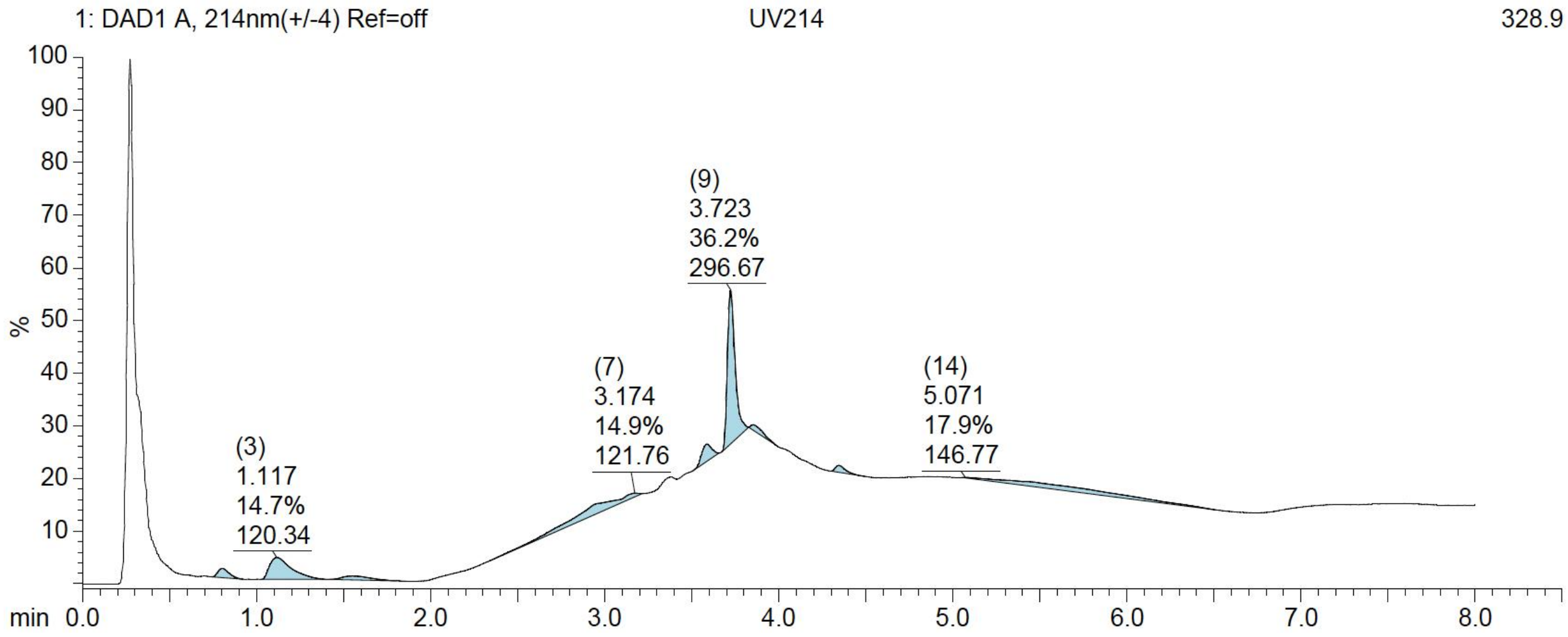

LCMS trace of peptide **72**

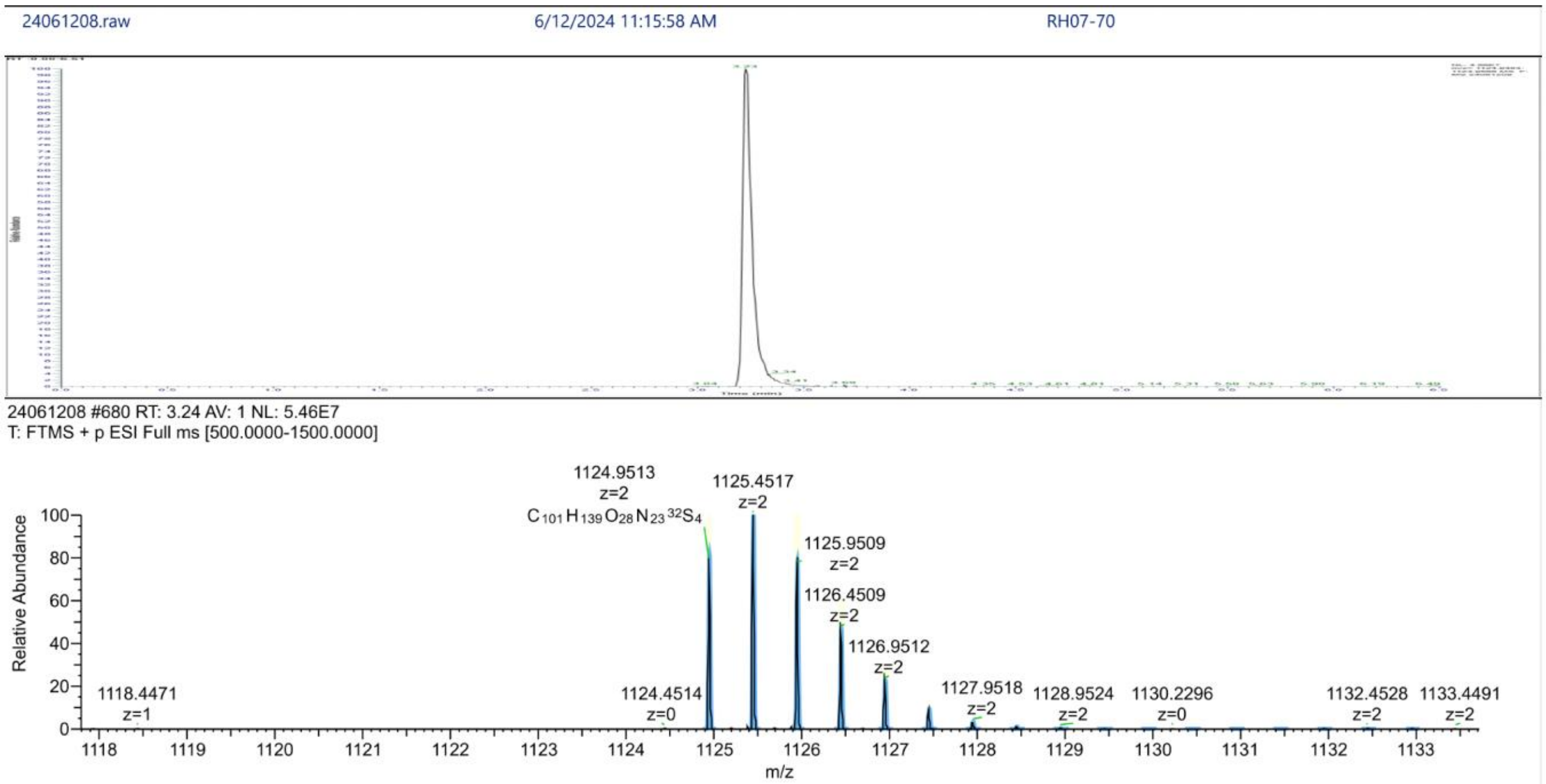

Peak Mass	Display For...	S Fit	RDB	Delta [ppm]	Theo. mass	Rank	Combined S...	# Matched I...	# Missed Iso.	MS Cov. [%]	Pattern Cov....	MSMS Matc...
1124.9513	$C_{101}H_{139}O_{28}N_{23}{}^{32}S_4$	51.7754249067613	44.00	-0.26	1124.95159	1	97.07	9	0	99.59	99.93	(Collection)

LCMS (ESI–TOF) of peptide **72**

10.2 Macrolactam ring and disulfide-containing MccJ25 analog with β-thiol-Glu (73)

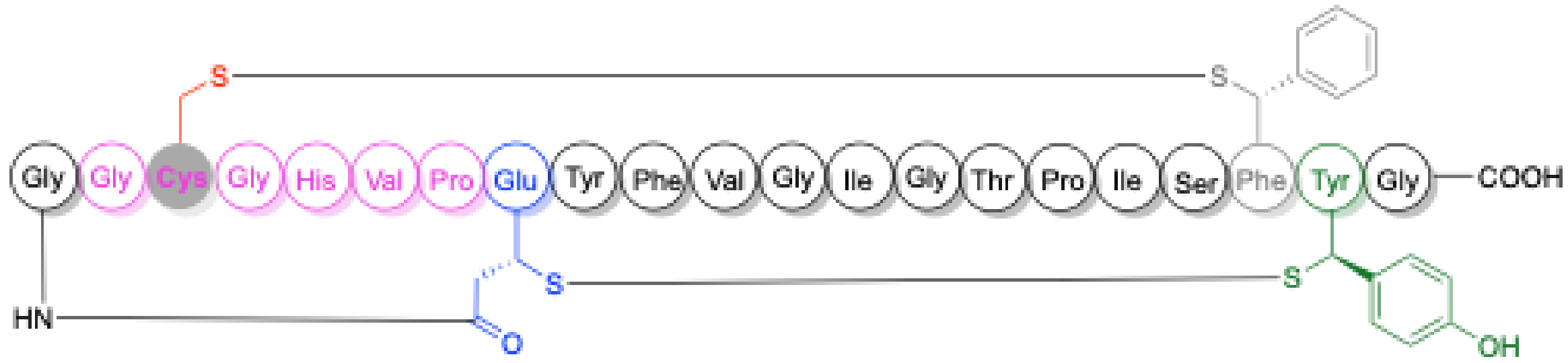

73

Peptide **65** was synthesized as described, and it was cleaved from the resin. After cyclization, the peptide was cleaved off the resin and purified by RP-HPLC using following bis-disulfide and macrolactam ring containing intermediate peptide purification method (retention time = 16.2 min) to give peptide **73** (0.21 mg, 1% yield, relative to 100 mg of crude peptide) as a fluffy white solid. LCMS (ESI–TOF) $[M+2H]^{2+}$ calc'd for $C_{101}H_{137}N_{23}O_{27}S_4$ 1115.9463, found 1115.9454.

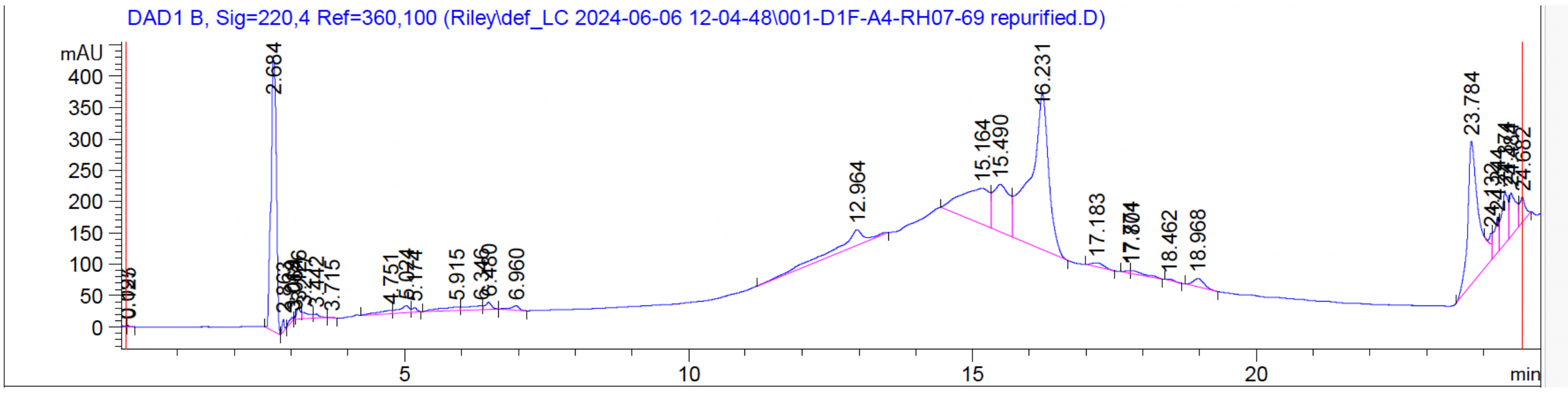

HPLC trace of peptide **73**

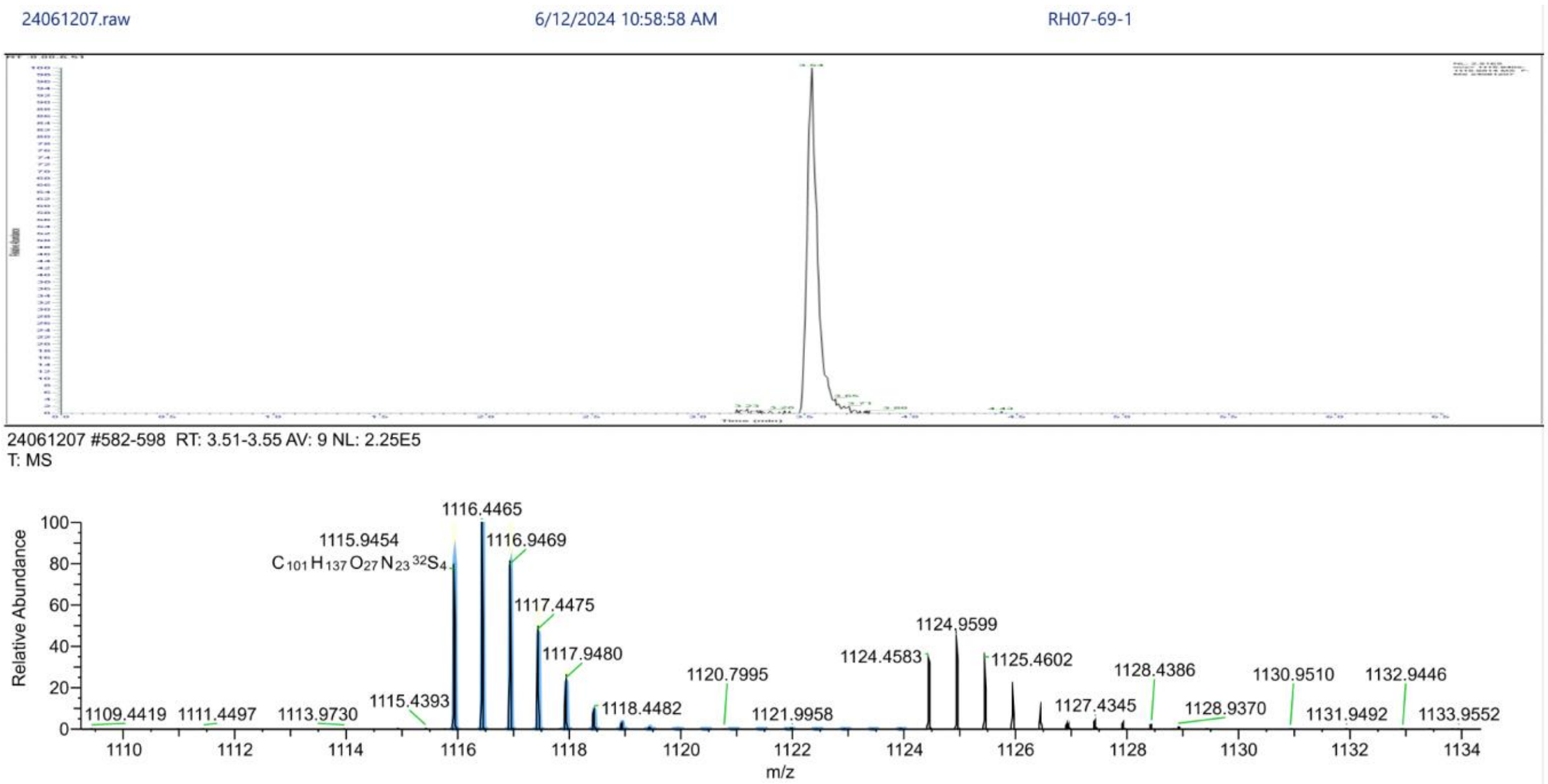

Peak Mass	Display For...	S Fit	RDB	Delta [ppm]	Theo. mass	Rank	Combined S...	# Matched I...	# Missed Iso.	MS Cov. [%]	Pattern Cov....	MSMS Matc...
1115.9454	$C_{101}H_{137}O_{27}N_{23}{}^{32}S_4$	50.75567679 51558	45.00	-0.81	1115.94631	1	97.17	8	1	99.75	99.65	(Collection)

LCMS (ESI–TOF) of peptide **73**

10.3 Disulfide-containing MccJ25 analog with γ-thiol-Glu (74)

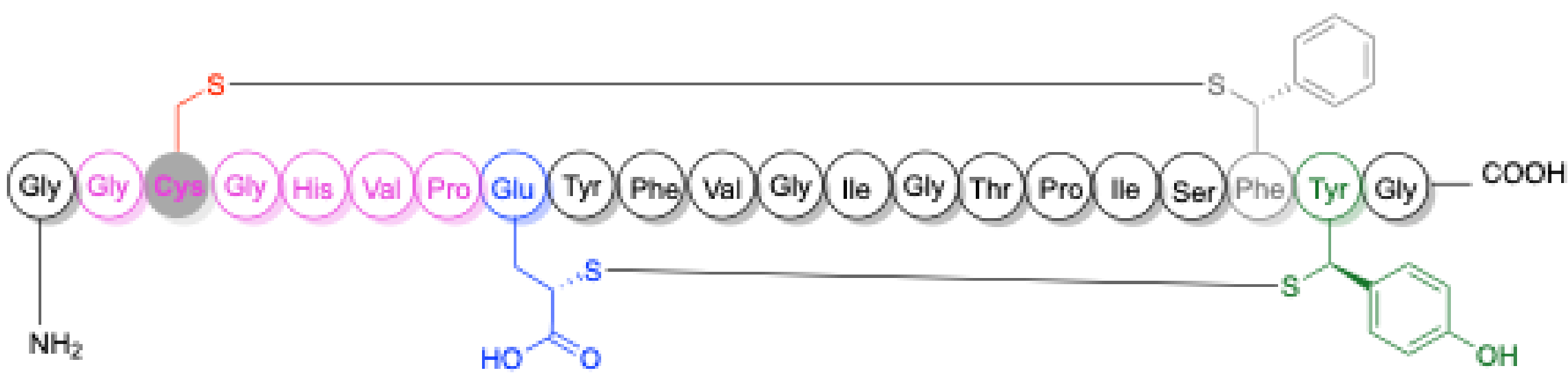

74

Synthetic MccJ25 analog **67** was synthesized according to the general SPPS protocol. Orthogonally protected β-thiol Tyr **57** was incorporated into position 20 by reduced loading procedure. Fmoc-β-thiol-Phe(Mmt)-OH **61** was (3 equiv) incorporated into position 19 using HATU (2.9 equiv), HOAt (2.9 equiv), and DIPEA (15 equiv), and allowed to bubble with argon for 3 hours. Orthogonally protected γ-thiol Glu (2 equiv) **S7** was incorporated into position 8 using DIC (2 equiv) and HOBt (2 equiv), and allowed to bubble with argon for 3 hours. Fmoc-Cys(Mmt)-OH was incorporated into position 3 according to the general SPPS protocol. After incorporation of 21 amino acids on the resin, bis-disulfide formation was performed. Allyl deprotection was performed, followed by Fmoc removal to obtain peptide for macrocyclization. Side chain to tail cyclization was performed according to general cyclization procedure to afford peptide **74**. Allyl deprotection was performed, followed by

Fmoc removal to obtain bis-disulfide containing intermediate peptide without macrocycle ring. The peptide was cleaved off the resin and purified by RP-HPLC following bis-disulfide and macrolactam ring containing intermediate peptide purification method (retention time = 14.5 min) to give peptide **74** (0.21 mg, 1% yield, relative to 100 mg of crude peptide) as a fluffy white solid. LCMS (ESI–TOF) $[M+2H]^{2+}$ calc'd for $C_{101}H_{139}N_{23}O_{28}S_4$ 1124.9516, found 1124.9523.

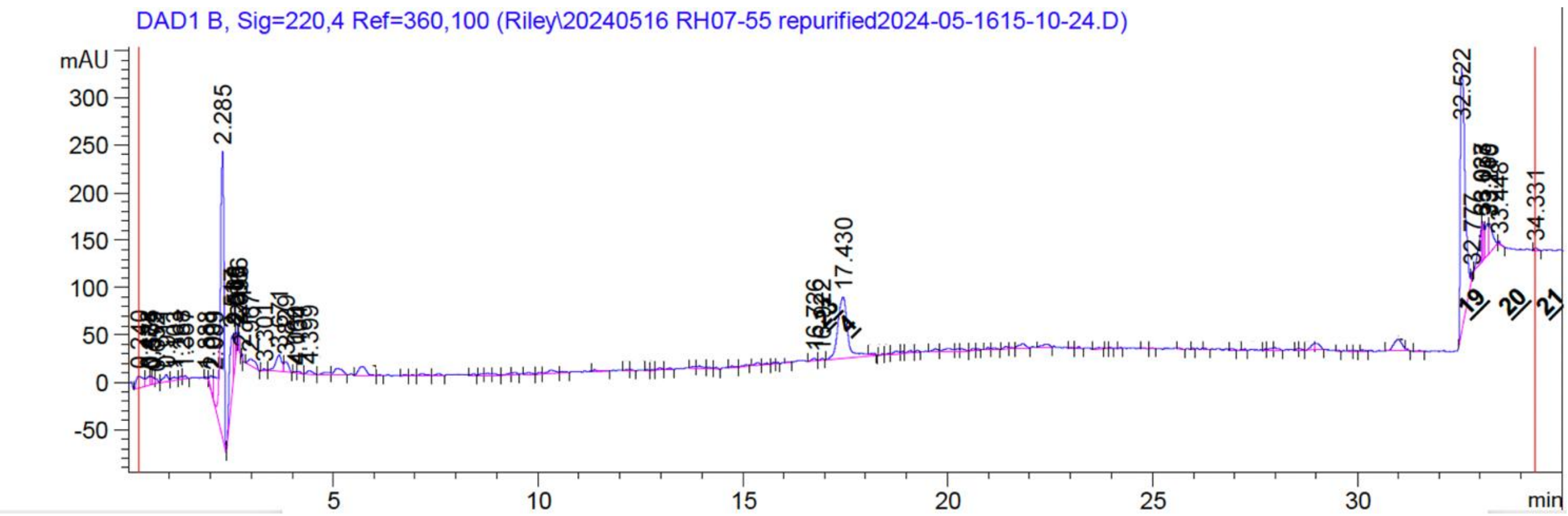

HPLC trace of peptide **74**

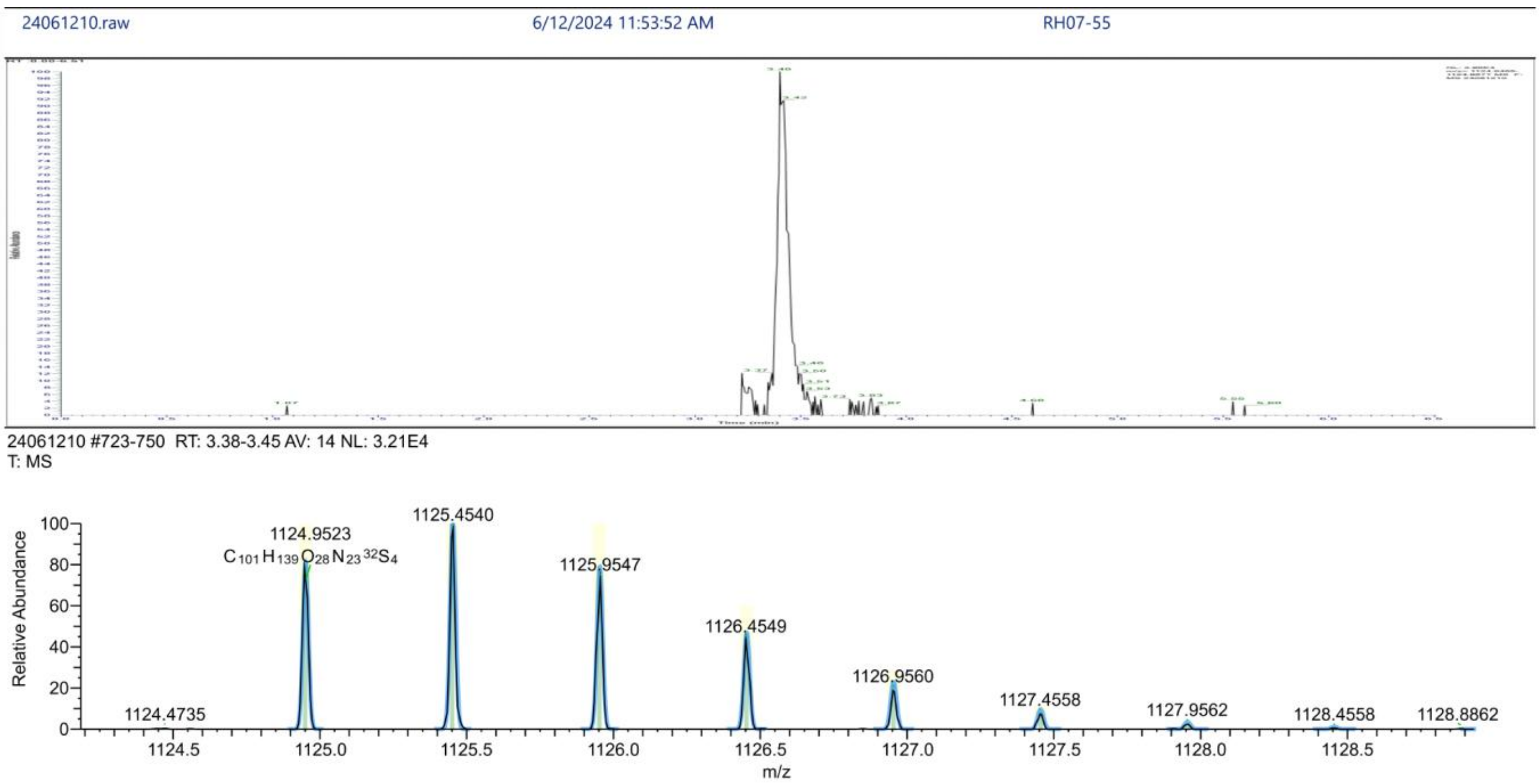

Peak Mass	Display For...	S Fit	RDB	Delta [mmu]	Theo. mass	Rank	Combined S...	# Matched I...	# Missed Iso.	MS Cov. [%]	Pattern Cov....	MSMS Matc...
1124.9523	$C_{101}H_{139}O_{28}N_{23}{}^{32}S_4$	63.88018865 79312	44.00	0.71	1124.95159	1	97.88	8	0	99.77	100	(Collection)

LCMS (ESI–TOF) of peptide **74**

10.4 Macrolactam ring and disulfide-containing MccJ25 analog with γ-thiol-Glu (75)

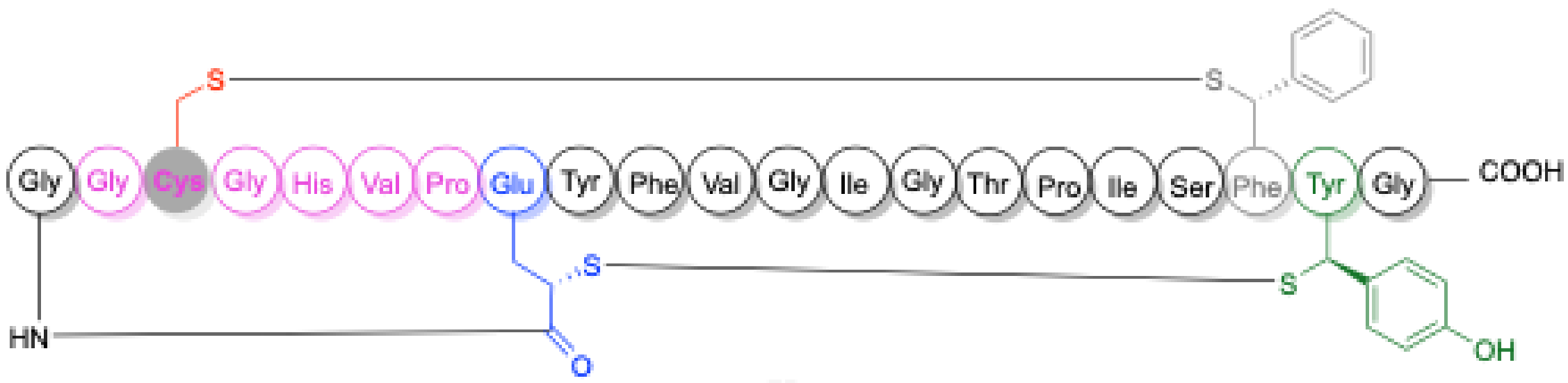

75

Peptide **70** was synthesized as described, and it was cleaved from the resin. After cyclization, the peptide was cleaved off the resin and purified by RP-HPLC using following bis-disulfide and macrolactam ring containing intermediate peptide purification method (retention time = 14.1 min) to give peptide **75** (0.21 mg, 1% yield, relative to 100 mg of crude peptide) as a fluffy white solid. LCMS (ESI–TOF) $[M+2H]^{2+}$ calc'd for $C_{101}H_{137}N_{23}O_{27}S_4$ 1115.9463, found 1115.9456.

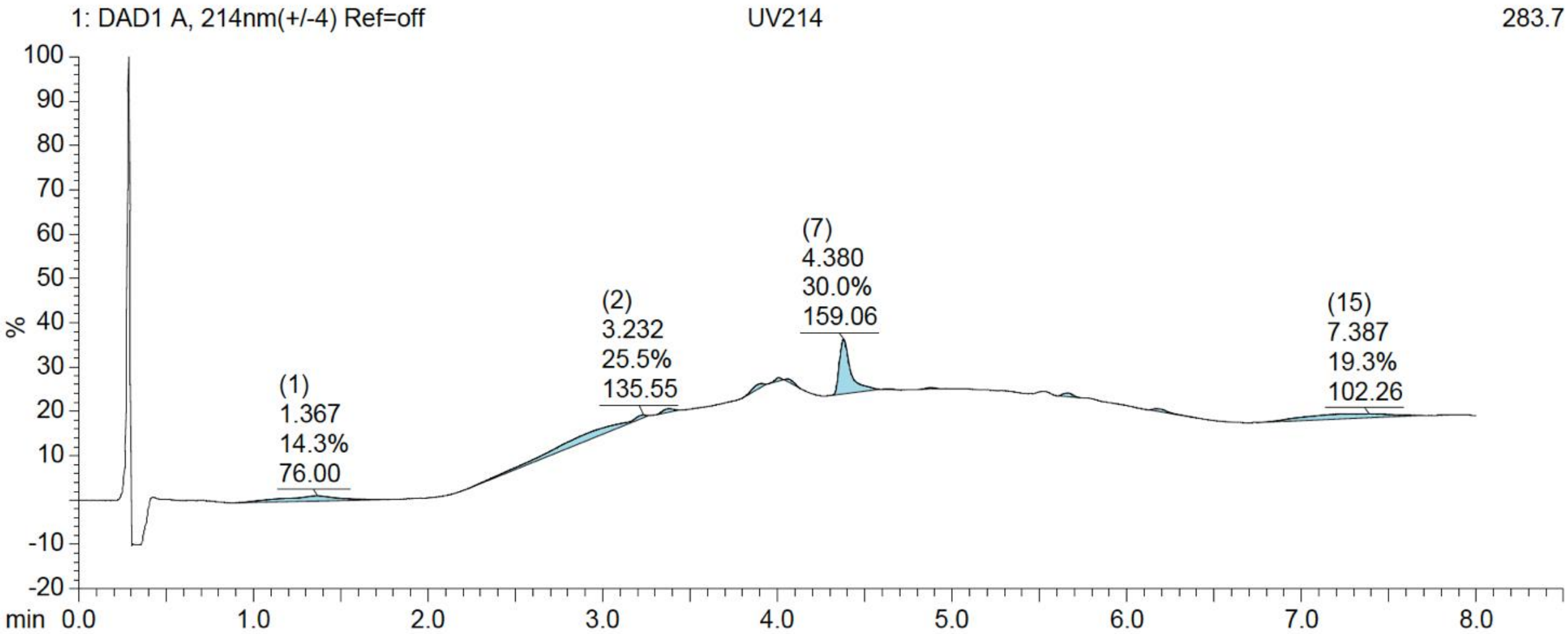

LCMS trace of peptide **75**

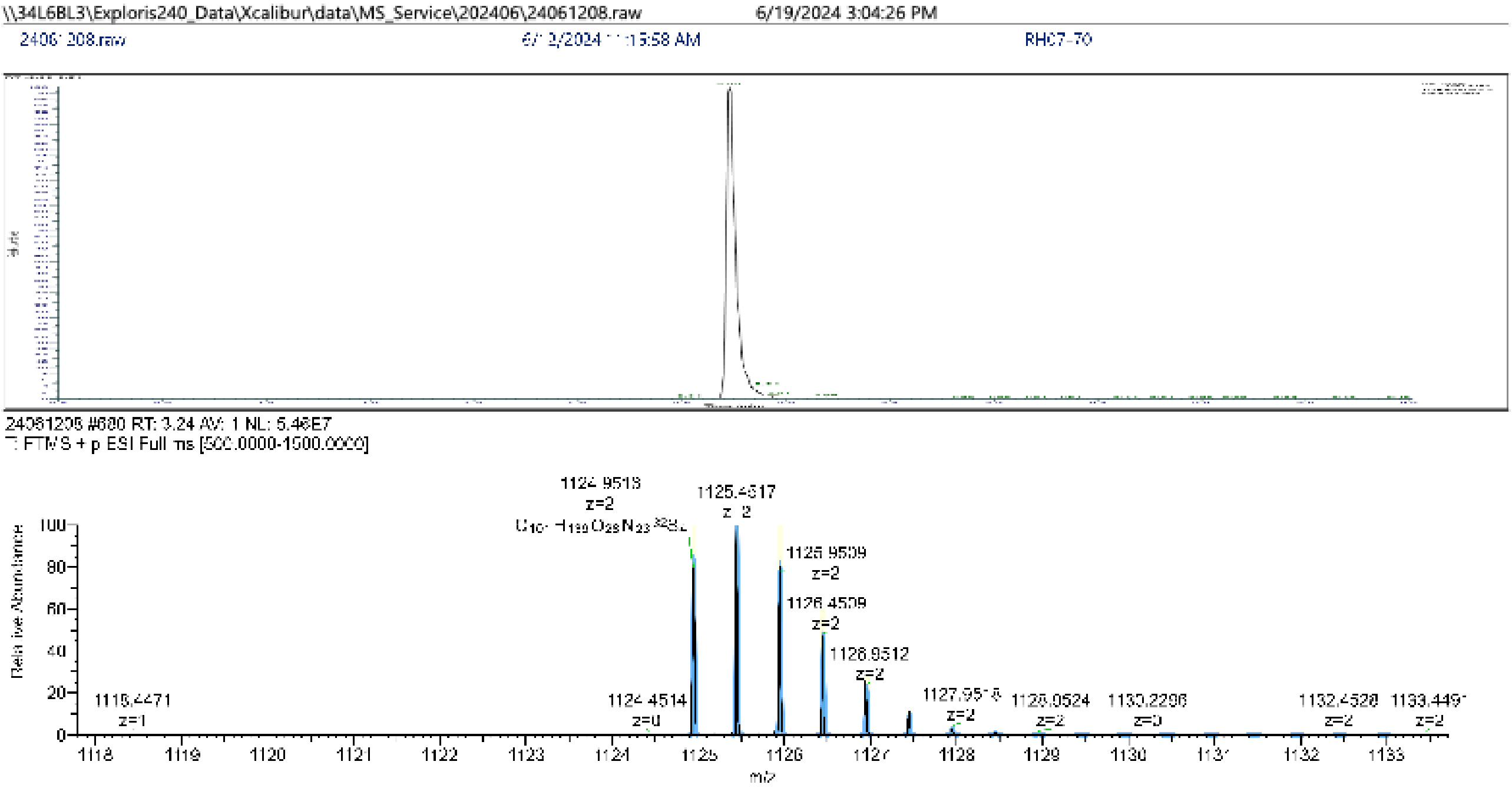

Peak Mass	Display For.	S Fi	RDB	Delta [ppm]	Theo. mass	Rank	Combined S...	# Matched I...	# Missed Iso.	MS Cov. [%]	Pattern Cov. ...	MSMS Mat...
1124.9513	$C_{101}H_{154}O_{28}N_{23}{}^{32}S_2$	51.77542490 67613	44.00	-0.26	1124.9559	1	97.07	9	0	99.53	99.93	(Collection)

LCMS (ESI–TOF) of peptide **75**

11) Bioactivity of synthetic MccJ25 analogues intermediates

A spot-on-lawn assay for 128 µM solutions of each synthetic disulfide-containing peptides **71**-**75** and the standard loop-tail **S8** against *E. coli* was conducted with polymyxin B as the positive control.[7] Unfortunately, no activity was observed at this concentration of each peptide (Figure S1).

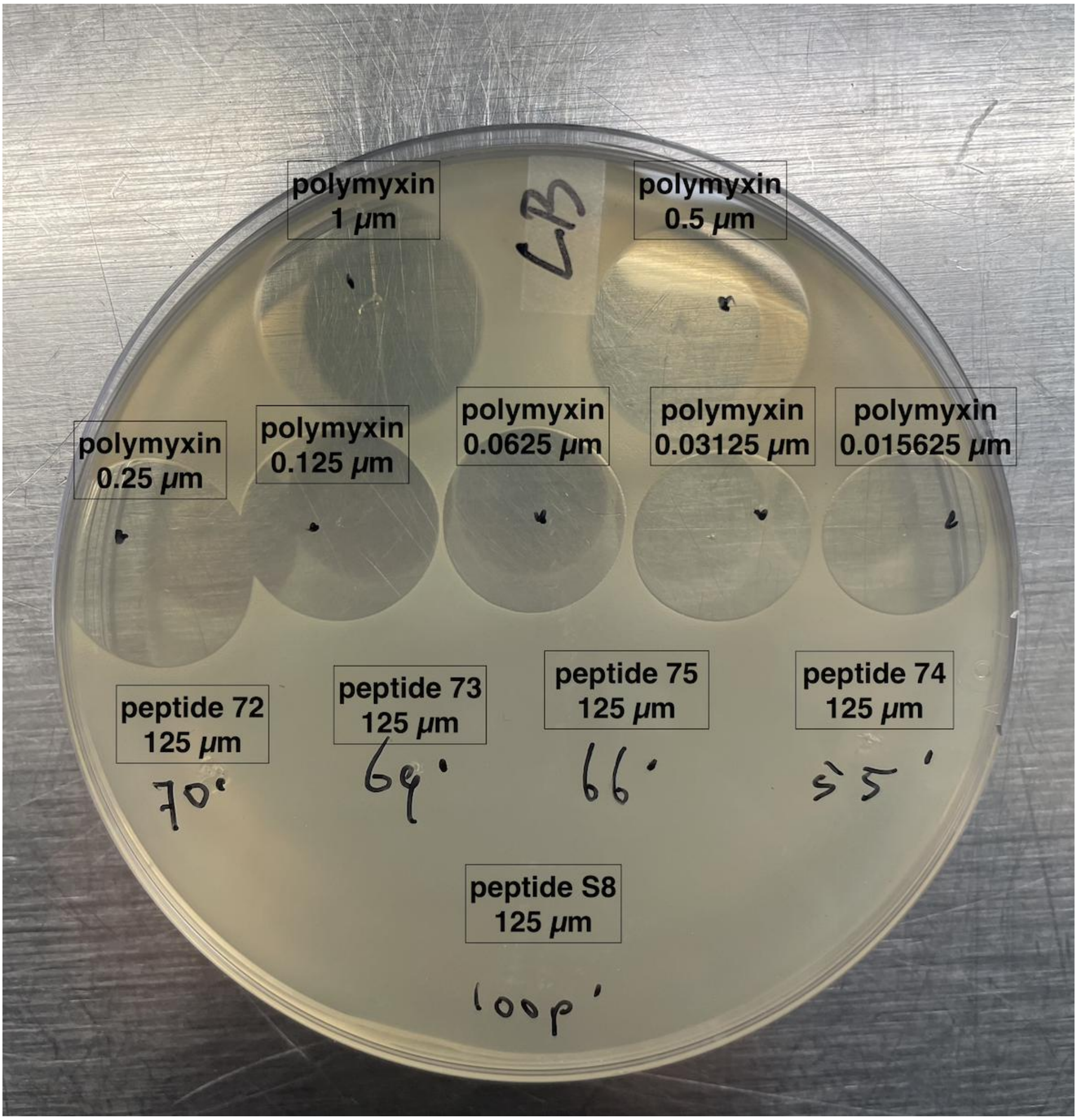

Figure S1. Spot-on-lawn assay of synthetic MccJ25 analogues

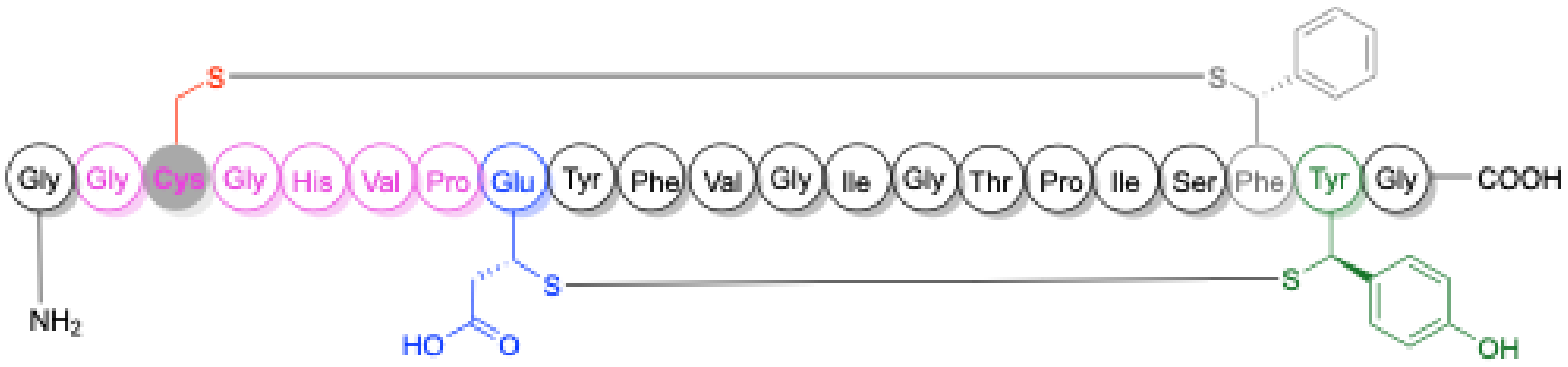
Gly
Gly
Cys
Gly
His
Val
Pro
Glu
Tyr
Phe
Val
Gly
Ile
Gly
Thr
Pro
Ile
Ser
Phe
Tyr
Gly
COOH
S
S
S
S
NH2
HO
O
OH

72

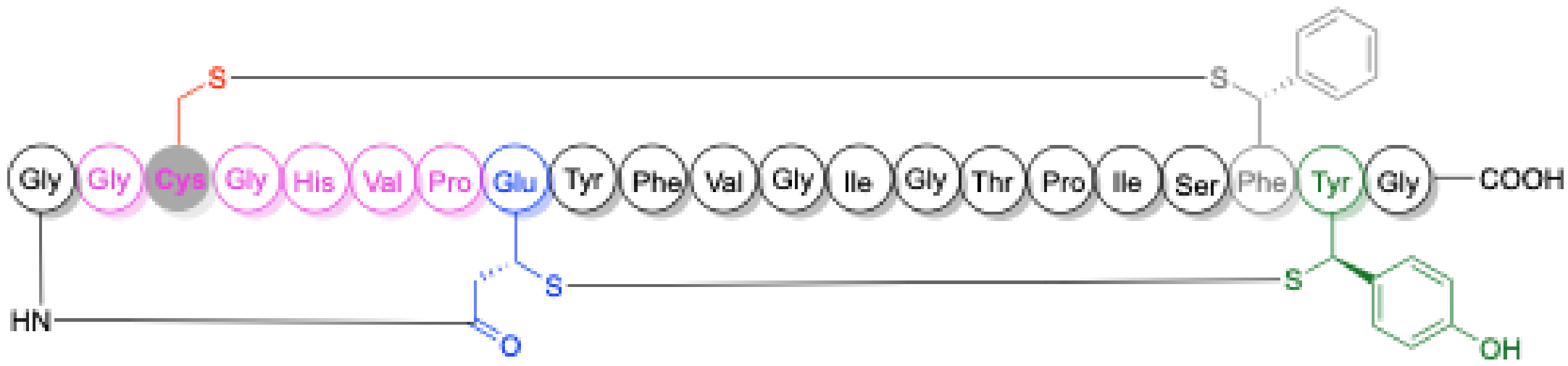
Gly
Gly
Cys
Gly
His
Val
Pro
Glu
Tyr
Phe
Val
Gly
Ile
Gly
Thr
Pro
Ile
Ser
Phe
Tyr
Gly
COOH
S
S
S
S
HN
O
OH

73

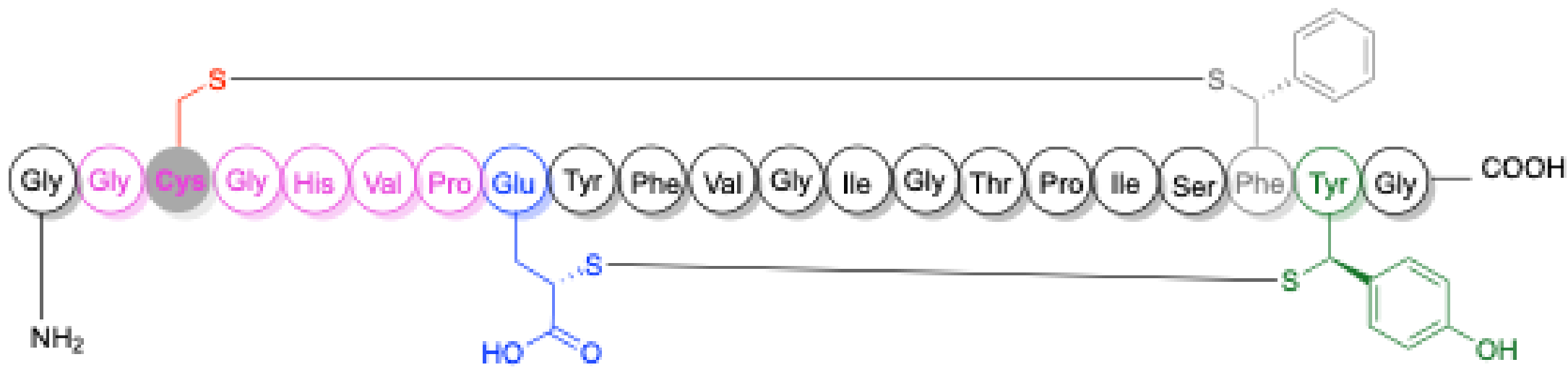
Gly
Gly
Cys
Gly
His
Val
Pro
Glu
Tyr
Phe
Val
Gly
Ile
Gly
Thr
Pro
Ile
Ser
Phe
Tyr
Gly
COOH
S
S
S
S
NH2
HO
O
OH

74

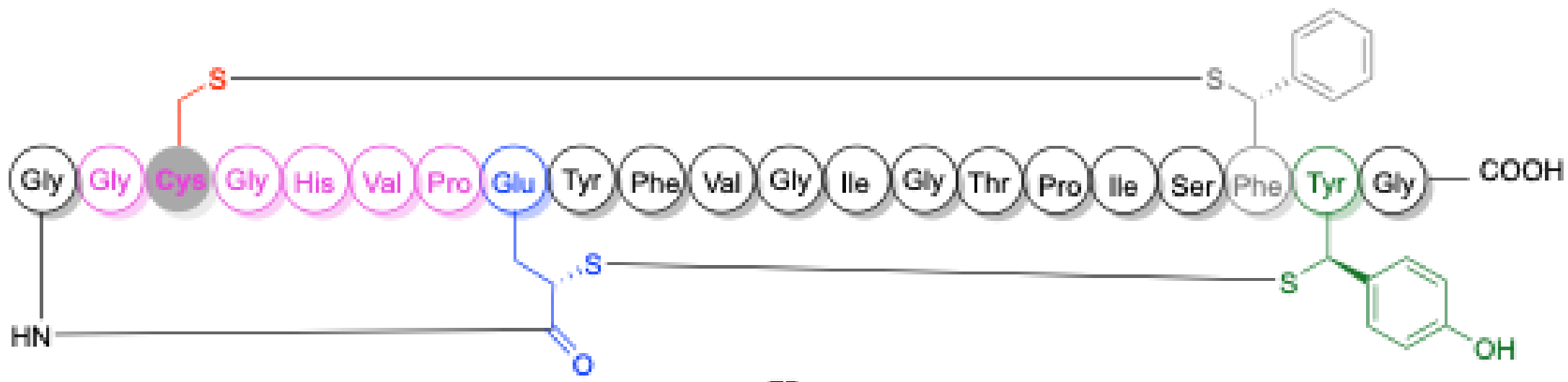
Gly
Gly
Cys
Gly
His
Val
Pro
Glu
Tyr
Phe
Val
Gly
Ile
Gly
Thr
Pro
Ile
Ser
Phe
Tyr
Gly
COOH
S
S
S
S
HN
O
OH

75

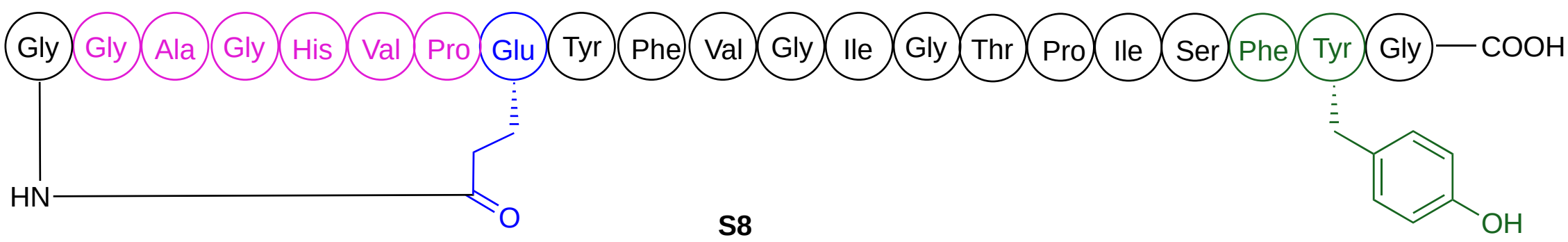
Gly
Gly
Ala
Gly
His
Val
Pro
Glu
Tyr
Phe
Val
Gly
Ile
Gly
Thr
Pro
Ile
Ser
Phe
Tyr
Gly
COOH
HN
O
OH

S8

12) MS/MS of loop-tail MccJ25 and synthetic MccJ25 analogues

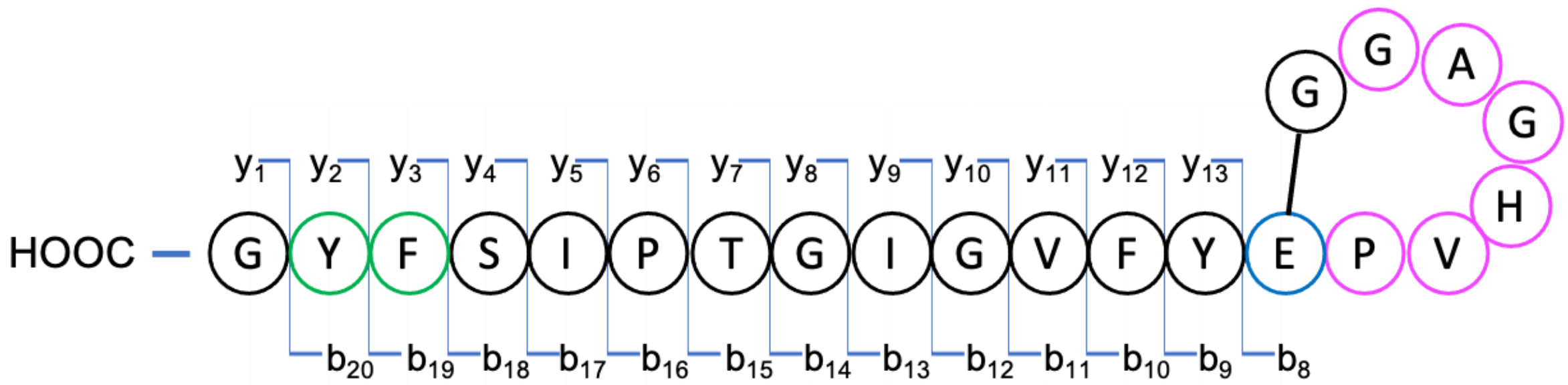

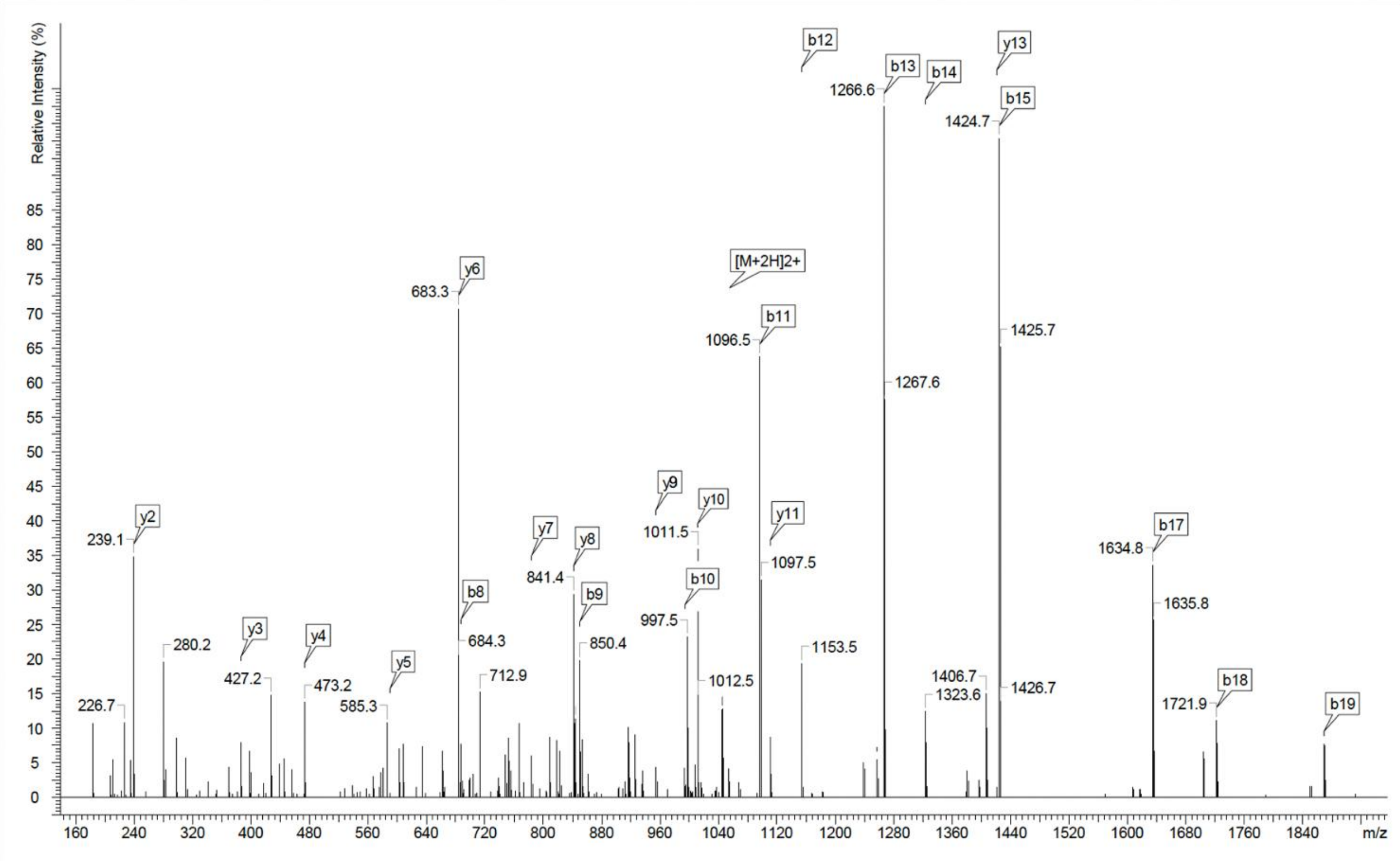

MS/MS of loop-tail synthetic MccJ25 analogues

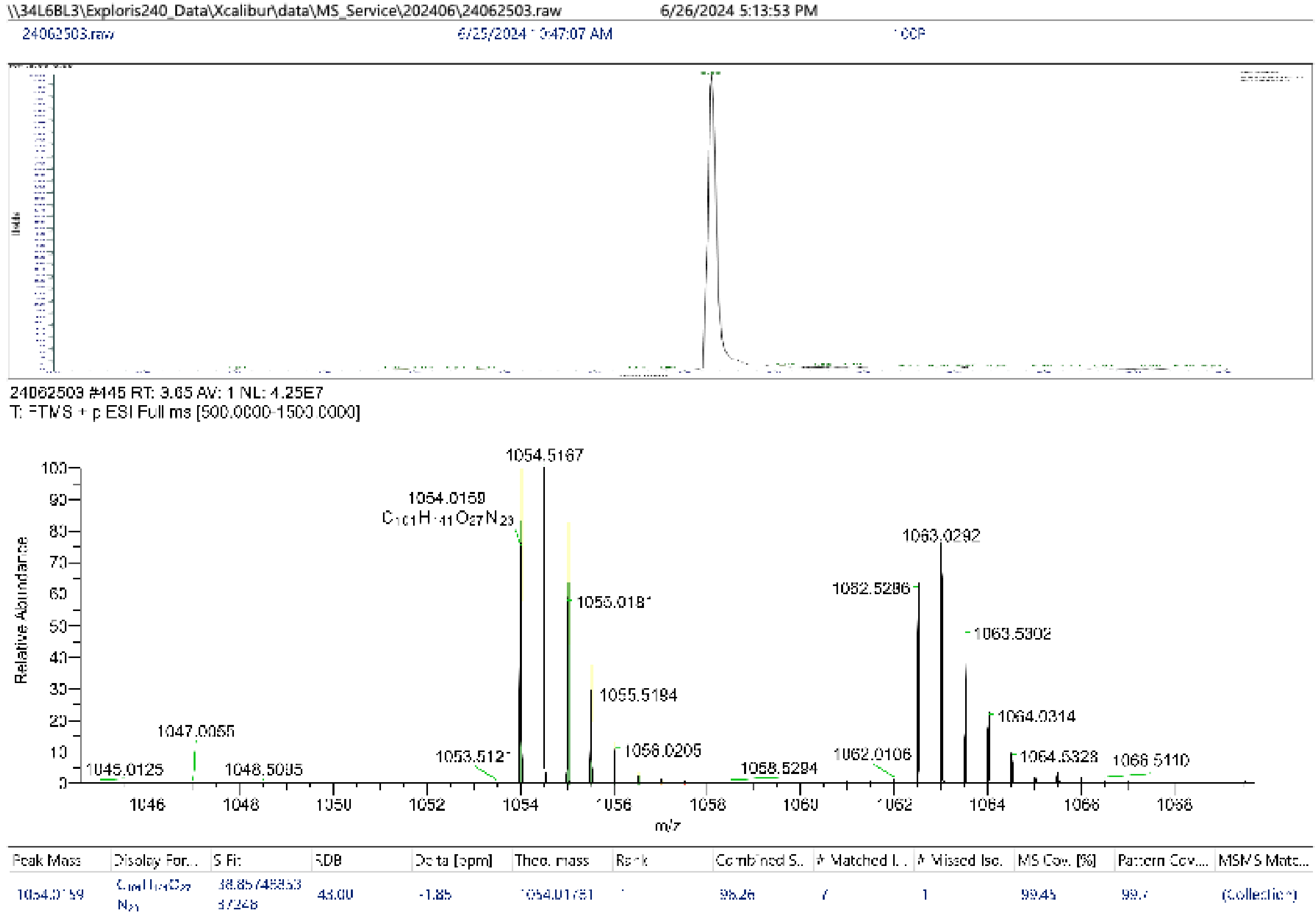

Peak Mass	Display For...	S Fit	RDB	Delta [ppm]	Theo. mass	Rank	Combined S...	# Matched I...	# Missed Iso...	MS Cov. [%]	Pattern Cov...	MSMS Matc...
1054.0159	$C_{101}H_{141}O_{27}N_{23}$	38.85745853 37248	43.00	-1.85	1054.01751	1	96.26	7	1	99.45	99.7	(Collection)

LCMS (ESI–TOF) of loop-tail synthetic MccJ25 analogues

13) Determination of disulfide connectivity of synthetic MccJ25 analogues intermediates

13.1 Synthesis of Nem/Nmm alkylated MccJ25 analog (S10)

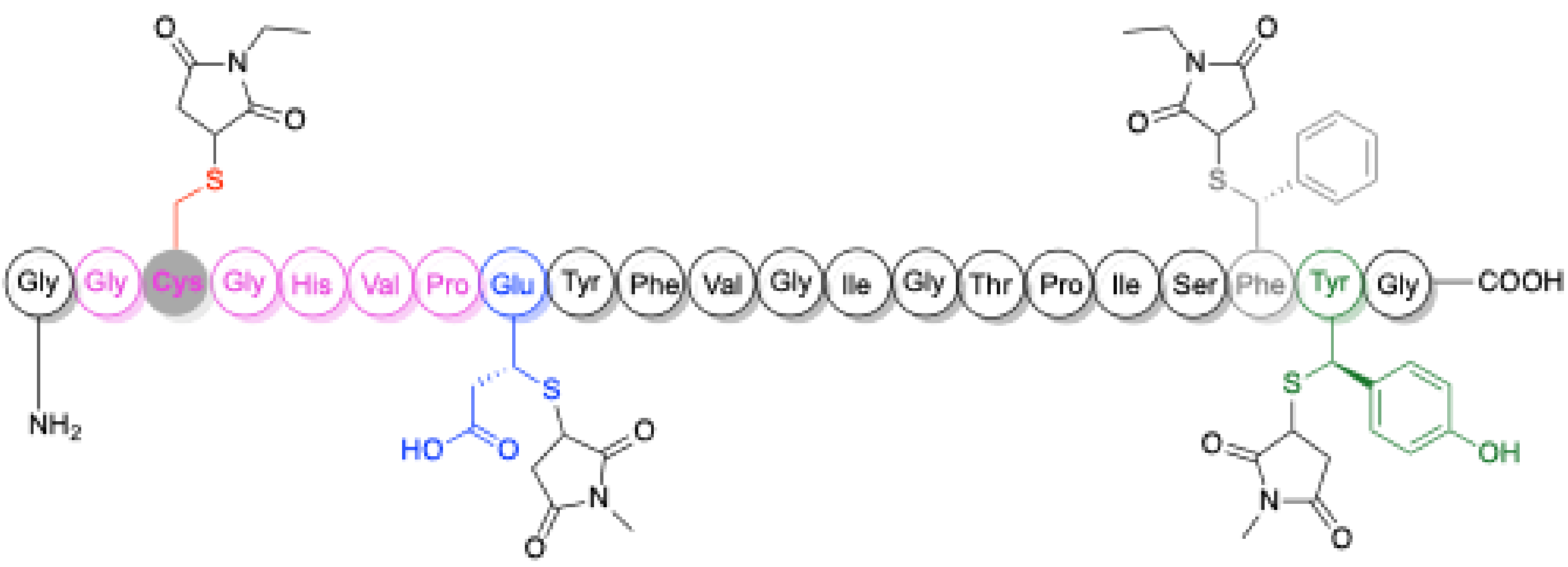

S10

Peptide **72** was subjected to alkylation according to general procedure for disulfide mapping analysis to give peptide **S10**. LCMS/MS sequenced the peptide to give y_2-y_{15} ions and b_5-b_{19} ions. LCMS (ESI–TOF) $[M+2H]^{2+}$ calc'd for $C_{123}H_{167}N_{27}O_{36}S_4$ 1363.0469, found 1363.0460.

13.2 MS/MS of partially reduced Nem alkylated MccJ25 analog (S9)

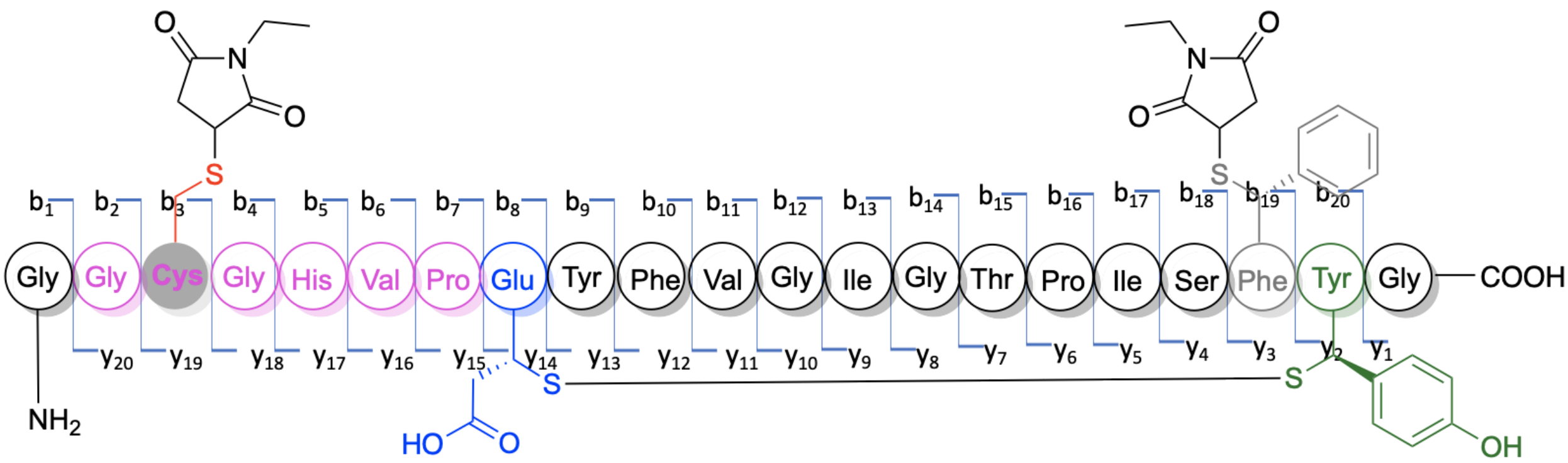

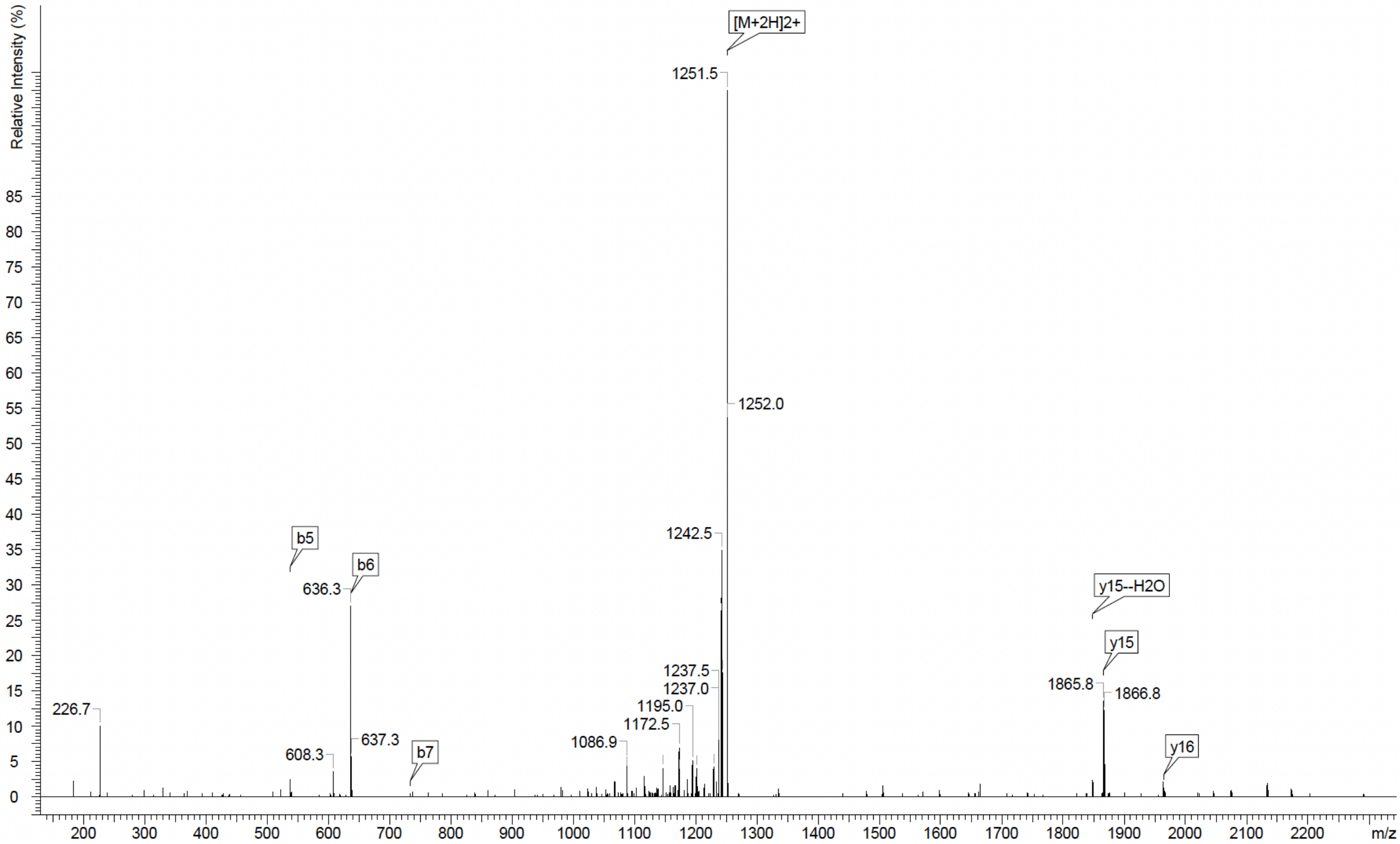

MS/MS of Nem alkylated peptide **S9**

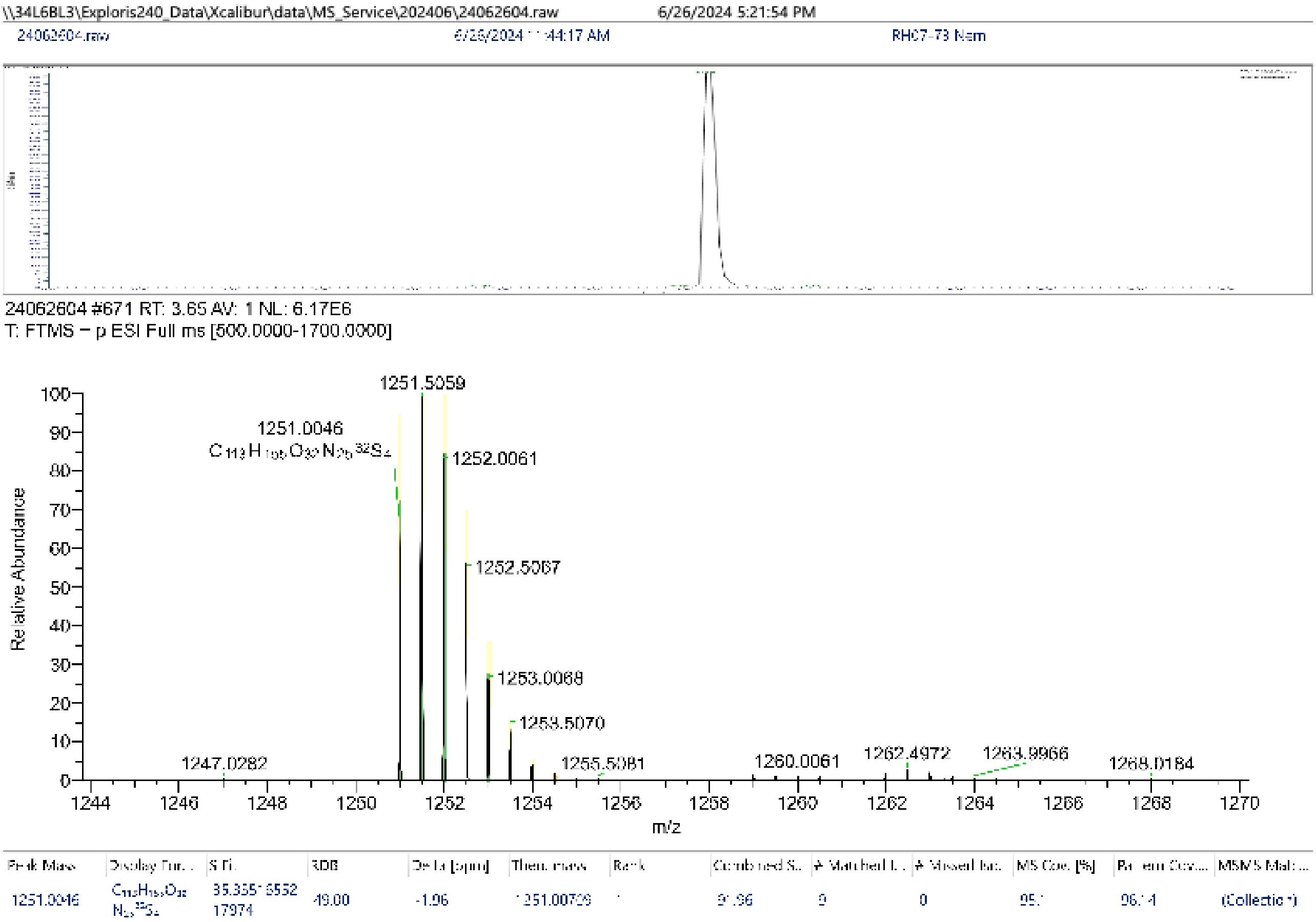

LCMS (ESI–TOF) of Nem alkylated peptide **S9**

13.3 MS/MS of Nem/Nmm alkylated MccJ25 analog (S10)

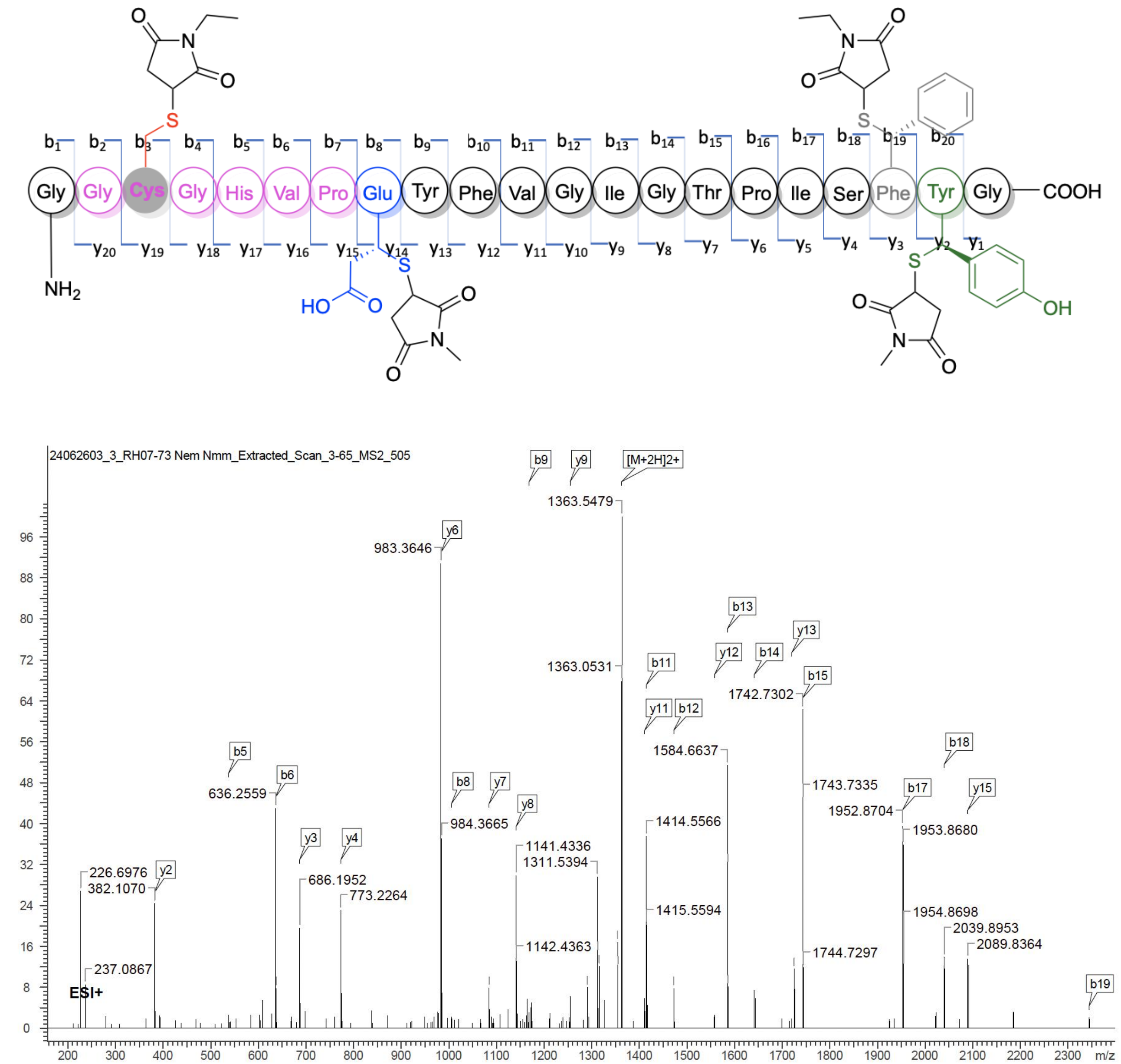

MS/MS of Nem and Nmm alkylated peptide **S10**

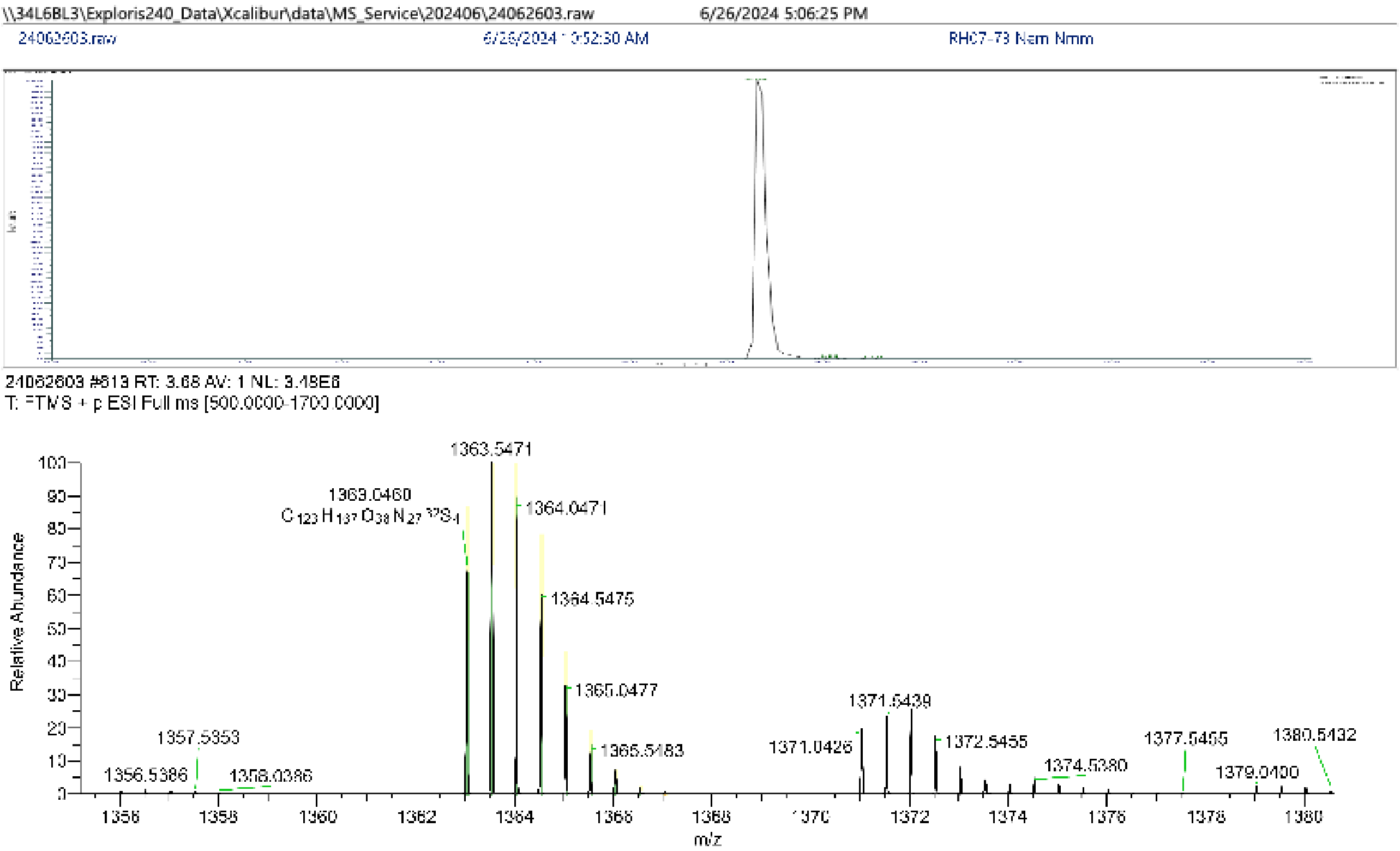

Peak Mass	Display For...	S Fit	RDB	Delta [ppm]	Theo. mass	Rank	Combined S...	# Matched I...	# Missed Iso.	MS Cov. [%]	Pattern Cov...	MSMS Matc...
1363.0460	$C_{123}H_{137}O_{38}N_{27}{}^{32}S_{4}$	58.043971698 1562	54.00	-0.68	1363.04654	1	97.51	9	0	99.7	99.85	(Collection)

LCMS (ESI–TOF) of Nem alkylated peptide **S10**

14) High Temperature ^{1}H NMR (400 MHz, DMSO-d_6) of Rotamer 52

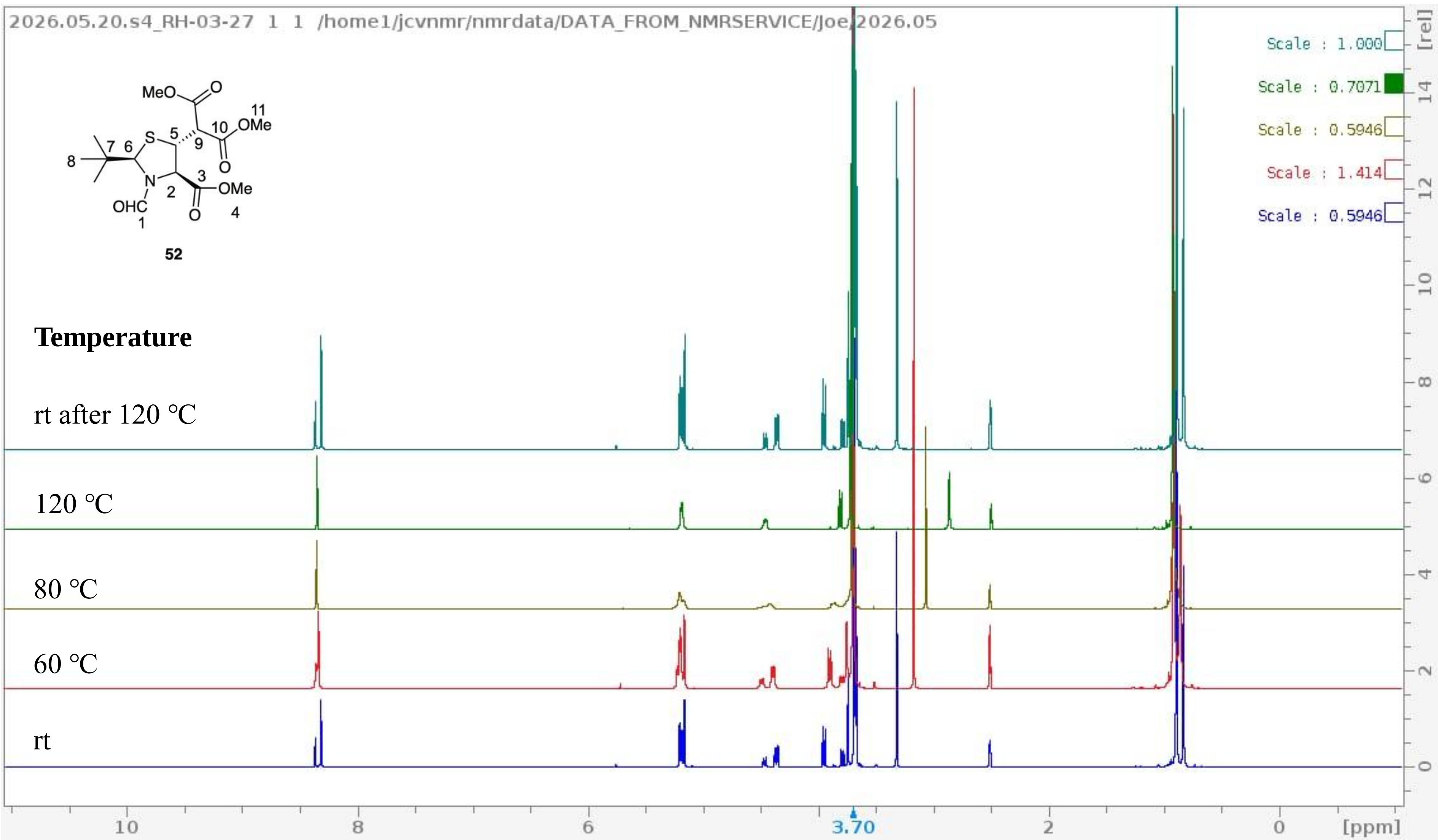

15) References

(1) Q. Wan, S.J. Danishefsky ‘‘Free-radical-based, specific desulfurization of cysteine: A powerful advance in the synthesis of polypeptides and glycopolypeptides.’’ *Angew. Chem. Int. Ed.* **2007**, *46*, 9248–9252.

(2) H. Liu, M. A. Boudreau, J. Zheng, R. M. Whittal, P. Austin, C. D. Roskelley, M. Roberge, R. J. Andersen, J. C. Vederas, “Chemical Synthesis and Biological Activity of the Neopetrosiamides and Their Analogues: Revision of Disulfide Bond Connectivity.” *J. Am. Chem. Soc.* **2010**, *132*, 1486–1487.

(3) H. Yin, M. Zheng, H. Chen, S. Wang, Q. Zhou, Q. Zhang, P. Wang, ‘‘Stereoselective and Divergent Construction of β-Thiolated/Selenolated Amino Acids via Photoredox-Catalyzed Asymmetric Giese Reaction.‘‘ *J. Am. Chem. Soc.* **2020**, *142*, 14201–14209.

(4) K. M. Cergol, R. E. Thompson, L. R. Malins, P. Turner, R. J. Payne, ‘‘One-Pot Peptide Ligation-Desulfurization at Glutamate.‘‘ *Org. Lett.* **2014**, *16*, 290–293.

(5) D. Hymel, K. Tsuji, R. A. Grant, R. M. Chingle, D. L. Kunciw, M. B. Yaffe, T. R. Burke, “Design and Synthesis of a New Orthogonally Protected Glutamic Acid Analog and Its Use in the Preparation of High Affinity Polo-like Kinase 1 Polo-Box Domain – Binding Peptide Macrocycles.” *Org. Biomol. Chem.* **2021**, *19*, 7843–7854.

(6) K. J. Rosengren, R. J. Clark, N. L. Daly, U. Göransson, A. Jones, D. J. Craik, ‘‘Microcin J25 Has a Threaded Sidechain-to-Backbone Ring Structure and Not a Head-to-Tail Cyclized Backbone.‘‘ *J. Am. Chem. Soc.* **2003**, *125*, 12464–12474.

(7) K. R. Buchholz, M. Reichelt, M. C. Johnson, S. J. Robinson, P. A. Smith, S. T. Rutherford, J. G. Quinn, “Potent Activity of Polymyxin B Is Associated with Long-Lived Super-Stoichiometric Accumulation Mediated by Weak-Affinity Binding to Lipid A.” *Nat. Commun. 2024 151* **2024**, *15*, 1–13.

16) NMR Spectra

OpenVnmrJ

Department of Chemistry, University of Alberta

Recorded on: u500, Jul 23 2022	Sweep Width(Hz): 6009.62	Acquisiton Time(s): 5	Relaxation Delay(s): 0.1
Pulse Sequence: PRESAT	Digital Res.(Hz/pt): 0.09	Hz per mm(Hz/mm): 25.04	Completed Scans 8

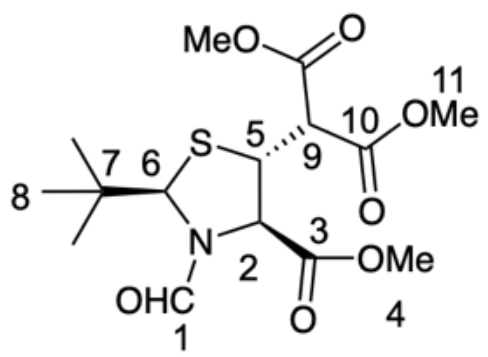

52

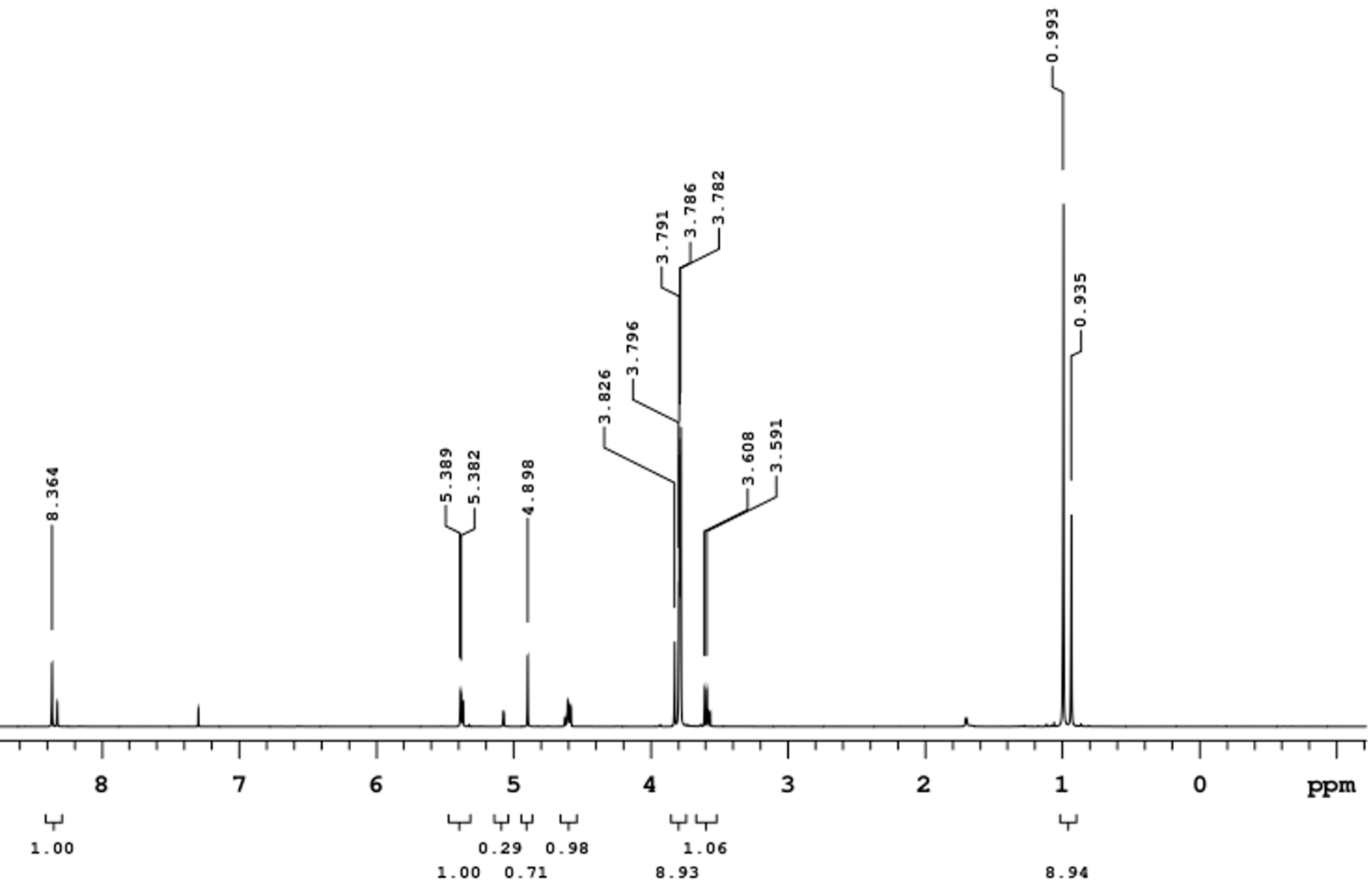

OpenVnmrJ

Department of Chemistry, University of Alberta

Recorded on: u500, Jul 23 2022 Sweep Width(Hz): 33783.8 Acquisiton Time(s): 1 Relaxation Delay(s): 1
Pulse Sequence: s2pul Digital Res.(Hz/pt): 0.26 Hz per mm(Hz/mm): 140.76 Completed Scans 128

Riley, RH03-27-1
125.685 MHz C13(H1) 1D in cdcl3 (ref. to CDCl3 @ 77.06 ppm)
temp 27.7 C -> actual temp = 27.0 C, colddual probe

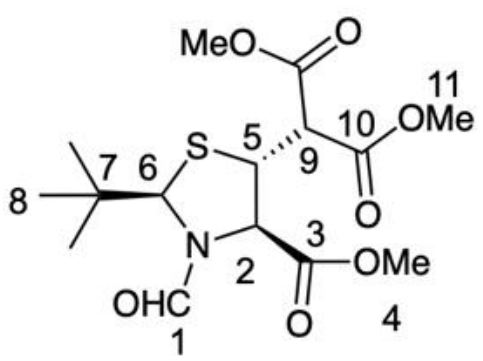

52

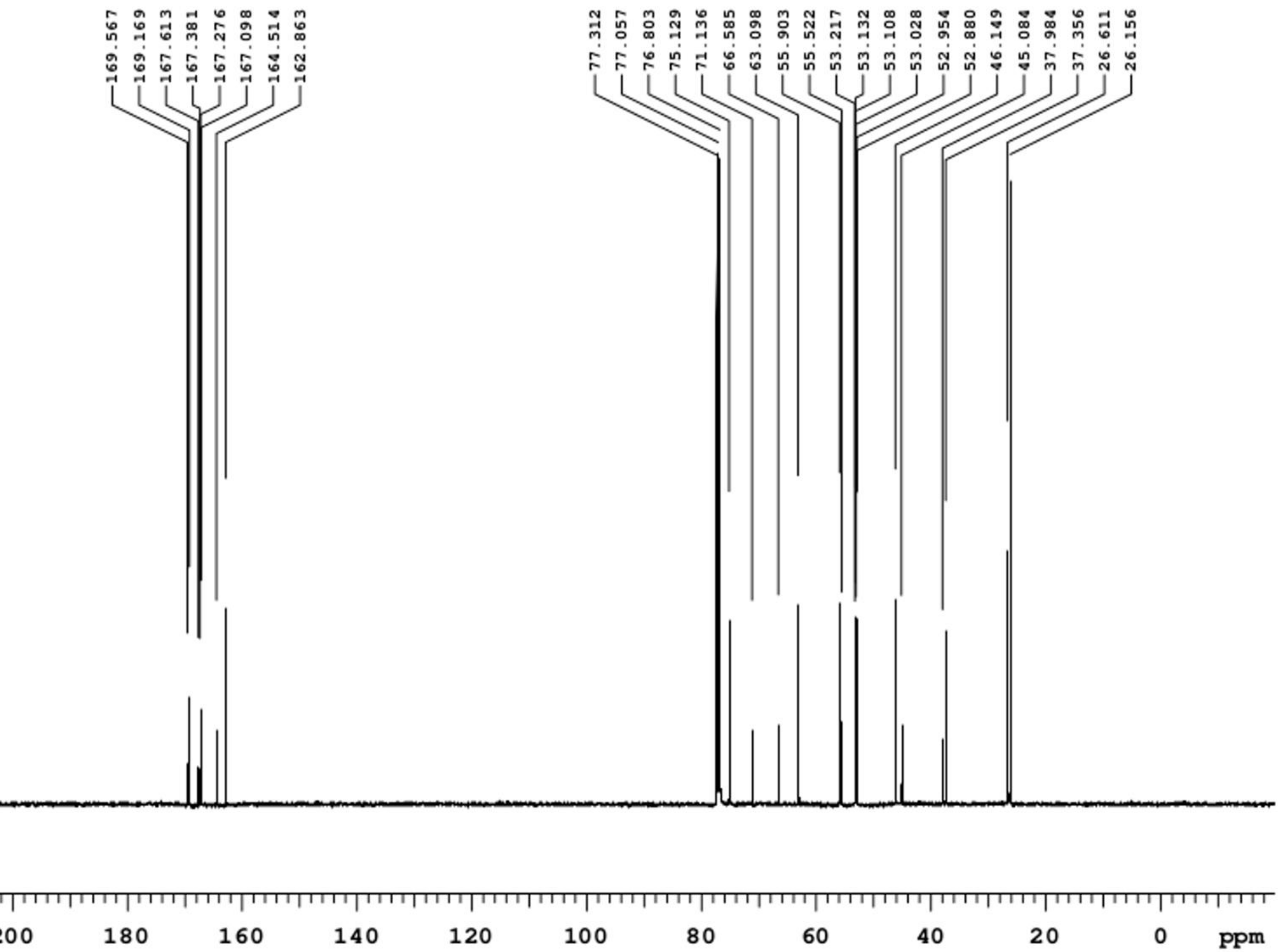

OpenVnmrJ

Department of Chemistry, University of Alberta

Recorded on: u500, Aug 10 2022 Sweep Width(Hz): 6009.62 Acquisiton Time(s): 5 Relaxation Delay(s): 0.1
Pulse Sequence: PRESAT Digital Res.(Hz/pt): 0.09 Hz per mm(Hz/mm): 25.04 Completed Scans 8

Riley, RH03-56_F17-58
499.787 MHz H1 1D in cdcl3 (ref. to CDCl3 @ 7.26 ppm)
temp 27.7 C -> actual temp = 27.0 C, colddual probe

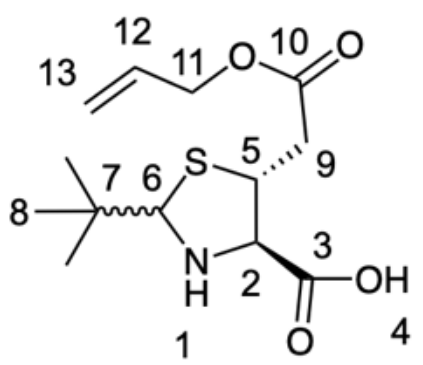

53

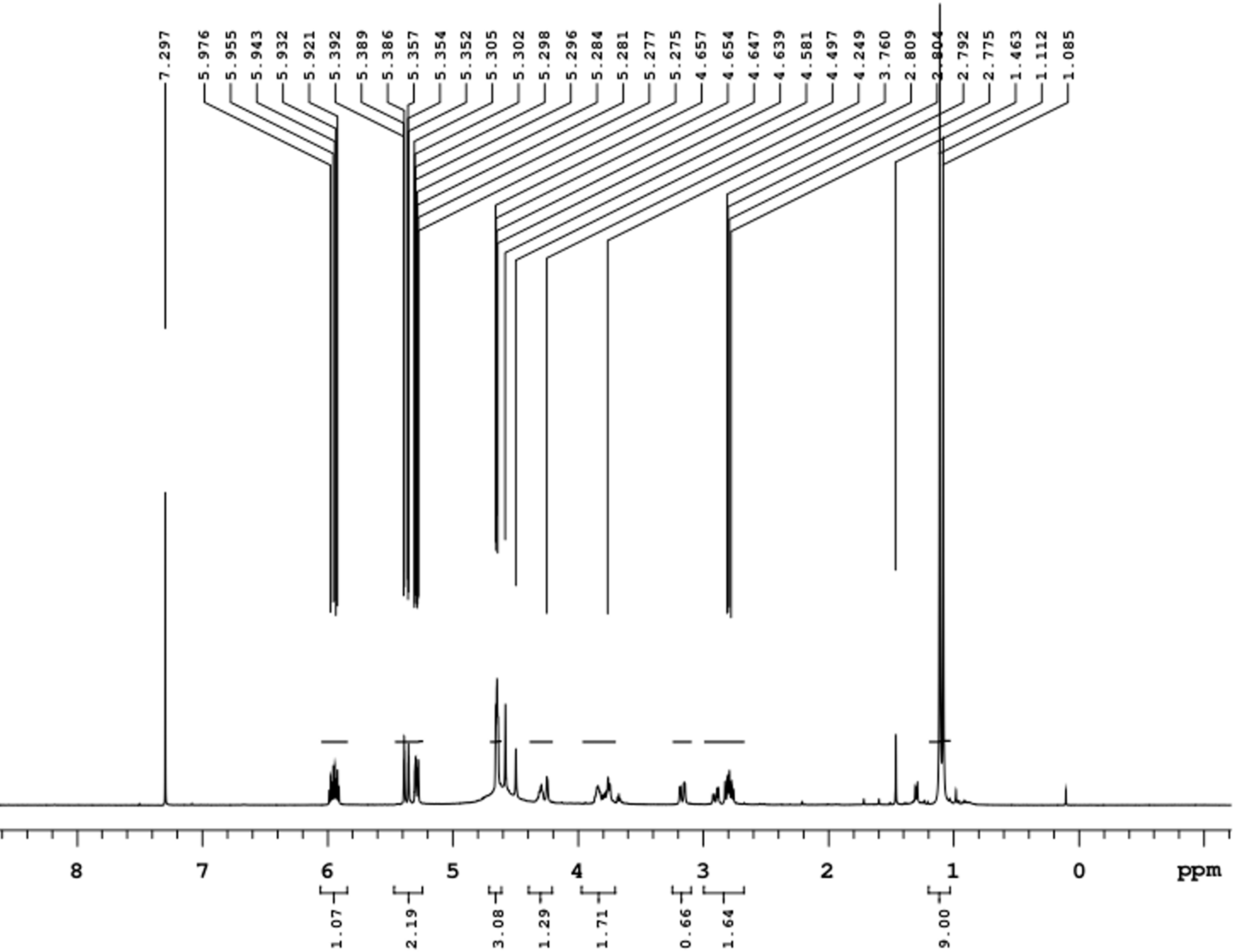

OpenVnmrJ

Department of Chemistry, University of Alberta

Recorded on: u500, Aug 10 2022 Sweep Width(Hz): 33783.8 Acquisiton Time(s): 1 Relaxation Delay(s): 1
Pulse Sequence: s2pul Digital Res.(Hz/pt): 0.26 Hz per mm(Hz/mm): 140.76 Completed Scans 128

Riley, RH03-56_F17-58
125.685 MHz C13{H1} 1D in cdcl3 (ref. to CDCl3 @ 77.06 ppm)
temp 27.7 C -> actual temp = 27.0 C, colddual probe

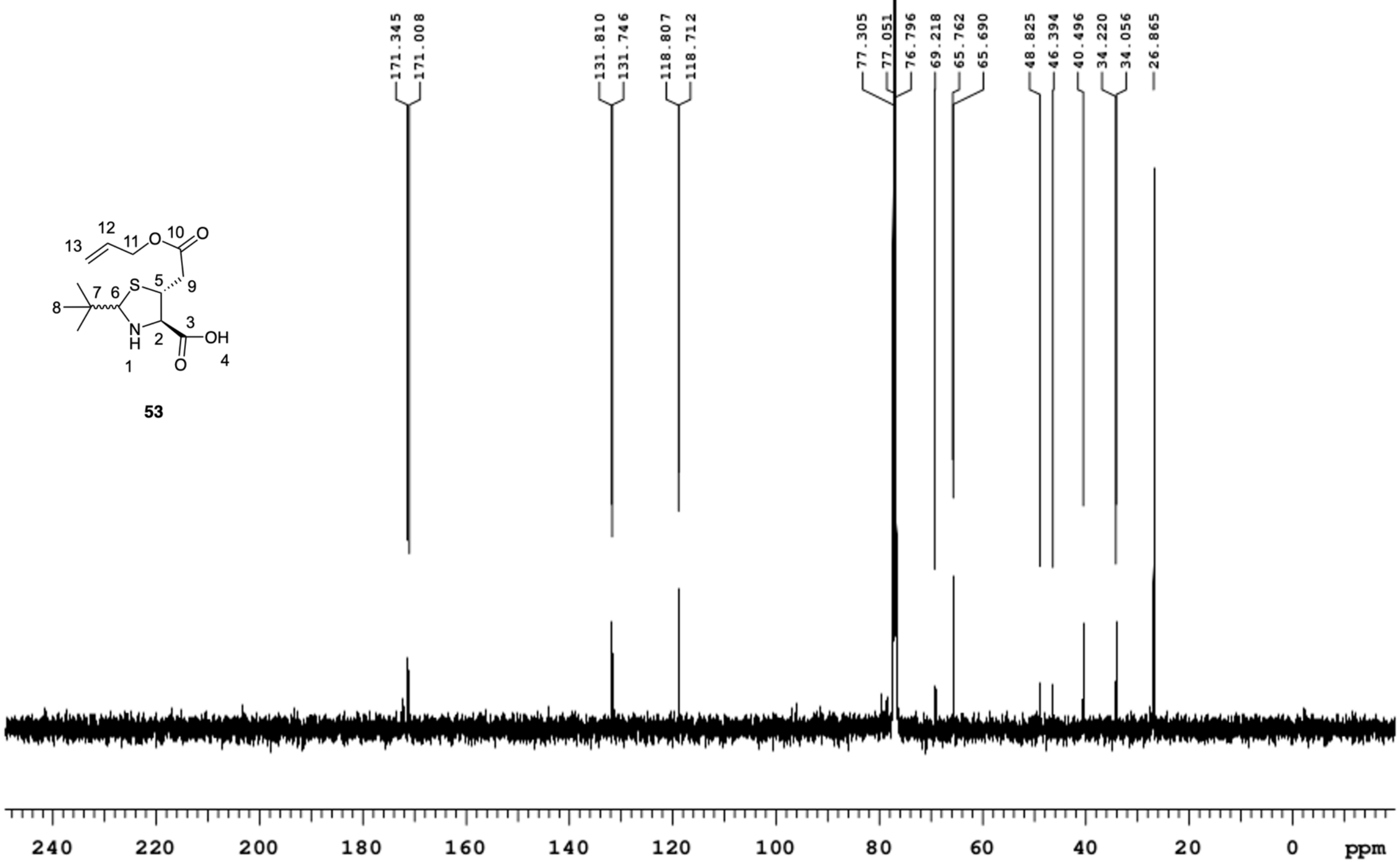

53

OpenVnmrJ

Department of Chemistry, University of Alberta

Recorded on: u500, Aug 11 2022	Sweep Width(Hz): 6009.62	Acquisiton Time(s): 5	Relaxation Delay(s): 0.1
Pulse Sequence: PRESAT	Digital Res.(Hz/pt): 0.09	Hz per mm(Hz/mm): 25.04	Completed Scans 8

Riley, RH03-59_F7-14
499.787 MHz H1 1D in cdcl3 (ref. to CDCl3 @ 7.26 ppm)
temp 27.7 C -> actual temp = 27.0 C, colddual probe

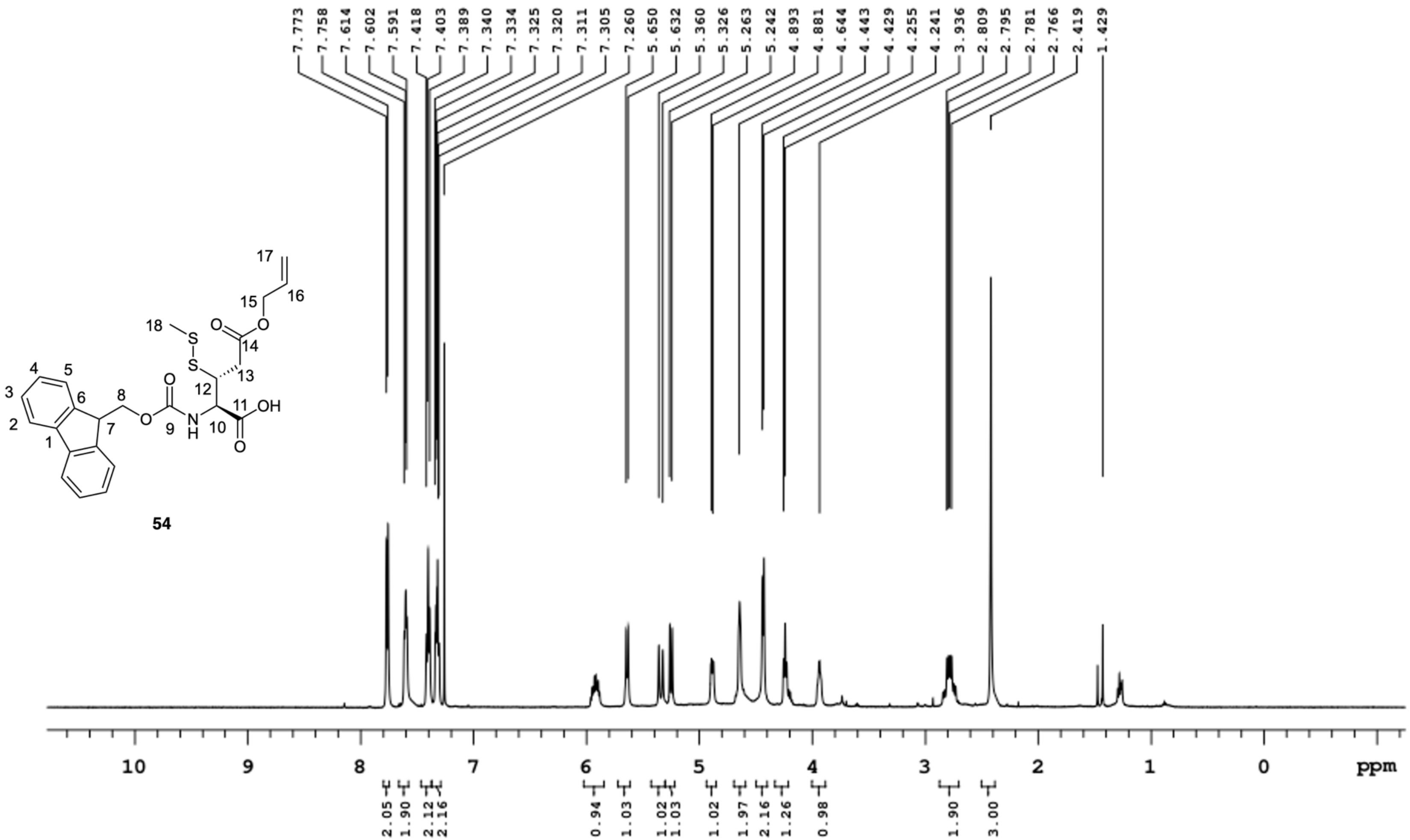

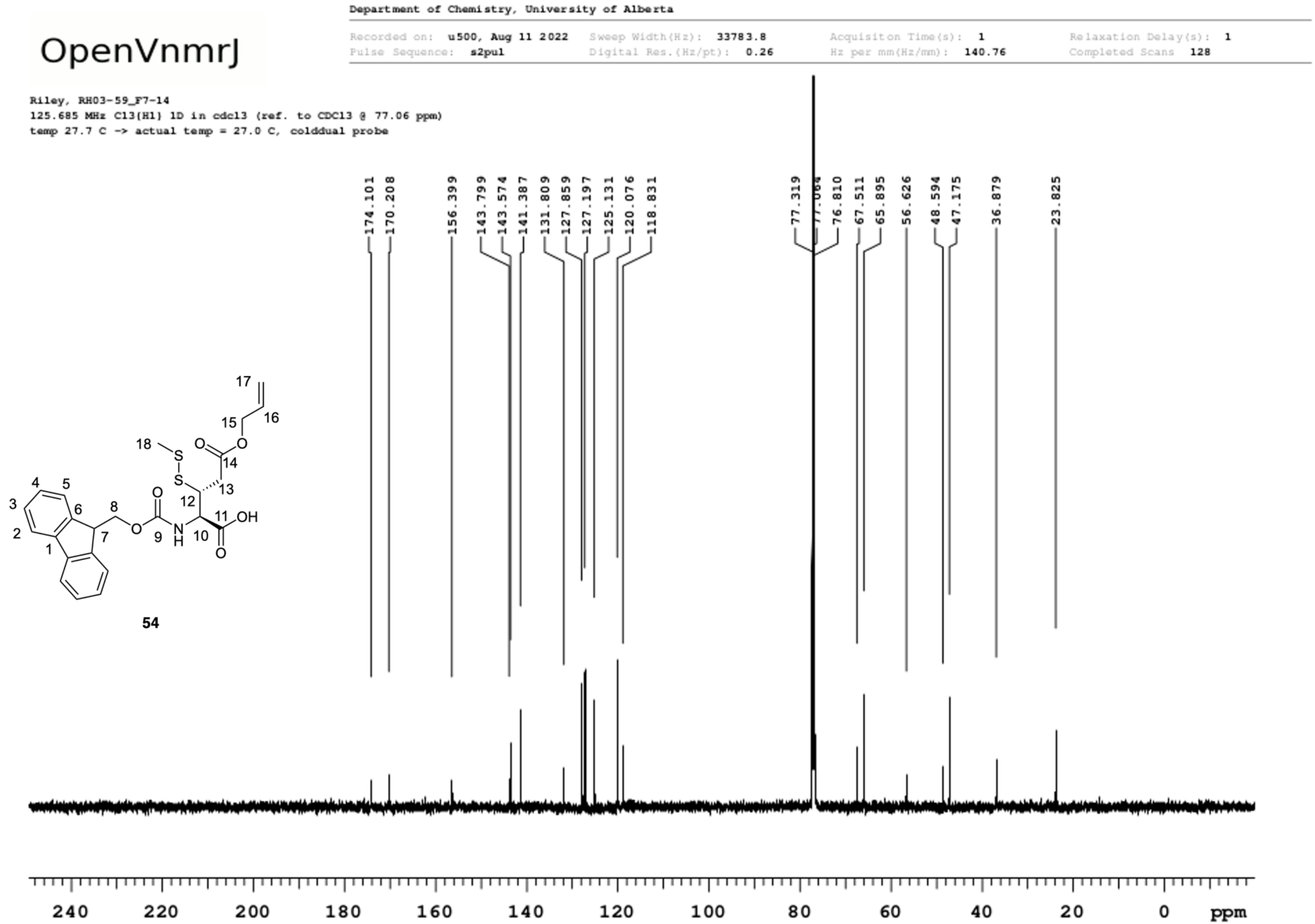

OpenVnmrJ
Department of Chemistry, University of Alberta
Recorded on: u500, Aug 11 2022
Sweep Width(Hz): 33783.8
Acquisiton Time(s): 1
Relaxation Delay(s): 1
Pulse Sequence: s2pul
Digital Res.(Hz/pt): 0.26
Hz per mm(Hz/mm): 140.76
Completed Scans 128
Riley, RH03-59_F7-14
125.685 MHz C13[H1] 1D in cdcl3 (ref. to CDCl3 @ 77.06 ppm)
temp 27.7 C -> actual temp = 27.0 C, colddual probe
174.101
170.208
156.399
143.799
143.574
141.387
131.809
127.859
127.197
125.131
120.076
118.831
77.319
76.810
67.511
65.895
56.626
48.594
47.175
36.879
23.825
240
220
200
180
160
140
120
100
80
60
40
20
0
ppm
17
16
15
18
O
O
S
S
14
13
12
11
OH
4
5
3
6
8
O
2
1
7
O
9
N
H
10
O

54

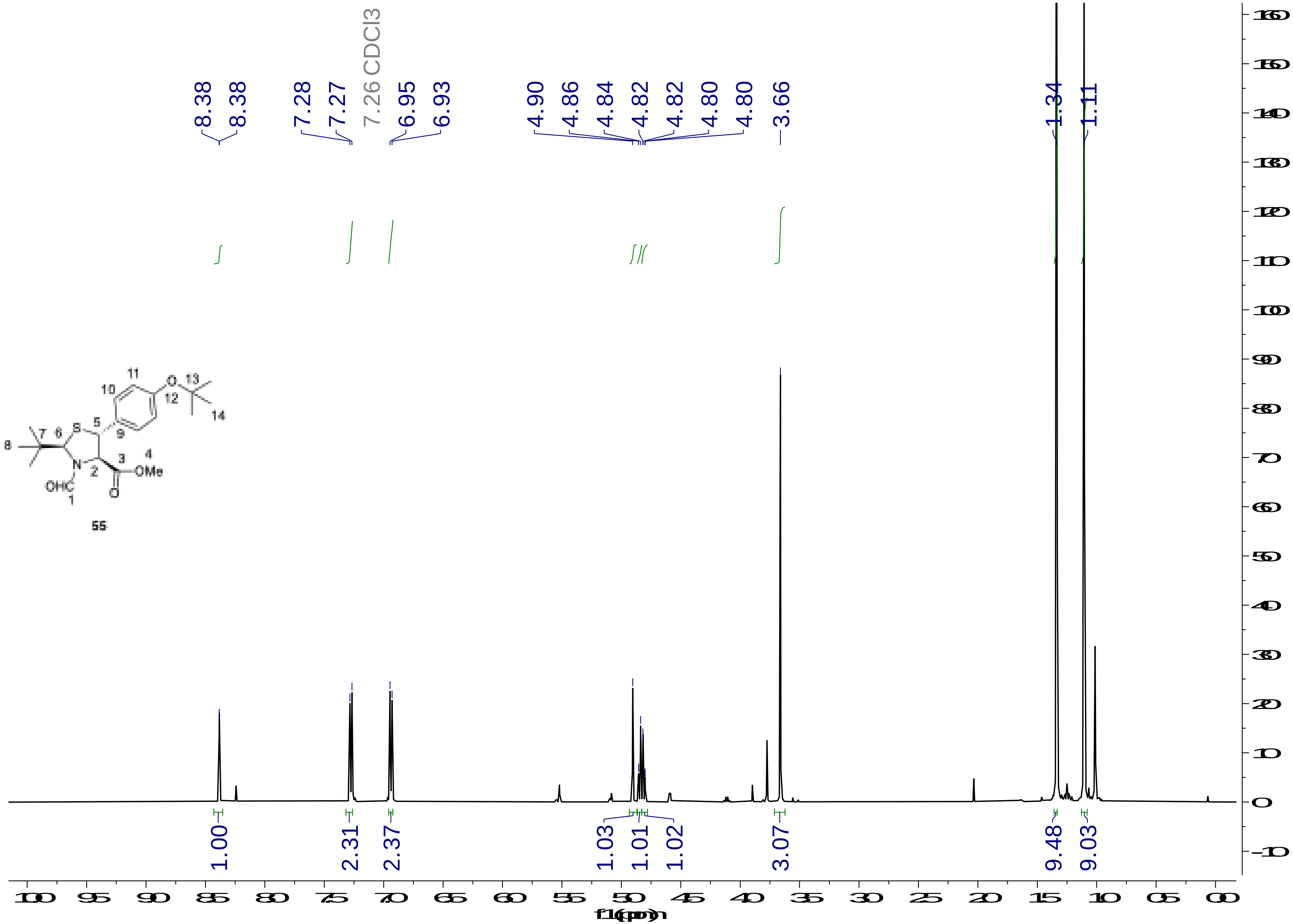
8.38
8.38
7.28
7.27
7.26 CDCl3
6.95
6.93
4.90
4.86
4.84
4.82
4.82
4.80
4.80
3.66
1.34
1.11
1.00
2.31
2.37
1.03
1.01
1.02
3.07
9.48
9.03
OHC
OMe
55
f1 (ppm)

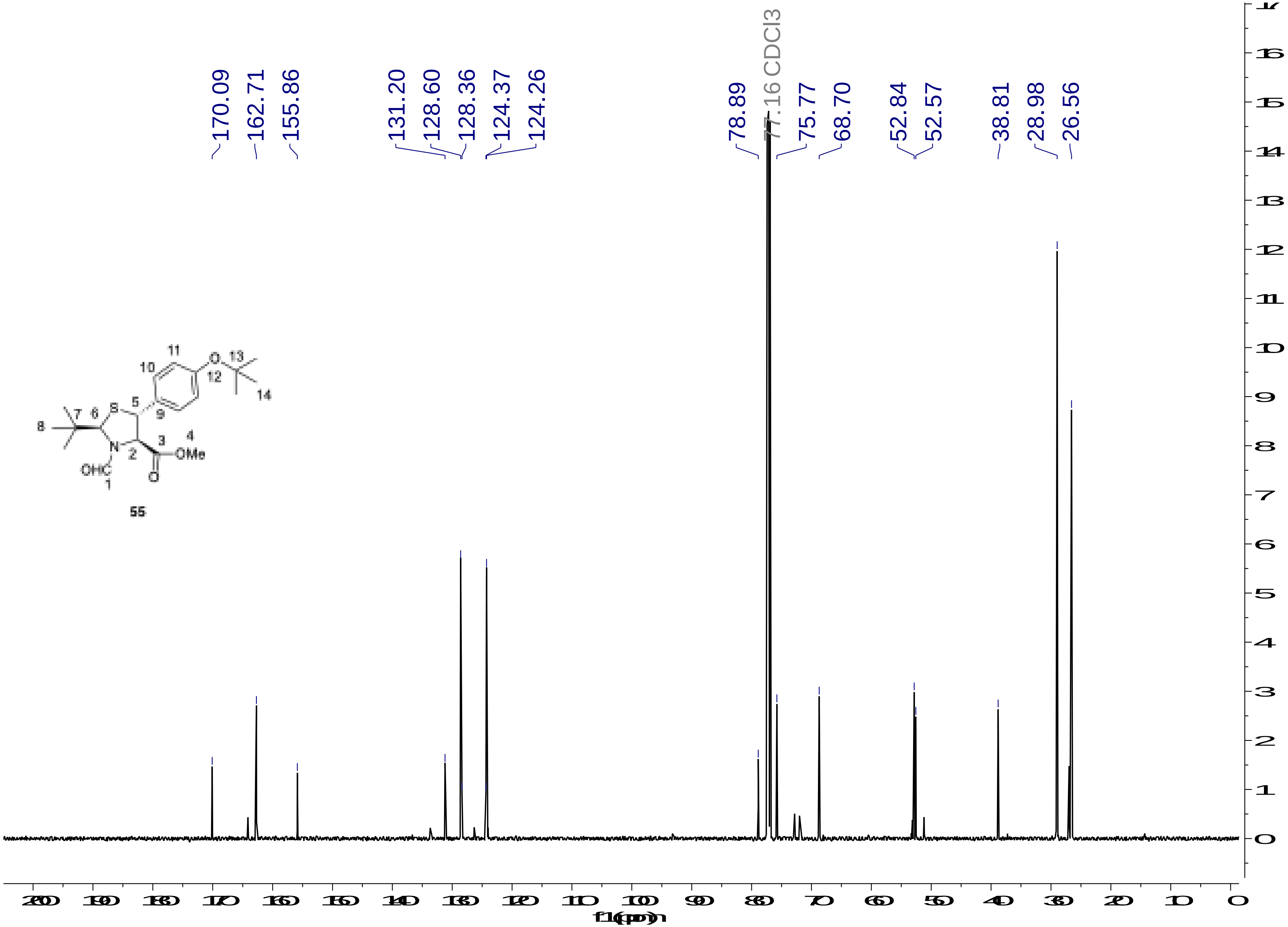
170.09
162.71
155.86
131.20
128.60
128.36
124.37
124.26
78.89
77.16 CDCl3
75.77
68.70
52.84
52.57
38.81
28.98
26.56
55
f1 (ppm)

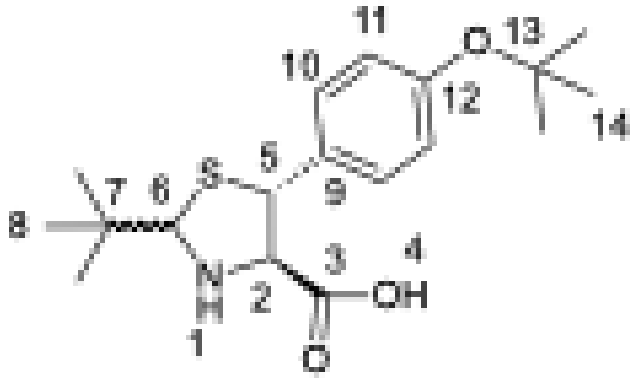

S
N
H
OH
O
O
1
2
3
4
5
6
7
8
9
10
11
12
13
14

56

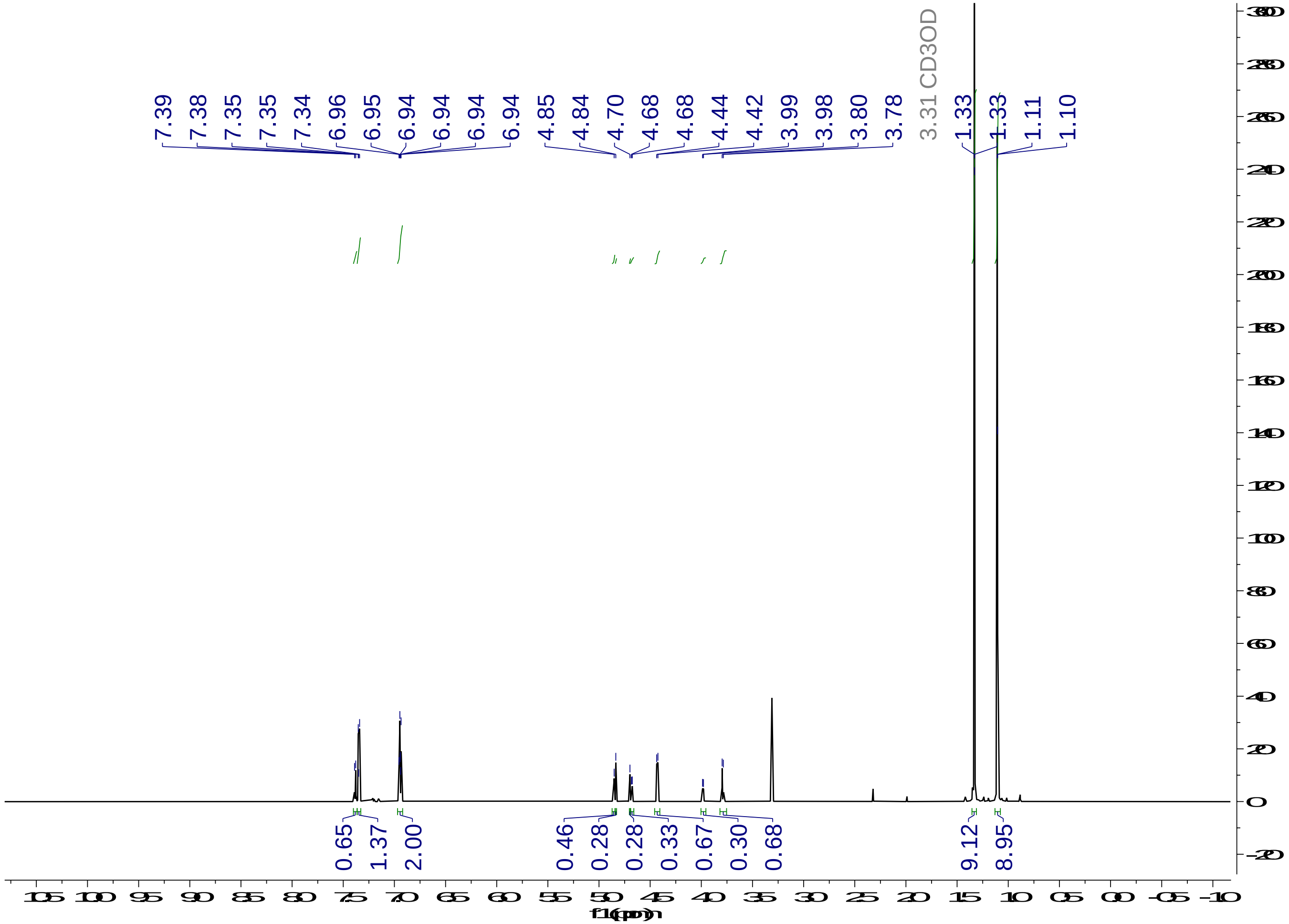

7.39
7.38
7.35
7.35
7.34
6.96
6.95
6.94
6.94
6.94
6.94
4.85
4.84
4.70
4.68
4.68
4.44
4.42
3.99
3.98
3.80
3.78
3.31 CD3OD
1.33
1.33
1.11
1.10
0.65
1.37
2.00
0.46
0.28
0.28
0.33
0.67
0.30
0.68
9.12
8.95
f1 (ppm)

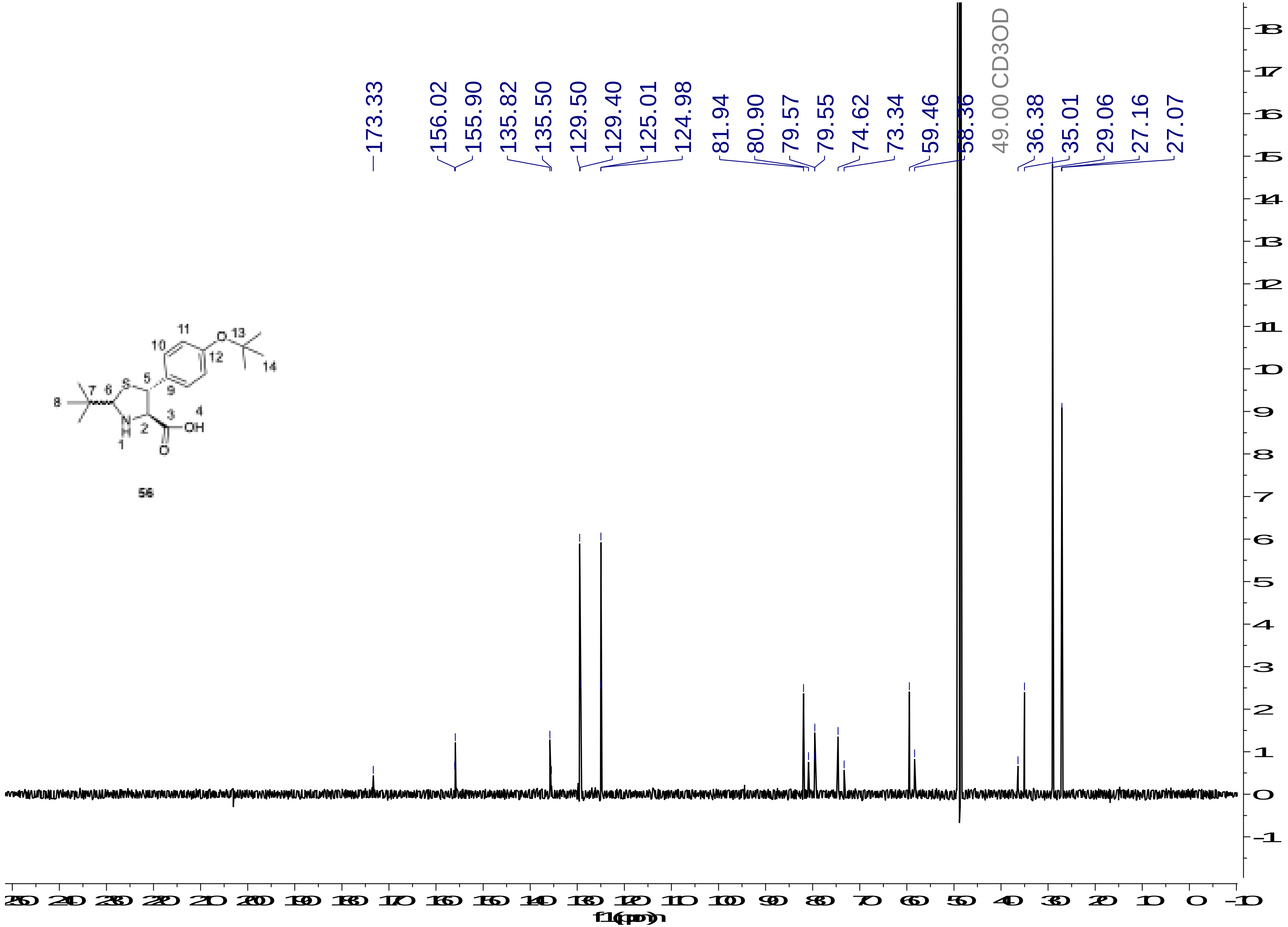
173.33
156.02
155.90
135.82
135.50
129.50
129.40
125.01
124.98
81.94
80.90
79.57
79.55
74.62
73.34
59.46
58.36
49.00 CD3OD
36.38
35.01
29.06
27.16
27.07
56
f1 (ppm)

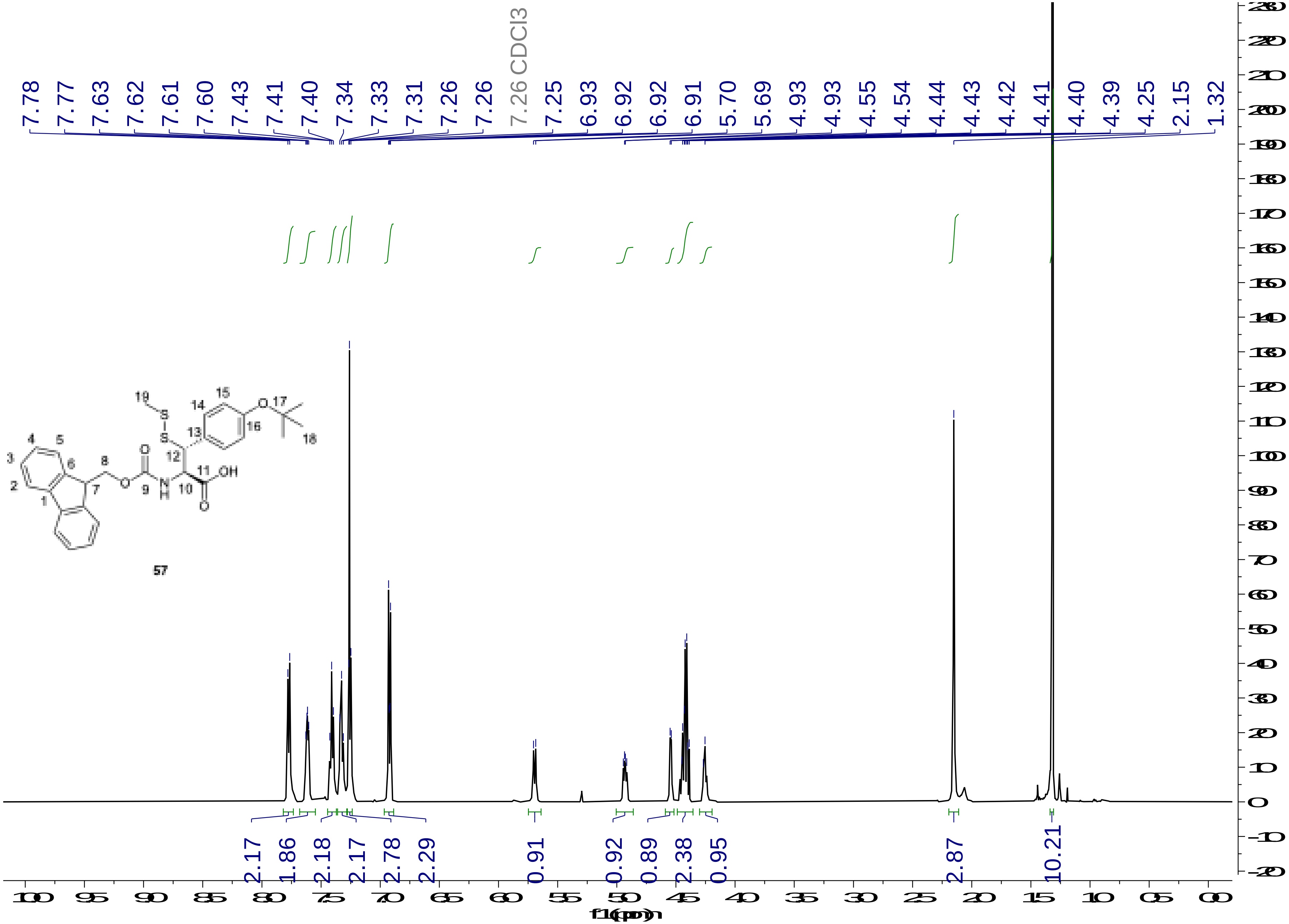
7.78
7.77
7.63
7.62
7.61
7.60
7.43
7.41
7.40
7.34
7.33
7.31
7.26
7.26
7.26 CDCl3
7.25
6.93
6.92
6.92
6.91
5.70
5.69
4.93
4.93
4.55
4.54
4.44
4.43
4.42
4.41
4.40
4.39
4.25
2.15
1.32
2.17
1.86
2.18
2.17
2.78
2.29
0.91
0.92
0.89
2.38
0.95
2.87
10.21
57
f1 (ppm)

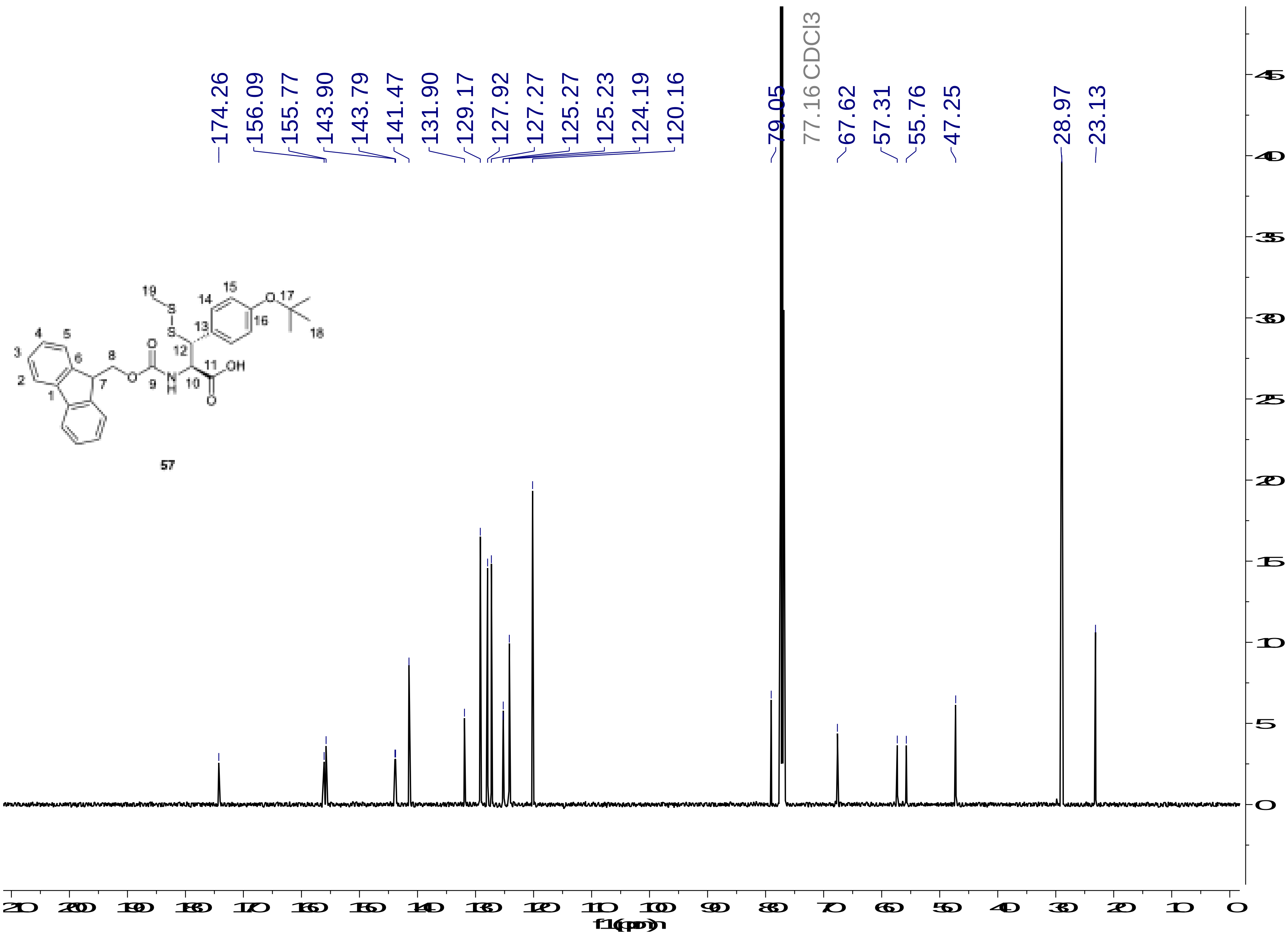
174.26
156.09
155.77
143.90
143.79
141.47
131.90
129.17
127.92
127.27
125.27
125.23
124.19
120.16
79.05
77.16 CDCl3
67.62
57.31
55.76
47.25
28.97
23.13
57
f1 (ppm)

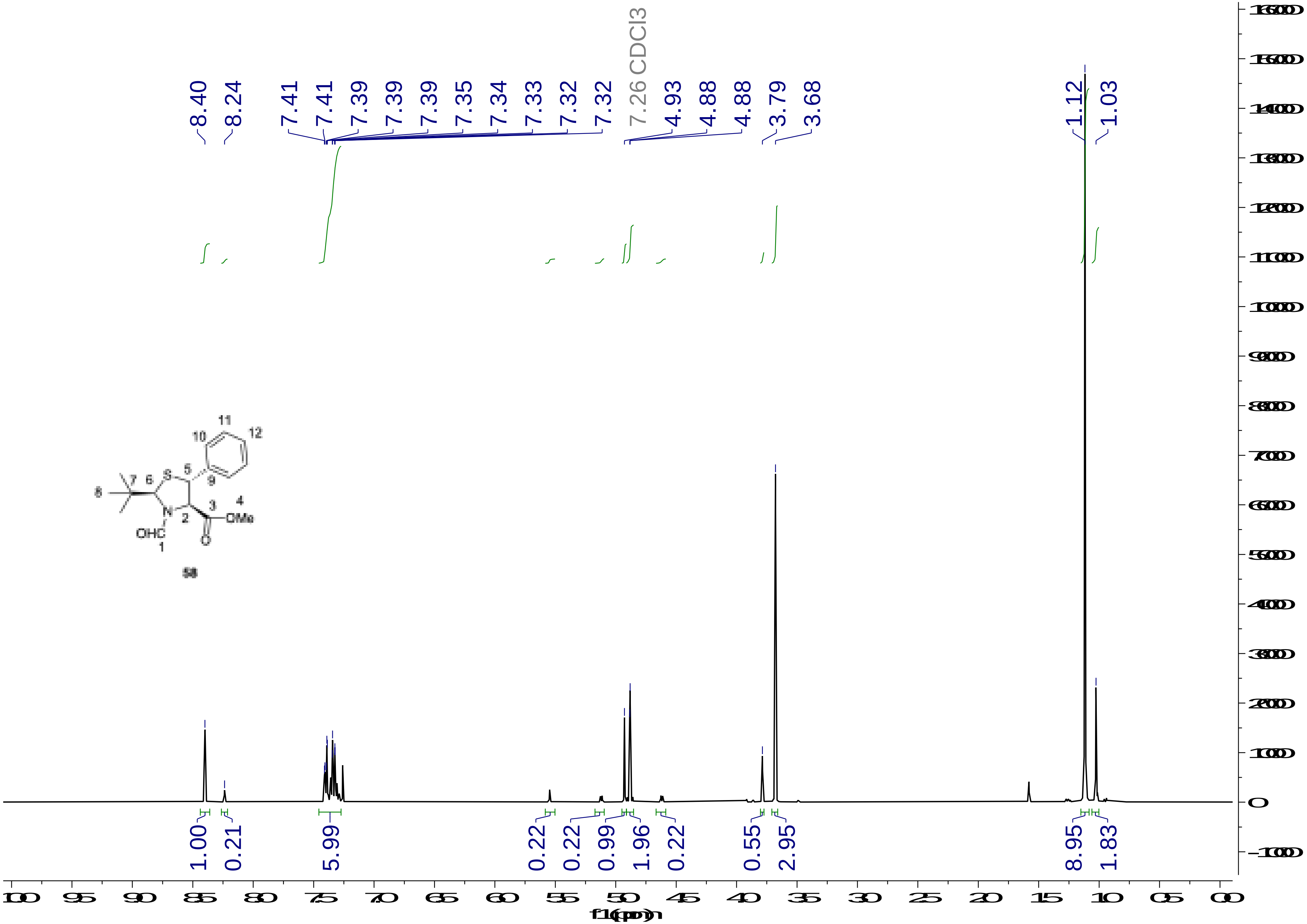
8.40
8.24
7.41
7.41
7.39
7.39
7.39
7.35
7.34
7.33
7.32
7.32
7.26 CDCl3
4.93
4.88
4.88
3.79
3.68
1.12
1.03
1.00
0.21
5.99
0.22
0.22
0.99
1.96
0.22
0.55
2.95
8.95
1.83
f1 (ppm)
OHC
OMe
58

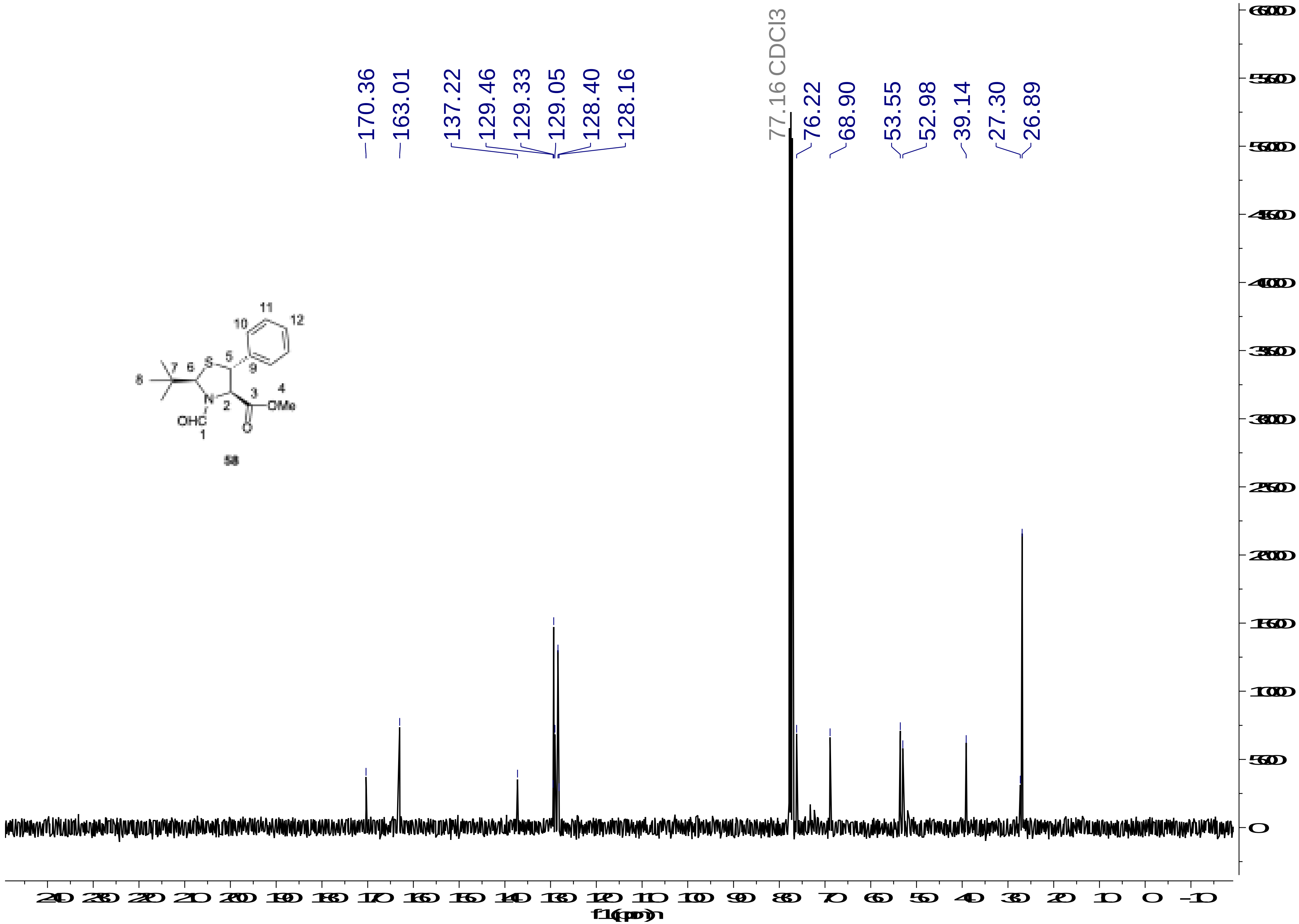
170.36
163.01
137.22
129.46
129.33
129.05
128.40
128.16
77.16 CDCl3
76.22
68.90
53.55
52.98
39.14
27.30
26.89
11
10
12
S
5
9
8
7
6
N
2
3
4
OMe
OHC
1
O
58
f1 (ppm)

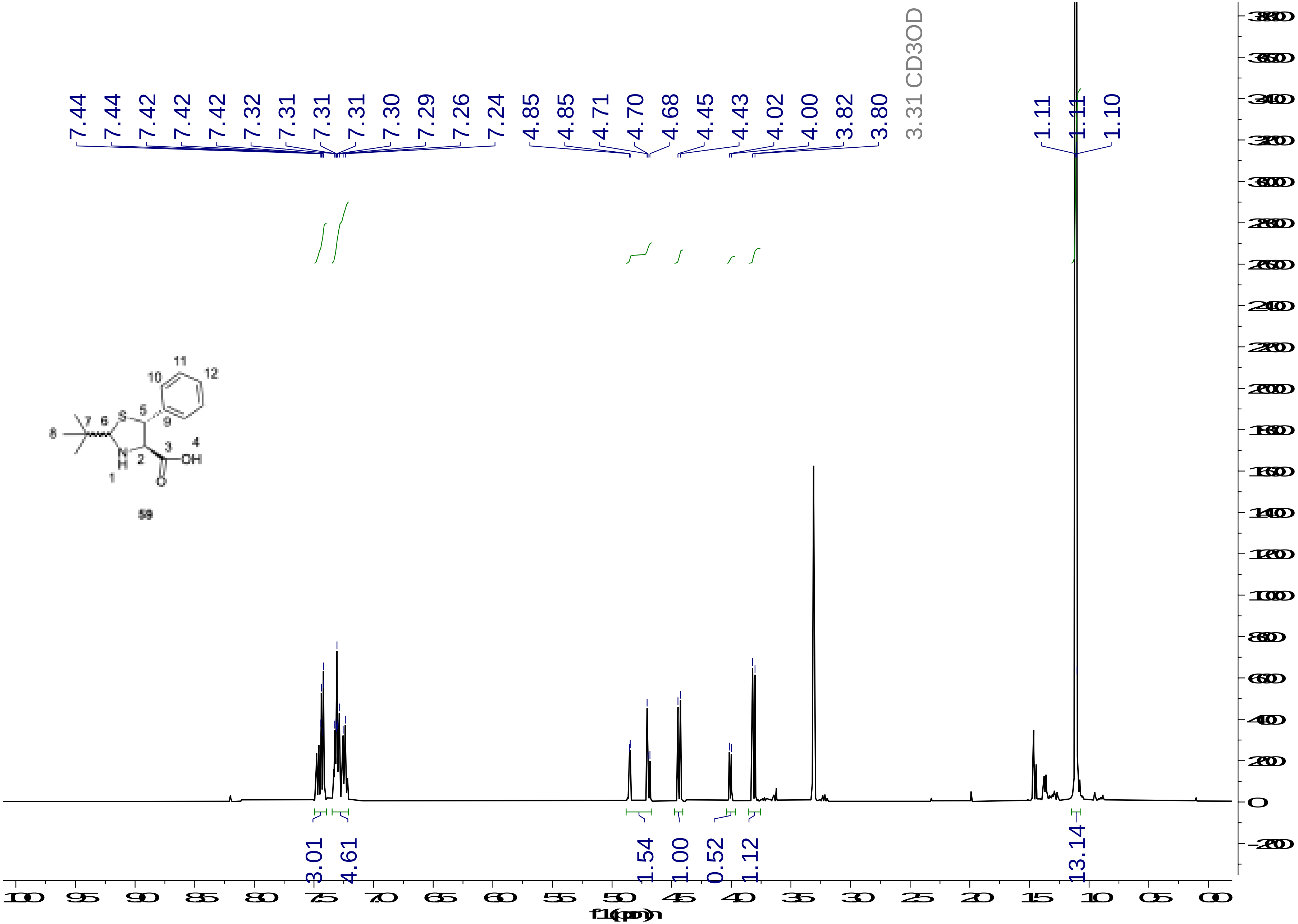
7.44
7.44
7.42
7.42
7.42
7.32
7.31
7.31
7.31
7.30
7.29
7.26
7.24
4.85
4.85
4.71
4.70
4.68
4.45
4.43
4.02
4.00
3.82
3.80
3.31 CD3OD
1.11
1.11
1.10
3.01
4.61
1.54
1.00
0.52
1.12
13.14
59
f1 (ppm)

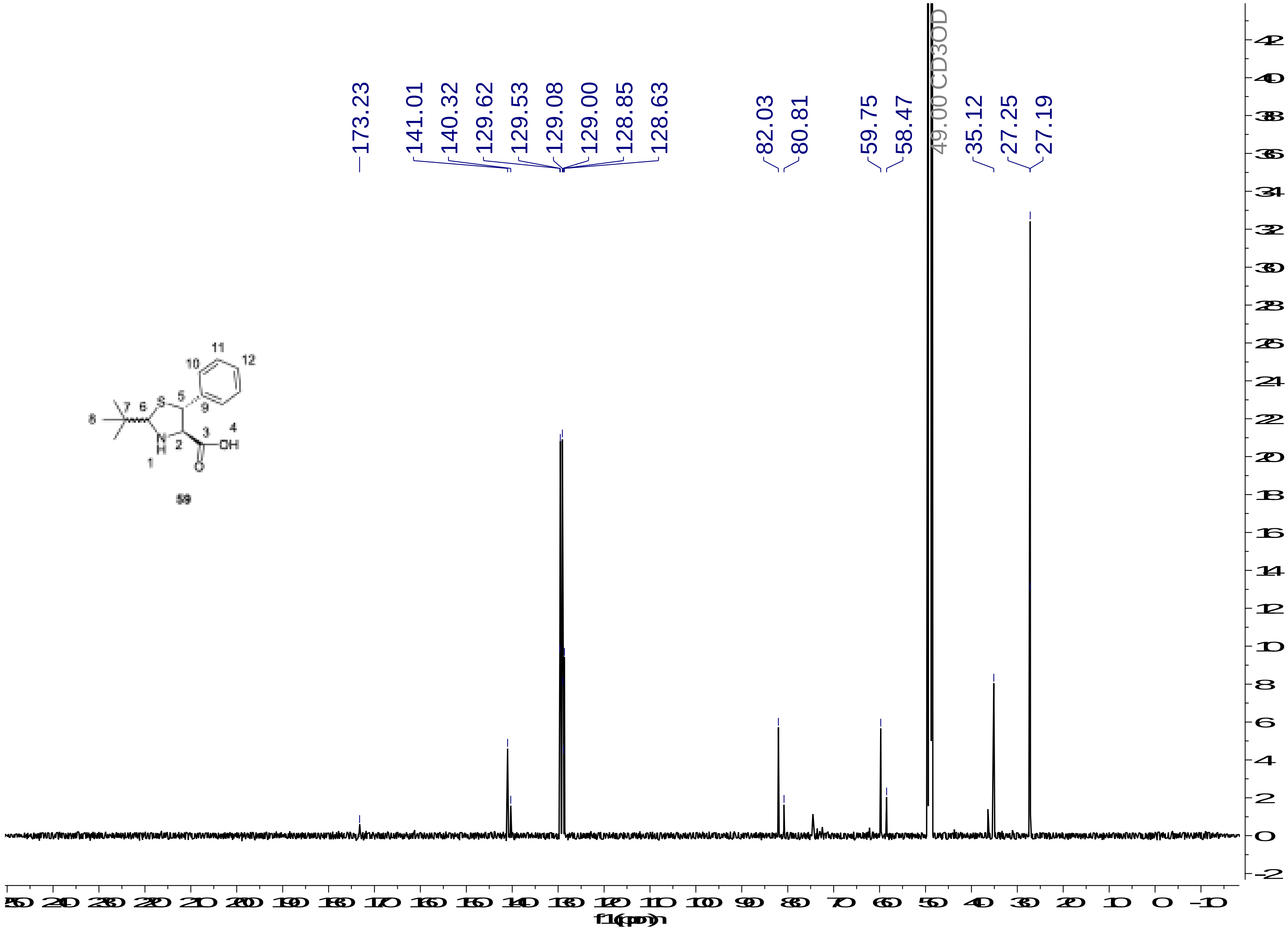
173.23
141.01
140.32
129.62
129.53
129.08
129.00
128.85
128.63
82.03
80.81
59.75
58.47
49.00 CD3OD
35.12
27.25
27.19
59
f1 (ppm)

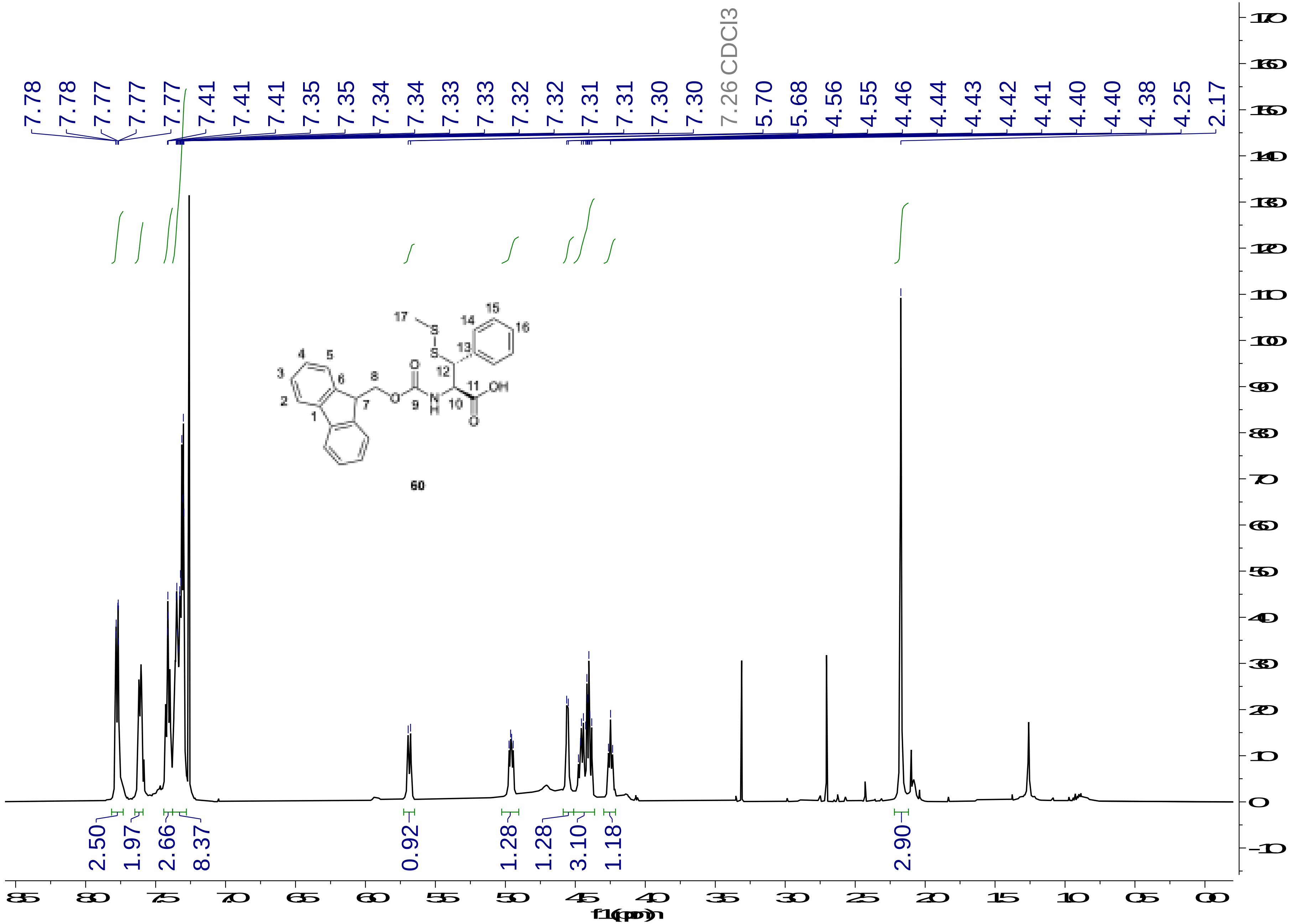
7.78
7.78
7.77
7.77
7.77
7.41
7.41
7.41
7.35
7.35
7.34
7.34
7.33
7.33
7.32
7.32
7.31
7.31
7.30
7.30
7.26 CDCl3
5.70
5.68
4.56
4.55
4.46
4.44
4.43
4.42
4.41
4.40
4.40
4.38
4.25
2.17
2.50
1.97
2.66
8.37
0.92
1.28
1.28
3.10
1.18
2.90
17
14
15
16
S
S
13
4
5
3
6
8
O
12
11
OH
2
7
O
9
N
H
10
1
O
60
8.5
8.0
7.5
7.0
6.5
6.0
5.5
5.0
4.5
4.0
3.5
3.0
2.5
2.0
1.5
1.0
0.5
0.0
f1 (ppm)
170
160
150
140
130
120
110
100
90
80
70
60
50
40
30
20
10
0
-10

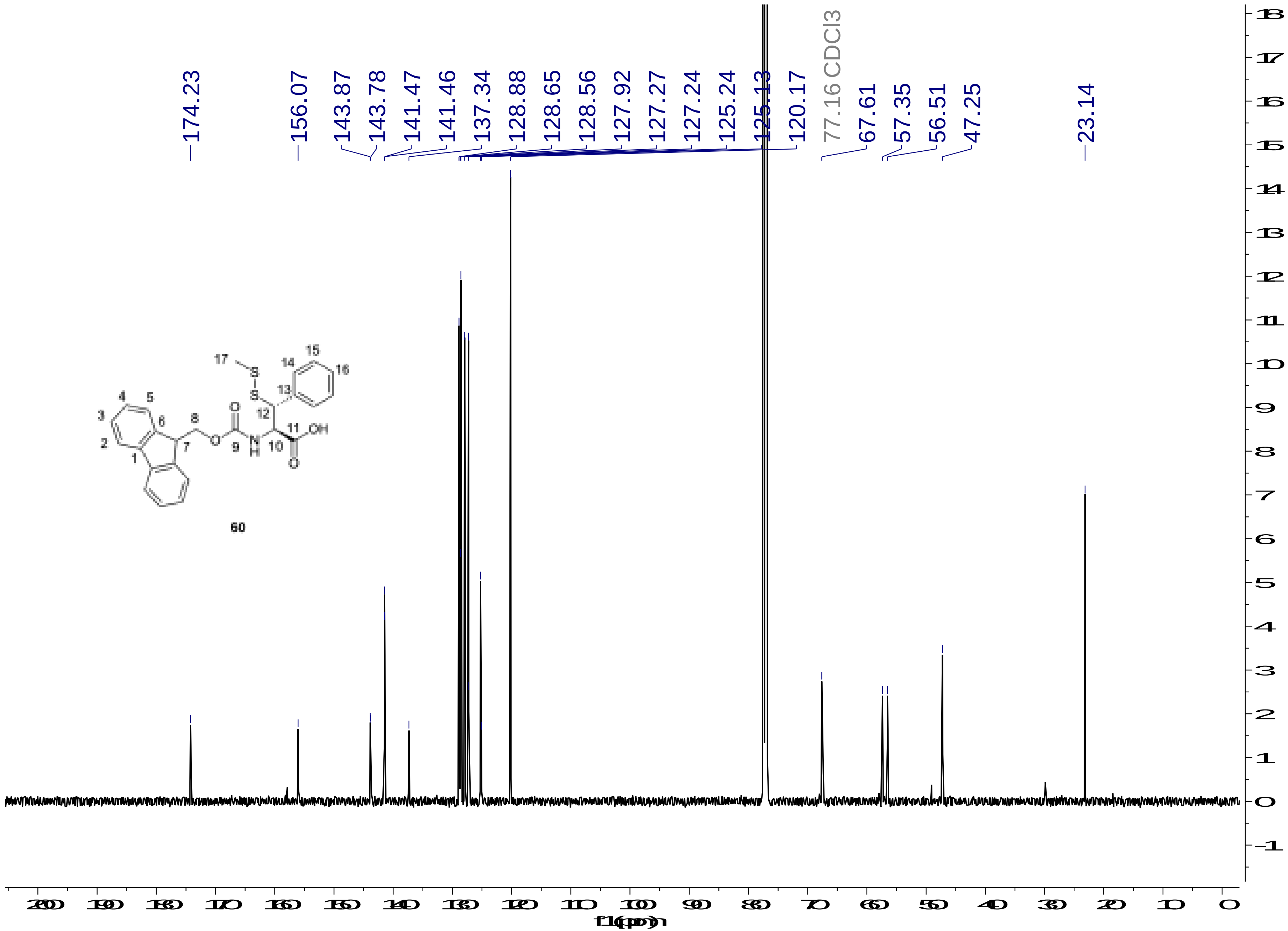
174.23
156.07
143.87
143.78
141.47
141.46
137.34
128.88
128.65
128.56
127.92
127.27
127.24
125.24
125.13
120.17
77.16 CDCl3
67.61
57.35
56.51
47.25
23.14
60
f1 (ppm)

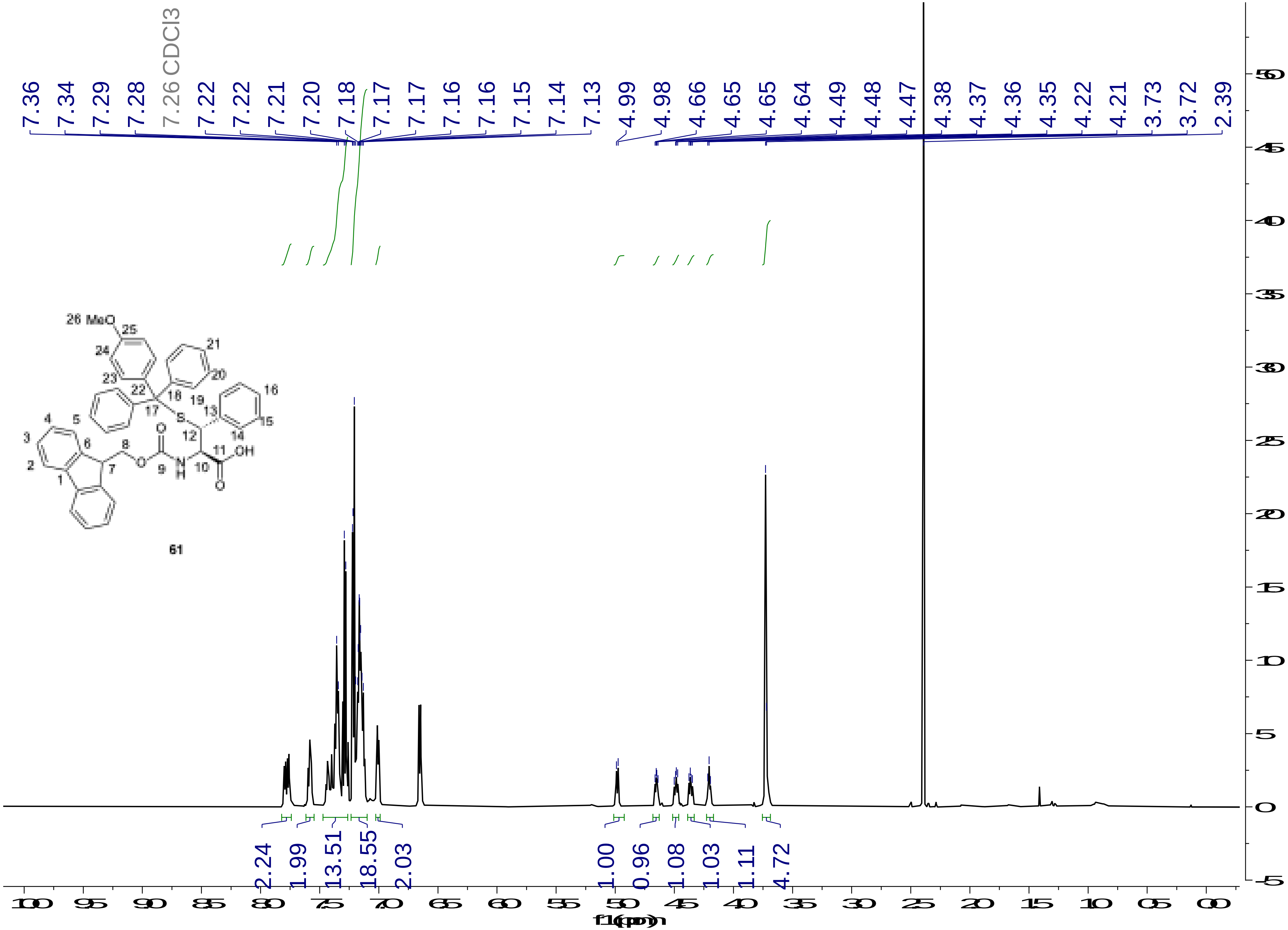

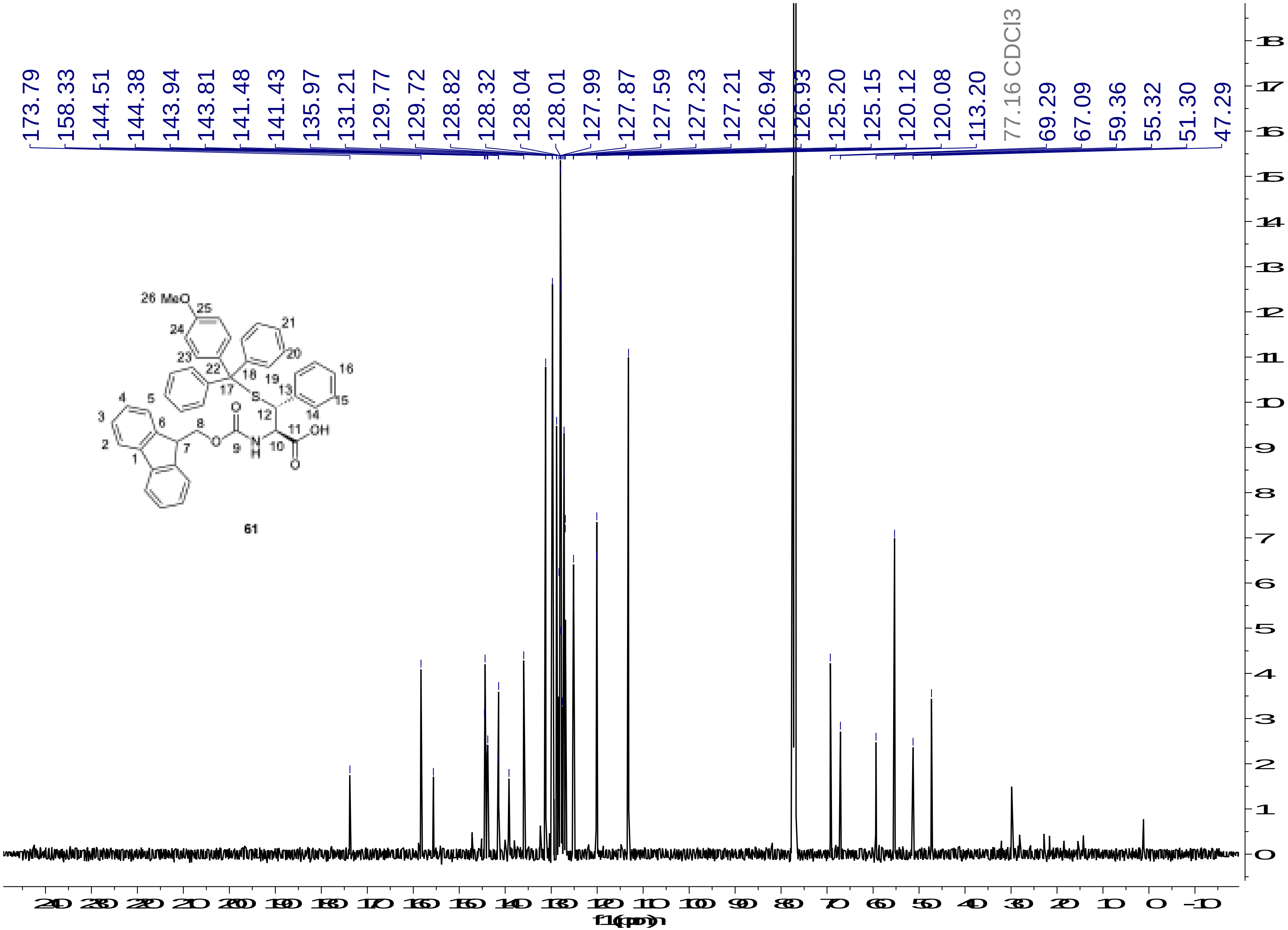
173.79
158.33
144.51
144.38
143.94
143.81
141.48
141.43
135.97
131.21
129.77
129.72
128.82
128.32
128.04
128.01
127.99
127.87
127.59
127.23
127.21
126.94
126.93
125.20
125.15
120.12
120.08
113.20
77.16 CDCl3
69.29
67.09
59.36
55.32
51.30
47.29
f1 (ppm)
26 MeO
OH
S
N
H
O

61

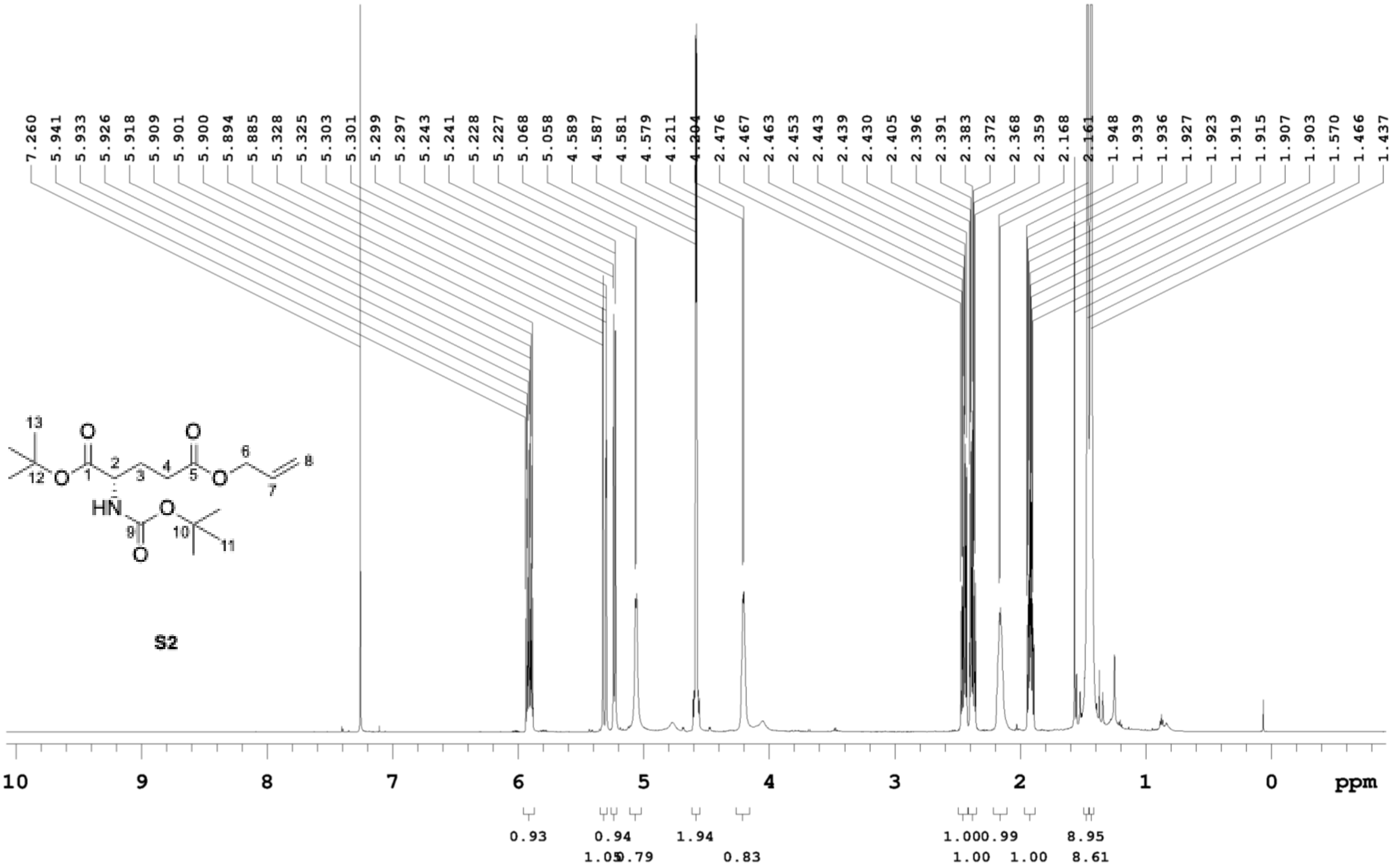
7.260
5.941
5.933
5.926
5.918
5.909
5.901
5.900
5.894
5.885
5.328
5.325
5.303
5.301
5.299
5.297
5.243
5.241
5.228
5.227
5.068
5.058
4.589
4.587
4.581
4.579
4.211
4.204
2.476
2.467
2.463
2.453
2.443
2.439
2.430
2.405
2.396
2.391
2.383
2.372
2.368
2.359
2.168
2.161
1.948
1.939
1.936
1.927
1.923
1.919
1.915
1.907
1.903
1.570
1.466
1.437
13
12
1
2
3
4
5
6
7
8
9
10
11
HN
O
S2
10 9 8 7 6 5 4 3 2 1 0 ppm
0.93
0.94
1.05
0.79
1.94
0.83
1.00
0.99
1.00
1.00
8.95
8.61

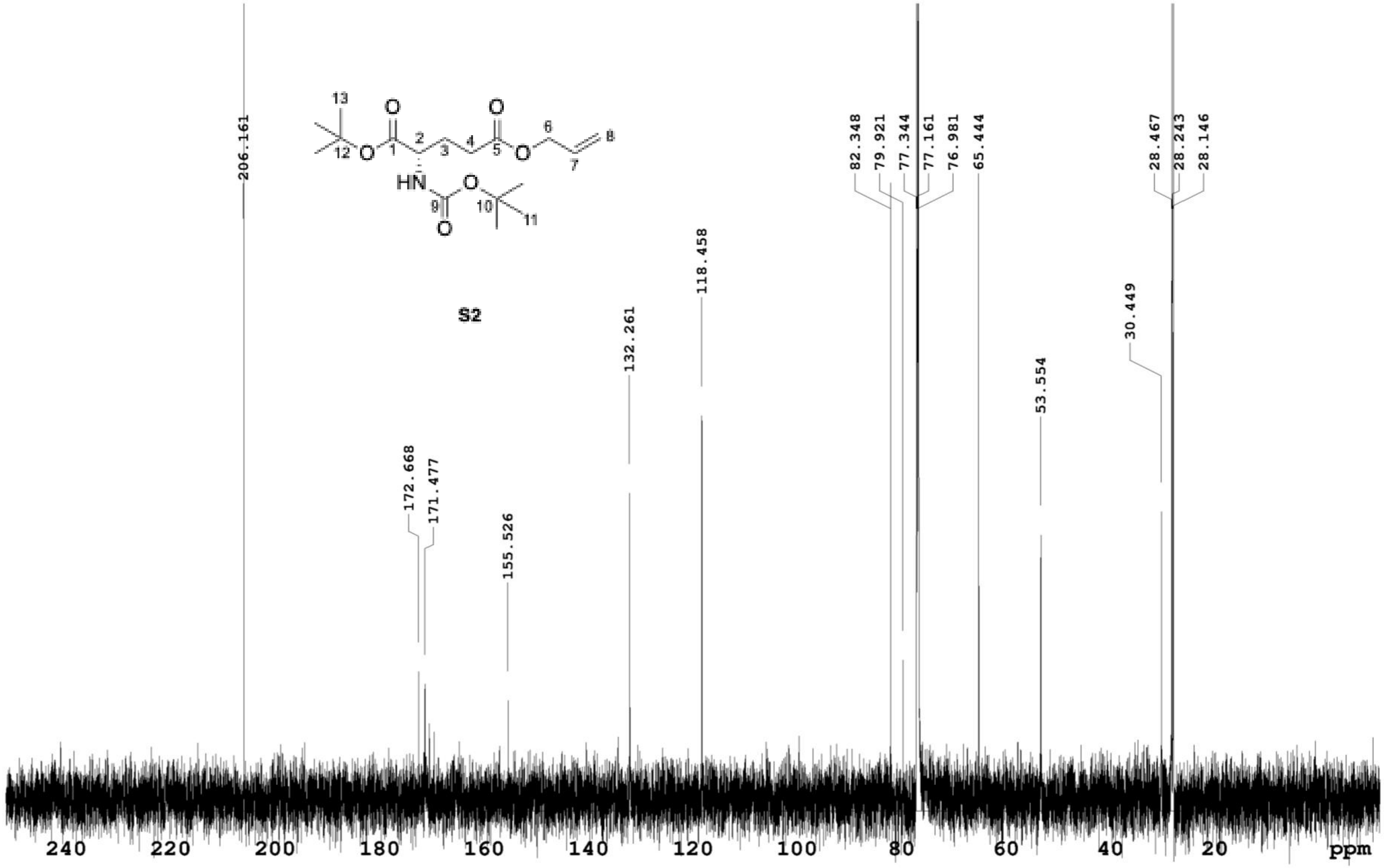
206.161
172.668
171.477
155.526
132.261
118.458
82.348
79.921
77.344
77.161
76.981
65.444
53.554
30.449
28.467
28.243
28.146
S2
240
220
200
180
160
140
120
100
80
60
40
20
ppm

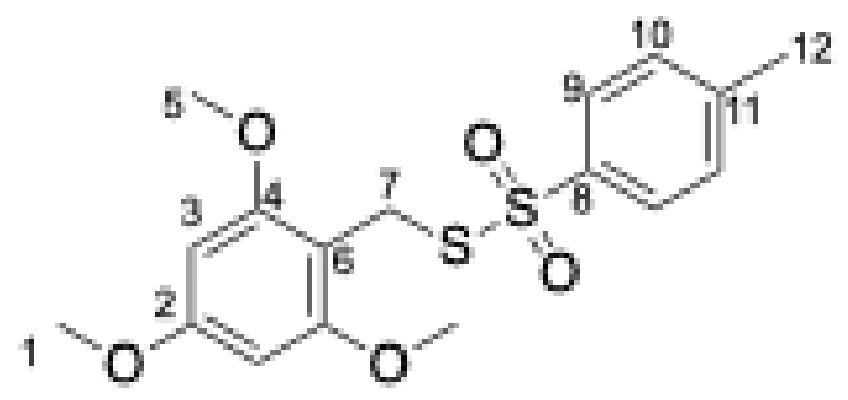

1
2
3
4
5
6
7
8
9
10
11
12
S
S
O
O
O
O
O

S3

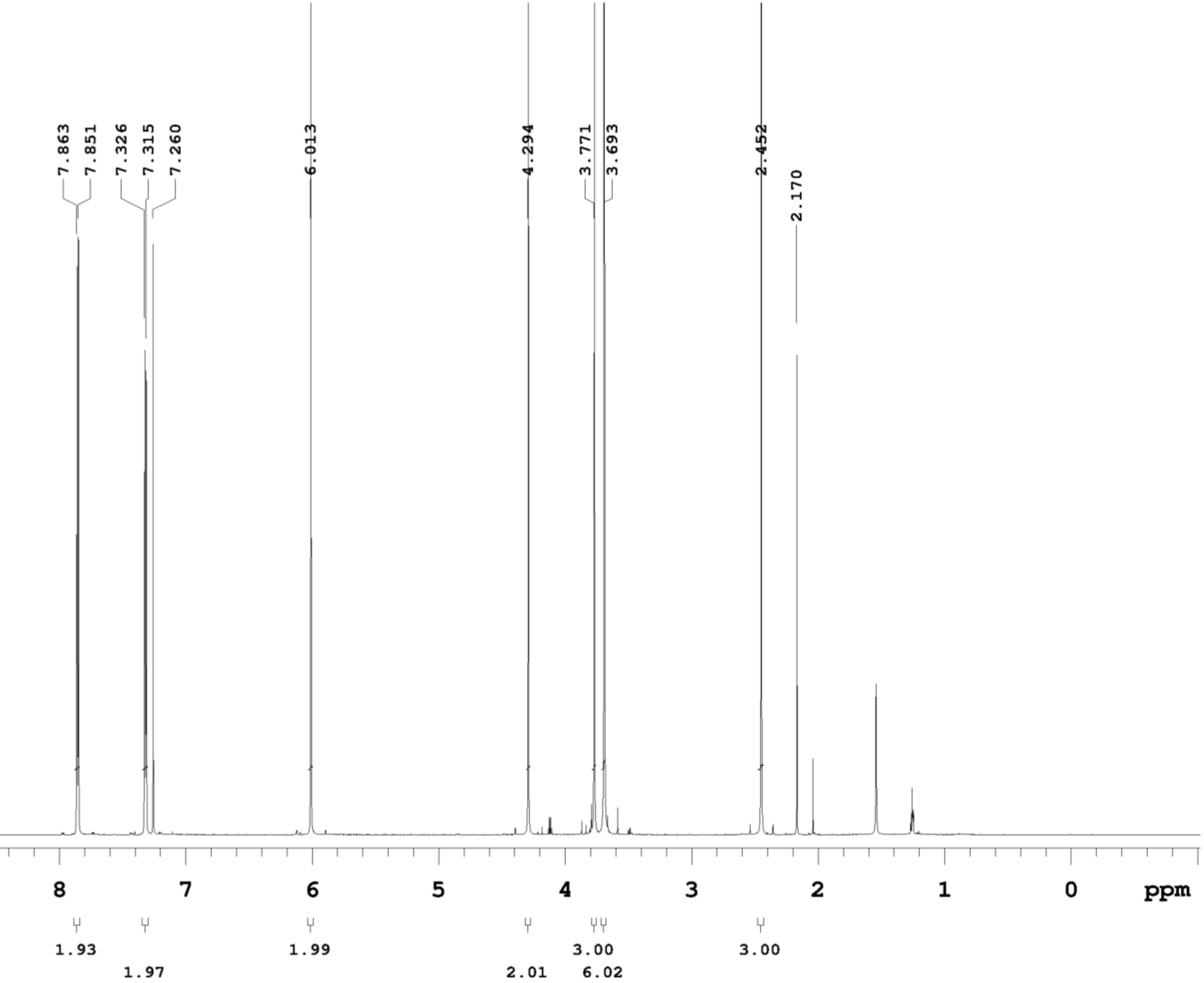

7.863
7.851
7.326
7.315
7.260
6.013
4.294
3.771
3.693
2.452
2.170
10
9
8
7
6
5
4
3
2
1
0
ppm
1.93
1.97
1.99
2.01
3.00
6.02
3.00

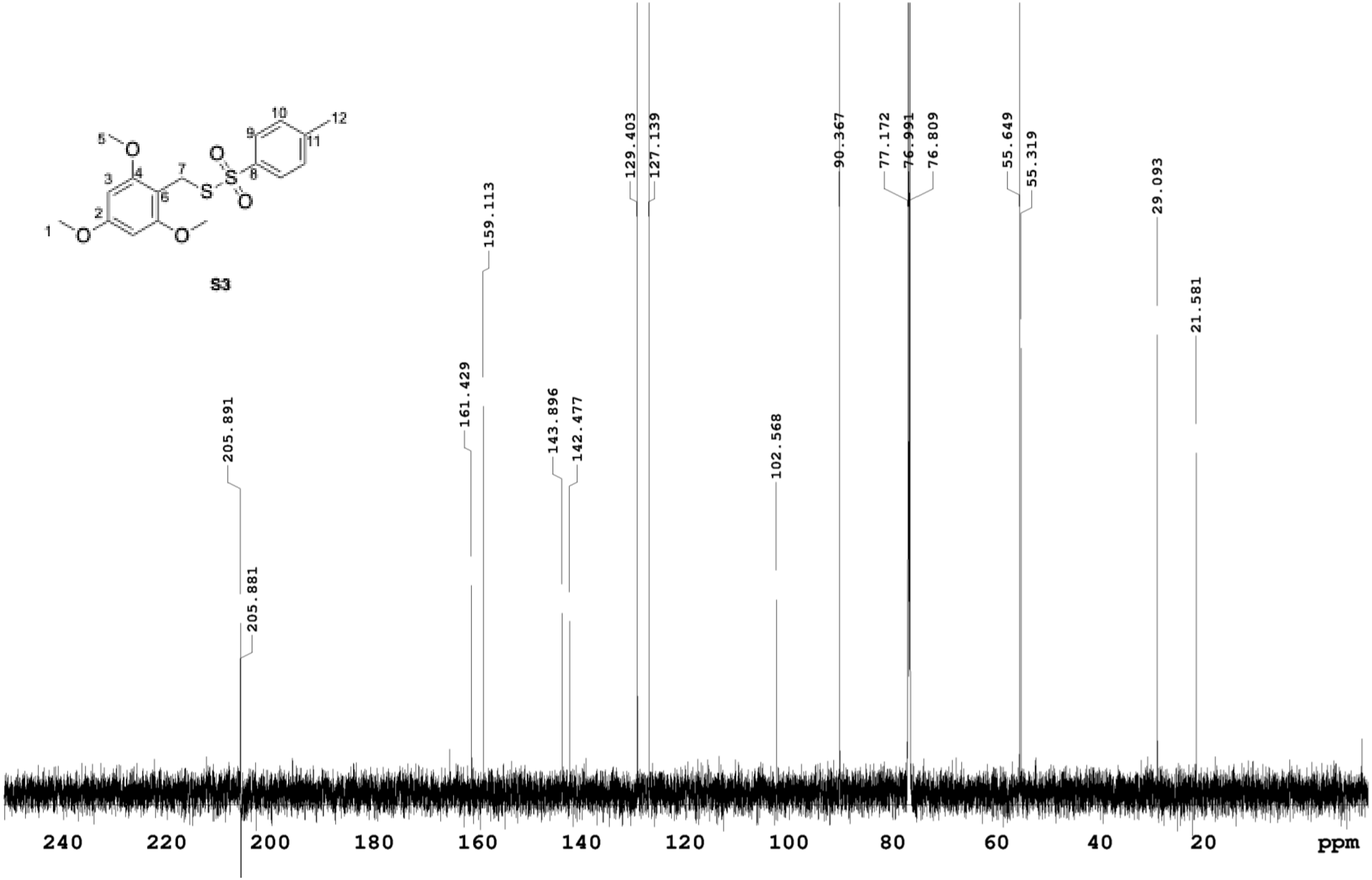
205.891
205.881
161.429
159.113
143.896
142.477
129.403
127.139
102.568
90.367
77.172
76.991
76.809
55.649
55.319
29.093
21.581
240
220
200
180
160
140
120
100
80
60
40
20
ppm

S3

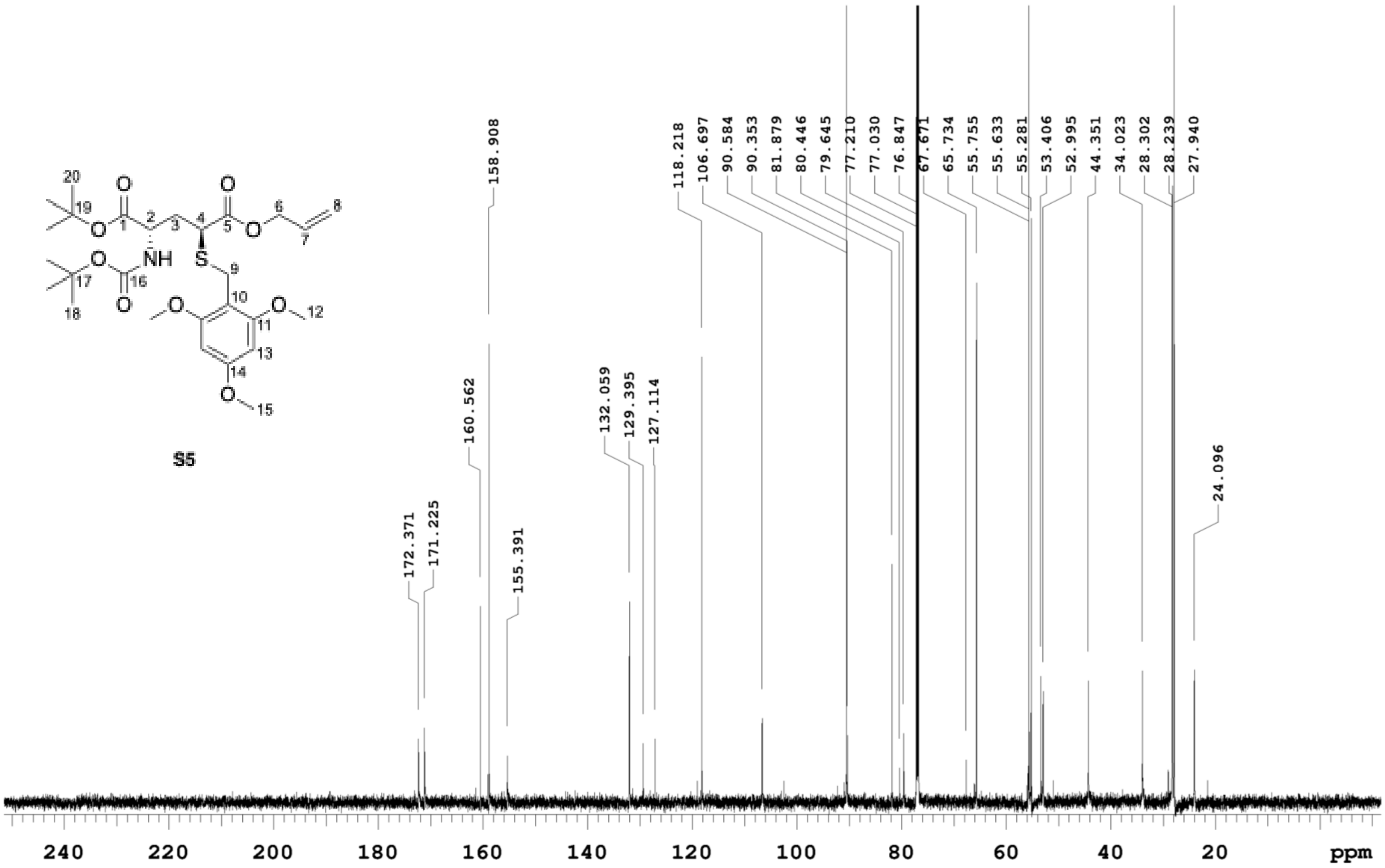

S5
172.371
171.225
160.562
158.908
155.391
132.059
129.395
127.114
118.218
106.697
90.584
90.353
81.879
80.446
79.645
77.210
77.030
76.847
67.671
65.734
55.755
55.633
55.281
53.406
52.995
44.351
34.023
28.302
28.239
27.940
24.096
240
220
200
180
160
140
120
100
80
60
40
20
ppm

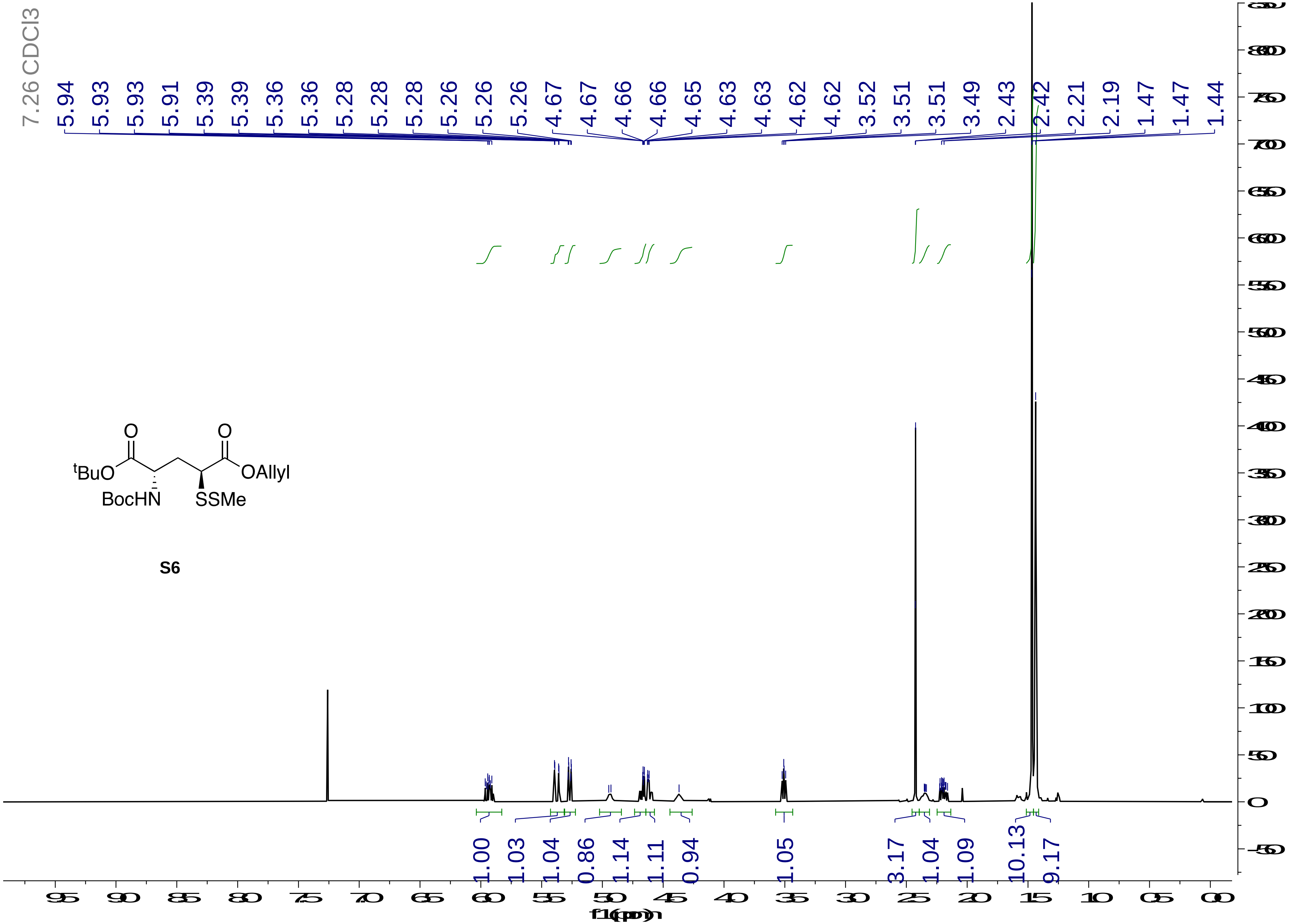

7.26 CDCl3
5.94
5.93
5.93
5.91
5.39
5.39
5.36
5.36
5.28
5.28
5.28
5.26
5.26
5.26
4.67
4.67
4.66
4.66
4.65
4.63
4.63
4.62
4.62
3.52
3.51
3.51
3.49
2.43
2.42
2.21
2.19
1.47
1.47
1.44
O
O
tBuO
OAllyl
BocHN
SSMe
1.00
1.03
1.04
0.86
1.14
1.11
0.94
1.05
3.17
1.04
1.09
10.13
9.17
f1 (ppm)

S6

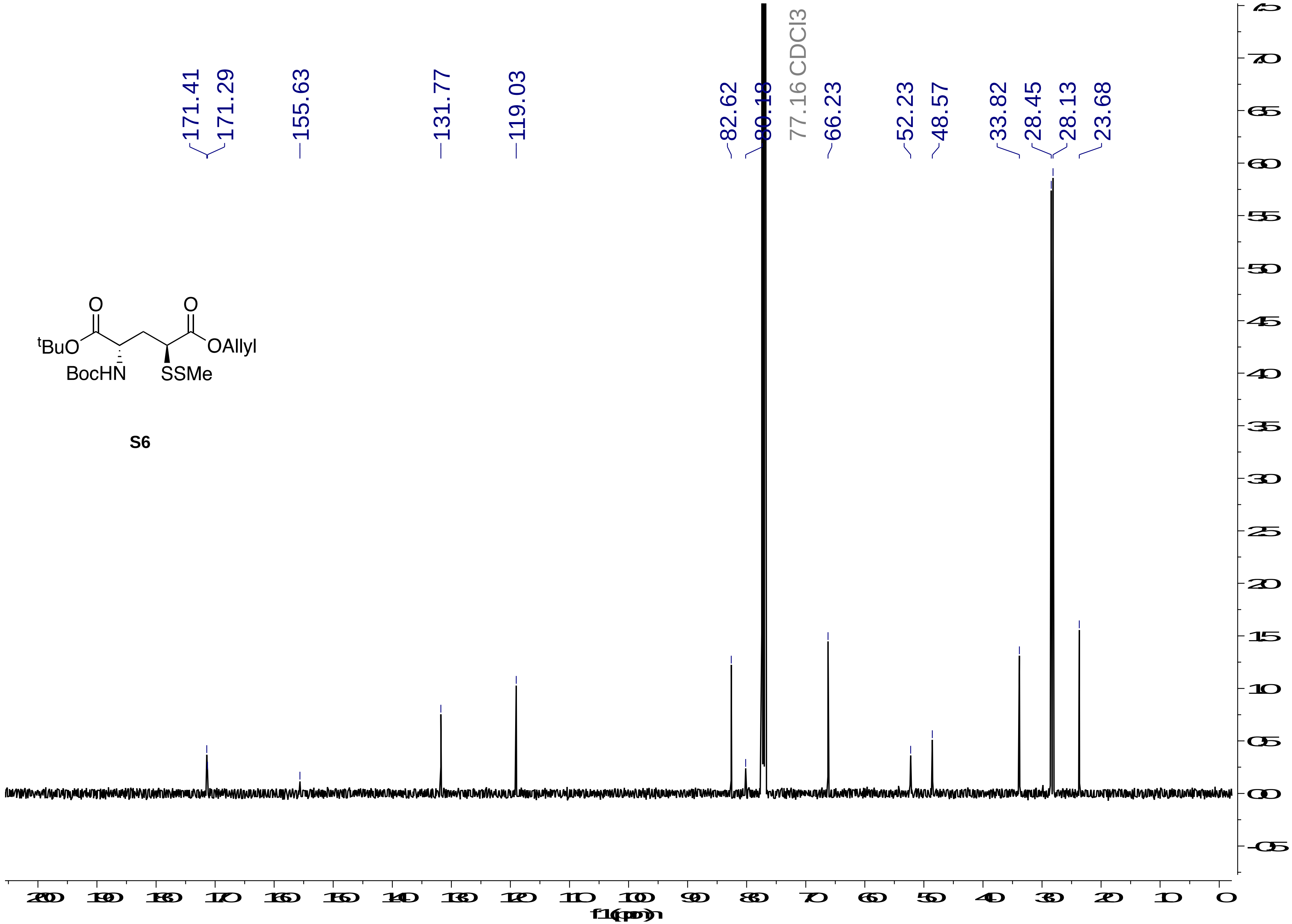
171.41
171.29
155.63
131.77
119.03
82.62
80.19
77.16 CDCl3
66.23
52.23
48.57
33.82
28.45
28.13
23.68
O
O
tBuO
OAllyl
BocHN
SSMe
S6
f1 (ppm)

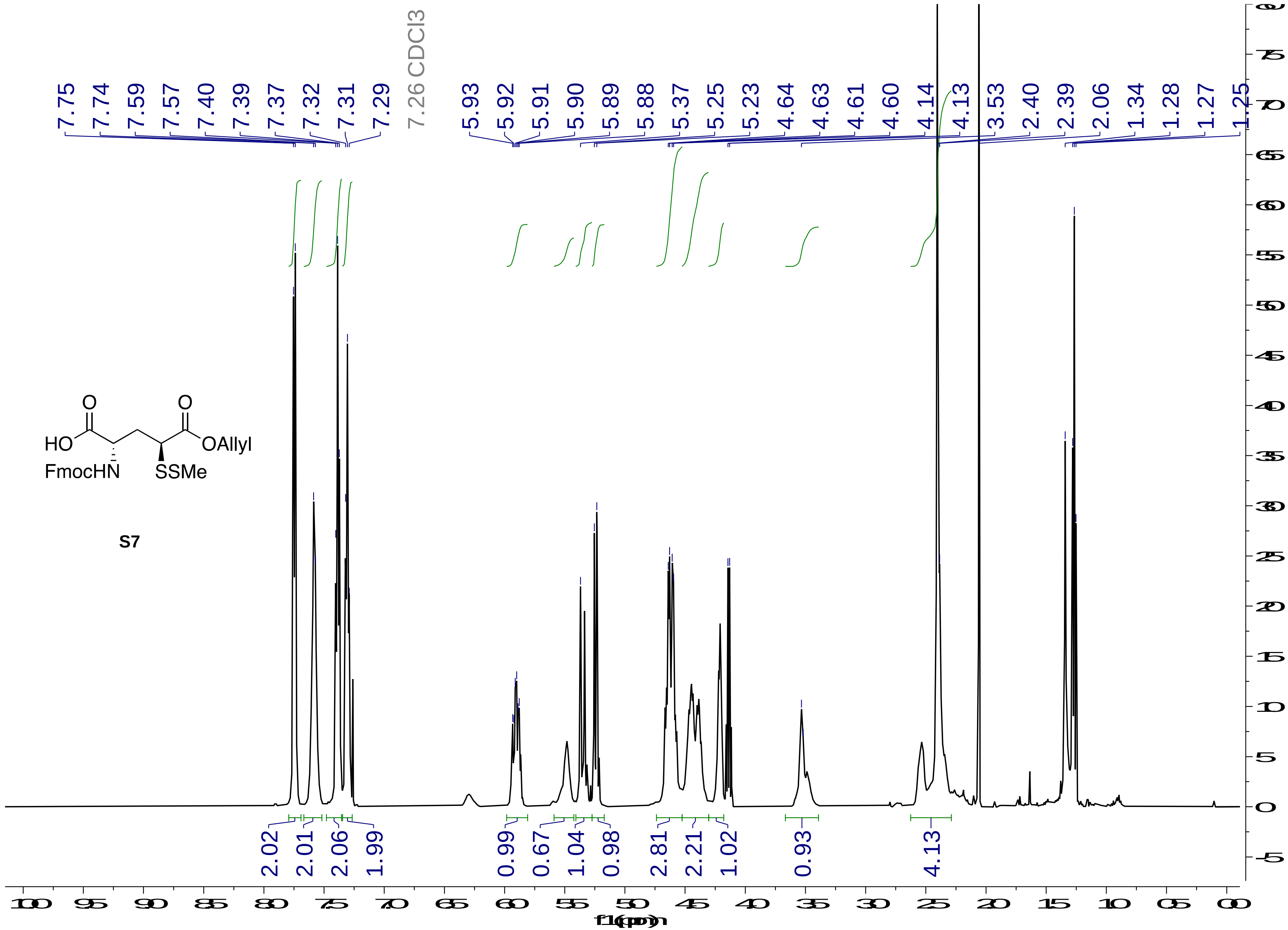

S7

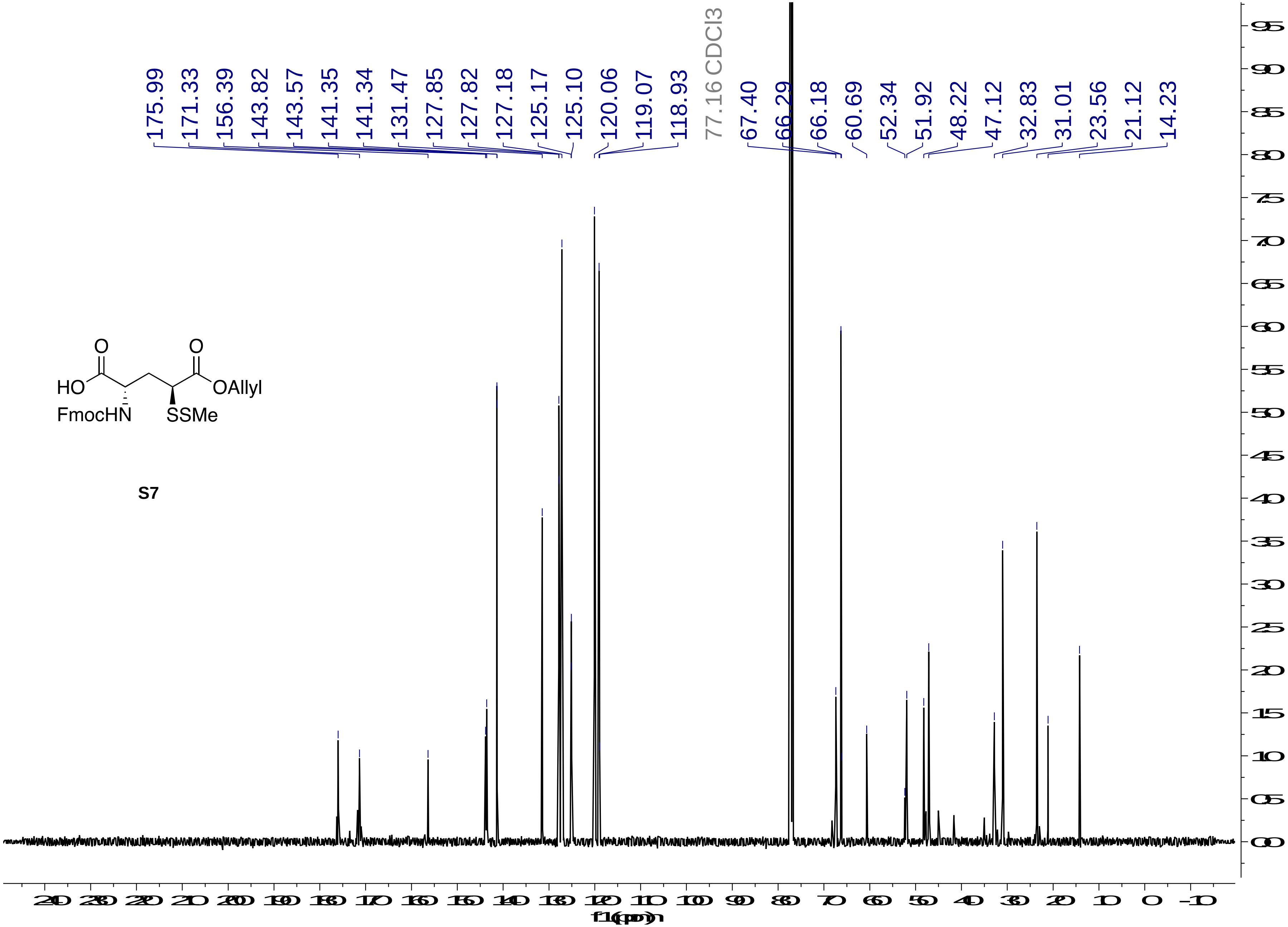
175.99
171.33
156.39
143.82
143.57
141.35
141.34
131.47
127.85
127.82
127.18
125.17
125.10
120.06
119.07
118.93
77.16 CDCl3
67.40
66.29
66.18
60.69
52.34
51.92
48.22
47.12
32.83
31.01
23.56
21.12
14.23
HO
O
O
OAllyl
FmocHN
SSMe
S7
f1 (ppm)
